# Excited state electron and energy relays in supramolecular dinuclear complexes revealed by ultrafast optical and X-ray transient absorption spectroscopy†
†Electronic supplementary information (ESI) available: Synthesis schemes, experimental methods, NMR spectra, X-ray crystallographic information, emission spectra, cyclic voltammetry, electronic structure calculations, data analysis and numerical methods, and other additional figures. CCDC 1561879. For ESI and crystallographic data in CIF or other electronic format see DOI: 10.1039/c7sc04055e


**DOI:** 10.1039/c7sc04055e

**Published:** 2017-11-28

**Authors:** Dugan Hayes, Lars Kohler, Ryan G. Hadt, Xiaoyi Zhang, Cunming Liu, Karen L. Mulfort, Lin X. Chen

**Affiliations:** a Chemical Sciences and Engineering Division , Argonne National Laboratory , Argonne , IL 60439 , USA . Email: dugan@uri.edu ; Email: mulfort@anl.gov ; Email: lchen@anl.gov; b X-ray Science Division , Argonne National Laboratory , Argonne , IL 60439 , USA; c Department of Chemistry , Northwestern University , Evanston , IL 60208 , USA

## Abstract

The kinetics of photoinduced electron and energy transfer in a family of tetrapyridophenazine-bridged heteroleptic homo- and heterodinuclear copper(i) bis(phenanthroline)/ruthenium(ii) polypyridyl complexes were studied using ultrafast optical and multi-edge X-ray transient absorption spectroscopies. This work combines the synthesis of heterodinuclear Cu(i)–Ru(ii) analogs of the homodinuclear Cu(i)–Cu(i) targets with spectroscopic analysis and electronic structure calculations to first disentangle the dynamics at individual metal sites by taking advantage of the element and site specificity of X-ray absorption and theoretical methods. The excited state dynamical models developed for the heterodinuclear complexes are then applied to model the more challenging homodinuclear complexes. These results suggest that both intermetallic charge and energy transfer can be observed in an asymmetric dinuclear copper complex in which the ground state redox potentials of the copper sites are offset by only 310 meV. We also demonstrate the ability of several of these complexes to effectively and unidirectionally shuttle energy between different metal centers, a property that could be of great use in the design of broadly absorbing and multifunctional multimetallic photocatalysts. This work provides an important step toward developing both a fundamental conceptual picture and a practical experimental handle with which synthetic chemists, spectroscopists, and theoreticians may collaborate to engineer cheap and efficient photocatalytic materials capable of performing coulombically demanding chemical transformations.

## Abstract 2

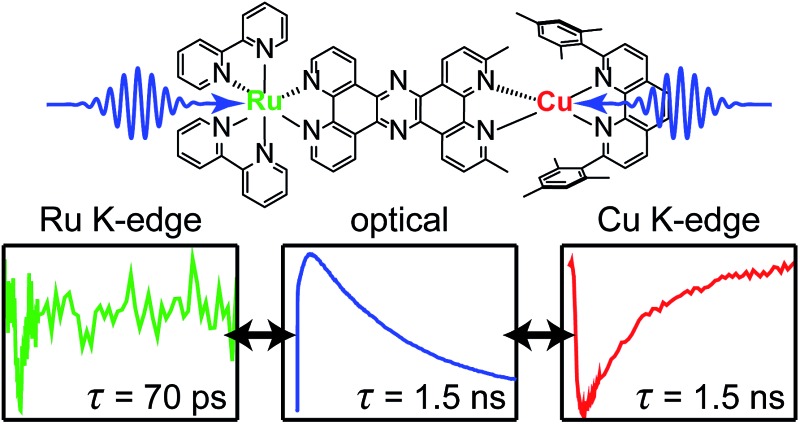
Complementary ultrafast techniques provide clear observation of charge hopping between metals in dinuclear complexes.

## Introduction

The rational design of multinuclear transition metal complexes for photochemical catalysis of homogeneous and/or heterogeneous multi-electron reactions (*e.g.* for producing solar fuels^1^–^4^) requires a detailed understanding of the often unique and convoluted excited state charge and energy transfer pathways and associated structural dynamics of these systems. Natural photosynthetic machineries, in which multiple chromophores and electron donors/acceptors are arranged in well-defined geometries to support long-lived, directional photoinduced charge separation,^5^–^9^ have provided inspiration for many such efforts,^10^–^18^ but effectively incorporating design principles from Nature into small molecule analogs remains a challenge. Recent synthetic efforts have established a variety of approaches for combining multiple light-absorbing and redox-active centers into linked assemblies toward the goal of developing homogeneous photocatalysts for multi-electron and/or multi-hole redox processes.^19^–^36^ But while ultrafast optical transient absorption spectroscopy (OTA) has been deployed extensively to map the evolution of electronic excited states in mononuclear transition metal complexes, obtaining a comprehensive picture of the dynamics of multinuclear complexes in the same fashion is often complicated by the spectroscopically indistinct nature of the various metal sites and the transfer of charges to and from shared ligands.

**Fig. 1:**
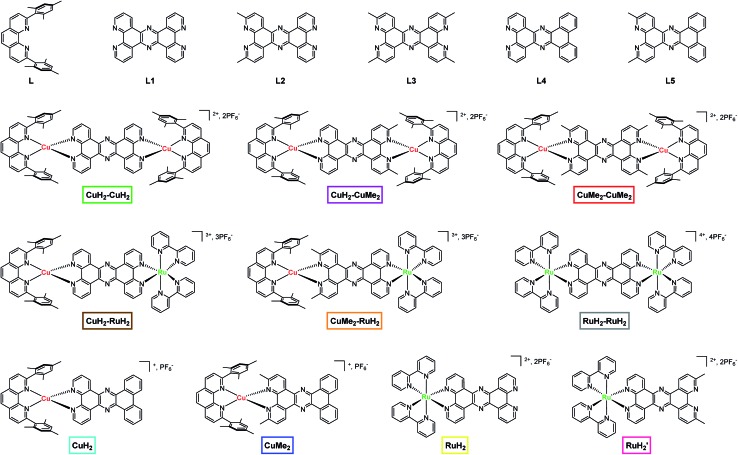
Chemical structures of ligands, dinuclear complexes, and mononuclear complexes studied in this work. The nomenclature and color scheme (boxes) introduced here is used throughout the text.

One particularly versatile method for assembling multiple metal centers using tetrapyrido[3,2-*a*:2′,3′-*c*:3′′,2′′-*h*:2′′′,3′′′-*j*]phenazine (tpphz) as a bridging ligand was first reported by Knapp *et al.*^37^ and Bolger *et al.*^38^,^39^ This ligand has been used as a building block for mono-,^38^ di-,^37^,^38^ tetra-,^40^–^42^ and polynuclear^37^,^43^ Ru(ii) constructs as well as stereochemically pure,^44^ asymmetric homodinuclear,^45^ topological,^46^ mixed valent,^47^ and heterodinuclear^48^–^53^ complexes. Additionally, the CuHETPHEN method pioneered by Schmittel *et al.*^54^,^55^ has been used by several groups,^56^–^69^ including our own,^70^,^71^ to prepare analytically pure heteroleptic Cu(i) bis(phen) complexes (phen = 1,10-phenanthroline) that can serve as individual building blocks in the piecewise assembly of supramolecular constructs with absolute synthetic control. In addition to furnishing synthetically bifunctional complexes, this design strategy can effectively facilitate unidirectional charge transfer by imposing local energetic asymmetry along possible charge separation pathways. In the current work, we incorporate tpphz-based bridging ligands into a CuHETPHEN synthetic scheme to obtain a family of mononuclear, symmetric and asymmetric homodinuclear, and heterodinuclear Cu(i)/Ru(ii) complexes (Fig. 1) as a first step toward building functional multimetallic photocatalysts.

Copper(i) diimine complexes have the potential to serve as earth-abundant substitutes for benchmark ruthenium(ii) tris(bpy) photosensitizers (bpy = 2,2′-bipyridine) in solar energy harvesting applications.^72^–^75^ Using ultrafast OTA, X-ray transient absorption (XTA), and fluorescence upconversion techniques, we and other groups have established a general scheme describing the excited state dynamics of these complexes, drawing correlations between ground/excited state structure and photophysical properties.^61^,^74^–^91^ One of the most attractive properties of these complexes is their broad, intense metal-to-ligand charge transfer (MLCT) absorption that closely resembles that of [Ru(bpy)_3_]^2+^, spanning a significant portion of the visible spectrum.^92^–^94^ Despite these similarities, however, a functionally important difference between Cu(i) and Ru(ii) complexes lies in their respective changes in nuclear geometry upon transition from the ground state to the MLCT state. While the photoinduced oxidation of Cu(i) (3d^10^) to Cu(ii)* (3d^9^) generally results in a significant pseudo Jahn–Teller distortion (JT) from a pseudo-tetrahedral geometry of *D*_2d_ symmetry to a flattened geometry of *D*_2_ symmetry, octahedral Ru(ii) polypyridyl complexes exhibit only very minor structural changes and boast much longer MLCT lifetimes. The extent of the distortion in Cu(i) complexes, however, varies substantially and is dictated by the steric and π-stacking interactions of the ligands. This process occurs in less than 1 ps, followed by intersystem crossing (ISC) from the ^1^MLCT to the ^3^MLCT state on the timescale of a few ps to a few tens of ps. Finally, relaxation to the ground state occurs on a timescale that varies from a few tens of ps to several μs, depending strongly on the identity of both the solvent and the substitution around the phenanthroline ligands.

Meanwhile, in their studies of the excited state dynamics of tpphz-bearing Ru(ii) complexes, Flamigni *et al.* found an intermediate (∼200 ps) time component following ISC that corresponds to relaxation from the ^3^MLCT_1_ to the ^3^MLCT_0_ state as the metal-derived electron migrates to the pyrazine-like center of tpphz.^95^ In a study of related Ru(ii)–tpphz compounds, Chiorboli *et al.* found that ground state recovery is accelerated by more than an order of magnitude upon metalation or protonation of the distal nitrogens of tpphz as a result of stabilization of the ^3^MLCT_0_ state.^50^ By incorporating these processes into the general scheme for Cu(i) bis(phen) complexes, we expect to obtain a complete description of the dynamics of tpphz-bearing mononuclear Cu(i) complexes. As we will demonstrate here, our data and calculations are indeed consistent with such a picture.

In general, however, the excited state dynamics of the multimetallic complexes employing tpphz as a bridging ligand cannot be modeled as simple linear combinations of the dynamics of the individual components. Instead, the dynamics of these and related systems must be considered as a network of competing charge transfer, energy transfer, and relaxation processes.^96^–^99^ And unfortunately for the ultrafast spectroscopist, the nearly indistinguishable optical absorption spectra of variously substituted Cu(i) bis(phen) and Ru(ii) tris(bpy) complexes can turn the task of deconvoluting time-resolved optical data – and thereby mapping such networks – into a nearly intractable problem. Nevertheless, Chiorboli *et al.* were able to conclude from steady-state emission^47^ and optical transient absorption measurements^50^ that intermetallic charge/energy transfer in a Ru(ii)–Os(ii) complex occurs with a weakly solvent-dependent time constant of ∼15 ps. Similarly, Torieda *et al.* observed photoinduced intramolecular electron transfer in a tpphz-bridged Ru(ii)–Co(iii) complex,^49^ and the mechanism of this process was recently elucidated by Canton *et al.* using a combination of ultrafast optical and X-ray spectroscopies.^51^,^100^

In this work we take advantage of the element specificity of X-ray absorption spectroscopy to separate the dynamics of two individual metal sites in a newly synthesized heterodinuclear copper/ruthenium complex through multi-edge X-ray transient absorption spectroscopy (XTA).^101^ We then compare these results with those obtained from optical transient absorption (OTA) and a series of electronic structure calculations to unambiguously construct a detailed picture of the evolution of the complex following excitation of the MLCT band. After establishing a model to describe the dynamics of the heterodinuclear system, we then apply these conclusions to the more challenging problem of an asymmetric homodinuclear Cu(i) complex. In this case, we find evidence for the transfer of excitation from the 3,6-dimethyl Cu(ii)* state with a ∼2 ns lifetime to the 3,6-unsubstituted Cu(ii)* state with a ∼40 ps lifetime on the timescale of ∼1 ns, demonstrating a potential means for indirectly extending the short excited state lifetimes of otherwise desirable sensitizer dyes without modifying coordination geometry or steady-state spectroscopic properties. We anticipate that this multi-disciplinary approach to mapping photoinduced charge transfer dynamics in the linked dinuclear light-absorbing complexes described here will provide a clear pathway forward for characterizing and designing larger multimetallic constructs capable of coupling single electron charge transfer events to multi-electron charge accumulation and redox catalysis.

## Results

### 1. Synthesis

The parent bridging ligand tetrapyrido[3,2-*a*:2′,3′-*c*:3′′,2′′-*h*:2′′′,3′′′-*j*]phenazine, labeled **L1** in Fig. 1 and the synthesis schemes in Section 1 of the ESI,† is well known in the literature.^37^–^39^,^44^ The synthesis of **L1** can be accomplished either by reacting 1,10-phenanthroline-5,6-dione with an excess of ammonium acetate or by condensing 1,10-phenanthroline-5,6-dione with one equivalent of 1,10-phenanthroline-5,6-diamine in the presence of acetic acid. The two new bridging ligands **L2** and **L3**, which respectively feature two and four methyl groups at the 3,6 and 3,6,12,15 positions in analogy to 2,9-dimethyl-1,10-phenanthroline, were prepared in a similar manner by condensation of the appropriately functionalized phenanthroline derivatives (Scheme S1†).

The dinuclear copper(i) complexes containing the three different bridging ligands were prepared using the CuHETPHEN approach originally developed by the Schmittel group.^54^,^55^ Briefly, [Cu(CH_3_CN)_4_]PF_6_ was mixed with one equivalent of the blocking ligand 2,9-dimesityl-1,10-phenanthroline (**L**) to form the intermediate [Cu(**L**)(CH_3_CN)](PF_6_). From this intermediate, the dinuclear complexes were obtained by one of two routes. The first is a direct reaction of two equivalents of [Cu(**L**)(CH_3_CN)](PF_6_) with one equivalent of the appropriate bridging ligand (**L1**, **L2**, or **L3**) to yield the corresponding dinuclear complexes **CuH_2_–CuH_2_**, **CuH_2_–CuMe_2_**, and **CuMe_2_–CuMe_2_**. The second method is to prepare fully coordinated CuHETPHEN intermediates by reacting [Cu(**L**)(CH_3_CN)](PF_6_) with one equivalent of 1,10-phenantholine-5,6-dione or 1,10-phenanthroline-5,6-diamine (or their 2,9-methyl substituted analogs). The mononuclear CuHETPHEN complexes functionalized with dione and diamine groups on the B-ring of the phenanthroline ligand can then be condensed in a 1 : 1 ratio in the presence of acetic acid to form the phenazine ring that bridges the two Cu(i)(**L**) centers.

Heterodinculear Cu–Ru complexes **CuH_2_–RuH_2_** and **CuMe_2_–RuH_2_** were synthesized using a route similar to that previously described by Bolger *et al.* (Scheme S2†).^39^ In the first step, the mononuclear ruthenium complexes **RuH_2_** and **RuH_2_′** were prepared by condensing [Ru(bpy)_2_(5,6-dione-1,10-phenanthroline)](PF_6_)_2_ with the appropriate 5,6-diamine-1,10-phenanthroline (with or without 2,9-methyl substitution). Importantly, during and following this reaction we did not observe the formation of any dinuclear complexes. In the second step, the mononuclear ruthenium complexes **RuH_2_** and **RuH_2_′** were converted to the heterodinuclear complexes **CuH_2_–RuH_2_** and **CuMe_2_–RuH_2_** by adding one equivalent of [Cu(**L**)(CH_3_CN)](PF_6_) to the mononuclear ruthenium complexes in dichloromethane.

The mononuclear Cu(i) and Ru(ii) analogs to the dinuclear complexes were synthesized as models for the spectroscopic analysis. Initial attempts at the synthesis of **CuH_2_** and **CuMe_2_** from a stoichiometric mixture of [Cu(**L**)(CH_3_CN)](PF_6_) and **L1**, **L2**, or **L3** yielded a mixture of the desired mononuclear complex and its dinuclear analog, which could not be separated. Similar mixtures were also found following condensation of [Cu(**L**)(5,6-dione-1,10-phenanthroline)](PF_6_) or [Cu(**L**)(5,6-diamine-1,10-phenanthroline)](PF_6_) with the complementarily functionalized phenanthroline. This is not entirely surprising given the solution lability of Cu(i) complexes and the propensity toward ligand scrambling in solution. Therefore, to ensure well-defined and pure solutions of each Cu(i) model complex, we replaced the two distal coordinating nitrogens of the bridging ligands **L1** and **L2** with carbons by designing and synthesizing phenazine ligands **L4** and **L5** (Scheme S3†). **L4** and **L5** were prepared *via* condensation of the appropriate 5,6-diamine-1,10-phenanthroline derivative with 9,10-phenanthrenequinone. The mononuclear complexes **CuH_2_** and **CuMe_2_** were then easily obtained by reaction of [Cu(**L**)(CH_3_CN)](PF_6_) with **L4** and **L5** under standard CuHETPHEN conditions.

### 2. Crystal structure of **CuH_2_–RuH_2_**

**Fig. 2:**
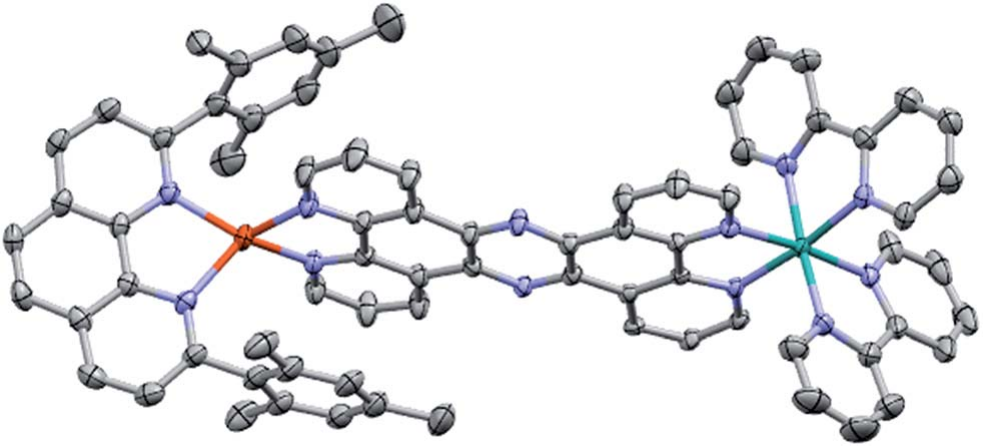
Crystal structure of **CuH_2_–RuH_2_**. Ellipsoids are depicted at 50% probability. Atom labels: carbon, gray; nitrogen, blue; copper, orange; ruthenium, green. Hydrogen atoms, counterions, and disordered solvent molecules are omitted for clarity.

The molecular structure of **CuH_2_–RuH_2_** was verified by single crystal X-ray crystallography. Single crystals of **CuH_2_–RuH_2_** were obtained by slow diffusion of diethyl ether into a saturated acetonitrile solution. Fig. 2 shows the X-ray structure of **CuH_2_–RuH_2_**; the crystallographic data are summarized in Table S1† and selected interatomic bond lengths and angles are listed in Table S2.† The Cu(i) side of the dinuclear complex is moderately disordered, and the solvent molecules (water, acetonitrile, and diethyl ether) are significantly disordered. The Ru–N bond lengths involving the two bipyridine ligands and the bridging ligand are within the normal range (2.04–2.07 Å) of what is expected for such bonds in [Ru(bpy)_2_(phenanthroline)]^2+^ type complexes.^102^,^103^ The Cu–N distances are 2.00–2.06 Å, in good agreement with bond lengths reported for related mononuclear CuHETPHEN complexes.^66^,^70^,^71^

An important feature of the CuHETPHEN side of **CuH_2_–RuH_2_** is the π-stacking interaction between one mesityl group of **L** and the Cu(i)-coordinating phenanthroline moiety of **L1**. This interaction leads to a significant distortion from ideal tetrahedral geometry around the Cu(i) center and creates a so-called “pac-man” motif, which has been described for related mononuclear CuHETPHEN complexes.^66^,^70^ Further analysis of the crystal structure of **CuH_2_–RuH_2_** reveals a substantial bend in **L1** instead of the perfectly planar geometry one might expect the fully conjugated bridging ligand to exhibit. This bend in **L1** results in a Cu–Ru distance of 12.64 Å, shorter than expected for a completely planar ligand. However, this is not unprecedented; bending in **L1** has also been reported in dinuclear structures bridging Ru(bpy)_2_ with AuCl_2_ or PdCl_2_.^52^,^53^ These complexes had only slightly longer Ru–M distances than what we observe for **CuH_2_–RuH_2_**: 12.74 Å for Ru–Au and 12.70 Å for Ru–Pd.

### 3. Ground state optical absorption and emission

**Fig. 3:**
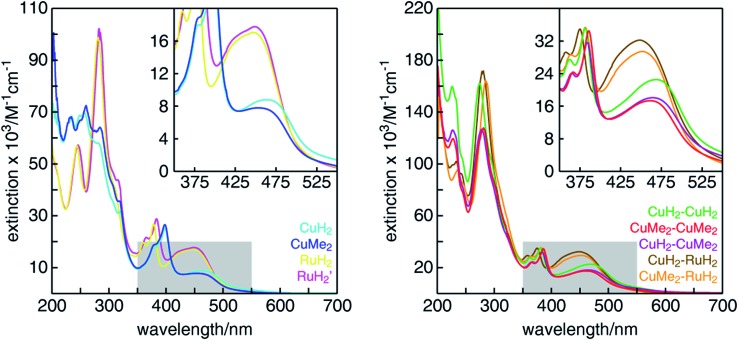
UV-visible absorption spectra of (left) mononuclear and (right) dinuclear Cu(i)/Ru(ii) diimine complexes in CH_3_CN. The insets show a zoom of the MLCT absorption bands in the region highlighted by gray boxes.

The UV-visible absorption spectra of all mononuclear and dinuclear complexes in acetonitrile are shown in Fig. 3 and summarized in Table 1. The spectra of the mononuclear complexes possess a fairly broad absorption band centered near 450 nm associated with MLCT, highlighted in the insets. As we have shown in mononuclear CuHETPHEN model complexes,^70^ the absorption maximum and intensity is dictated by the sterics around the Cu(i) center. In comparison to the unsubstituted **CuH_2_**, the MLCT absorption maximum of **CuMe_2_** at 452 nm is hypsochromically shifted by 12 nm and has an extinction coefficient ∼11% lower. In contrast, the MLCT bands of both mononuclear ruthenium complexes (**RuH_2_** and **RuH_2_′**) are similar in energy and intensity and therefore independent of substitution of the phenazine ligand at the 3,6 positions distal to the metal center. The extinction coefficients of the dinuclear complexes are very nearly a sum of the individual mononuclear counterparts (the ratio of the scales of the *y*-axes is 2 : 1). We note that the extinction coefficients of the heterodinuclear complexes (**CuH_2_–RuH_2_** and **CuMe_2_–RuH_2_**) are slightly larger than the sum of the analogous mononuclear complexes, which is likely due to the synthetic requirement to use **L4** and **L5** in the mononuclear copper complexes rather than the exactly analogous **L1**. At higher energies, all complexes possess the characteristic double-peaked feature between 350 and 400 nm corresponding to the n–π* and π–π* transitions of the extended tpphz-based ligand, and methylation of this bridging ligand leads to a hypsochromic shift of these features.

**Table 1:** Summary of electrochemical and optical ground state characterization in CH_3_CN^*a*^

	*λ*_max_, absorption (MLCT, nm)	*ε* (M^–1^ cm^–1^)	*E* (Cu^2+/+^) (V *vs.* SCE)	*E* (Ru^3+/2+^) (V *vs.* SCE)	*λ*_max_, emission (nm)	*E*_00_ (eV)	*E* (M^*n*+/(*n*–1)+^*) (V *vs.* SCE)
**CuH_2_**	464	8789	+0.52	—	—	—	—
**CuMe_2_**	452	7796	+0.90	—	671 (w)	2.15	–1.25
**RuH_2_**	447	17 049	—	+1.33^*b*^	616 (str)^*b*^	2.26	–0.93
**RuH_2_′**	449	17 756	—	+1.34	610 (str)	2.24	–0.90
**CuH_2_–CuH_2_**	469	22 526	+0.58	—	—	—	—
**CuH_2_–CuMe_2_**	465	18 077	+0.57; +0.88	—	—	—	—
**CuMe_2_–CuMe_2_**	461	17 354	+0.89	—	—	—	—
**CuH_2_–RuH_2_**	449	32 209	+0.55	+1.33	615	2.26	–0.93
**CuMe_2_–RuH_2_**	452	29 426	+0.89	+1.34	612	2.24	–0.90
**RuH_2_–RuH_2_**	442^*b*^	36 100^*b*^	—	+1.34^*b*^	671^*b*^	—	—

^*a*^w = weak, str = strong.

^*b*^ *Inorg. Chem.*, 1996, **35**, 2937.

The room temperature emission of all complexes was measured in acetonitrile and is summarized in Table 1 and shown in Fig. S42.† As has been observed for mononuclear CuHETPHEN^70^ and homoleptic^74^ Cu(i)diimine complexes, substitution immediately adjacent to the Cu(i) center has a substantial effect on the emission properties. **CuH_2_**, bearing no substituents on the phenazine ligand, is completely non-emissive at room temperature in acetonitrile, but increasing the steric bulk around the Cu(i) center by introducing methyl groups in **CuMe_2_** leads to a weak emission peak with a maximum at 671 nm. As for most Ru(ii)tris(bipyridyl) complexes, both mononuclear ruthenium complexes studied here have a strong emission response following MLCT excitation. Remote methyl substitution on the bridging ligand (**L1***vs.***L2**) leads to a slight hypsochromic shift of the emission maximum from 616 nm for **RuH_2_** to 610 nm for **RuH_2_′**. The heterodinuclear complexes are emissive at room temperature in acetonitrile, albeit with lower intensity than the mono- and dinuclear **RuH_2_** species. All three dinuclear copper complexes are non-emissive at room temperature in acetonitrile.

### 4. Cyclic voltammetry

Cyclic voltammetry was performed on each complex in acetonitrile to measure the redox potentials of the metal centers and the ligand-based reductions in the bridged dinuclear complexes. As with previously reported mononuclear CuHETPHEN complexes,^70^ we found that the Cu(ii/i) redox potential in **CuH_2_** and **CuMe_2_** is significantly influenced by substitution around the periphery of the 1,10-phenanthroline moiety of the tpphz bridging ligand (Fig. S43,† summarized in Table 1). Without any substituents at the 3,6-positions, the Cu(i) center of **CuH_2_** is most easily oxidized at +0.52 V *vs.* SCE. Increasing the steric bulk by introducing methyl groups at the 3,6-positions in **CuMe_2_** drives the redox potential 380 mV higher to +0.90 V *vs.* SCE. Both complexes exhibit a quasi-reversible Cu(ii/i) couple arising from a structural change that occurs upon oxidation and also likely from coordination of an acetonitrile solvent molecule that increases the coordination number from four to five, as suggested by our previous *in situ* electrolysis of Cu(i)bis(2,9-dimethylphenanthroline) to its Cu(ii) species.^104^ In the mononuclear complexes **RuH_2_** and **RuH_2_′**, the Ru(iii/ii) potential is not influenced by distal substitution on the phenazine ligand, as both possess reversible couples around +1.33 V *vs.* SCE. The substituents on the bridging ligand (**L1**, **L2**) are too far removed from the metal center to have any measureable influence on the Ru(iii/ii) potential.

We also used cyclic voltammetry to measure the ruthenium- and copper-centered redox potentials of all dinuclear complexes (Fig. S44†) and observed trends similar to those described for the mononuclear Ru and Cu complexes. The Ru(iii/ii) potential is not affected by distal methyl substitution of the bridging ligand or by the coordination of a second metal, appearing around +1.33 V *vs.* SCE as before. The Cu(ii/i) redox potentials of the unsubstituted CuHETPHEN part of the dinuclear complexes are found to be +0.57 ± 0.02 V *vs.* SCE in all homo- and heterodinuclear complexes (**CuH_2_–RuH_2_**, **CuH_2_–CuH_2_** and **CuH_2_–CuMe_2_**). These values are slightly more positive than what was found for the mononuclear complex **CuH_2_**, a minor perturbation perhaps resulting from a distant steric effect of the additional metal center. Increasing the steric bulk around the Cu(i) center resulted in a more positive Cu(ii/i) redox potential of +0.89 ± 0.01 V *vs.* SCE for **CuMe_2_–RuH_2_**, **CuH_2_–CuMe_2_** and **CuMe_2_–CuMe_2_**, comparable to the potential found for **CuMe_2_**. Interestingly, all methyl substituted CuHETPHEN complexes show a perfectly reversible Cu(ii/i) couple, whereas all unsubstituted complexes exhibit irreversibility.

The excited state reduction potentials *E*(M^*n*+/(*n*–1)+^*) were estimated by subtracting the onset of the emission band *E*_00_ from the ground-state oxidation potential *E*(M^*n*+/(*n*–1)+^); these values are collected in Table 1. The excited state reduction potential for **CuMe_2_** is –1.25 V *vs.* SCE, similar to that reported previously for CuHETPHEN model complexes.^70^,^71^ Both mononuclear ruthenium complexes are weaker excited state reductants than the CuHETPHEN counterparts with values around –0.90 V (–0.90 V for **RuH_2_** and –0.93 V for **RuH_2_′**) and show almost no substitution effect. Since the emission spectra of the heterodinuclear complexes **CuH_2_–RuH_2_** and **CuMe_2_–RuH_2_** closely resemble that of **RuH_2_**, we used the Ru(iii/ii) oxidation potential for the calculation of the heterodinuclear excited state reduction potentials listed in Table 1, obtaining values identical to those of the mononuclear ruthenium counterparts. However, even though we do not detect copper-based emission from **CuH_2_–RuH_2_** or **CuMe_2_–RuH_2_**, we presume that the excited state reduction potential of the copper half of the dinuclear complexes is also largely unchanged in the heterodinuclear complexes and could be used to drive more challenging electron transfer chemistry.

### 5. Electronic structure calculations

**Fig. 4:**
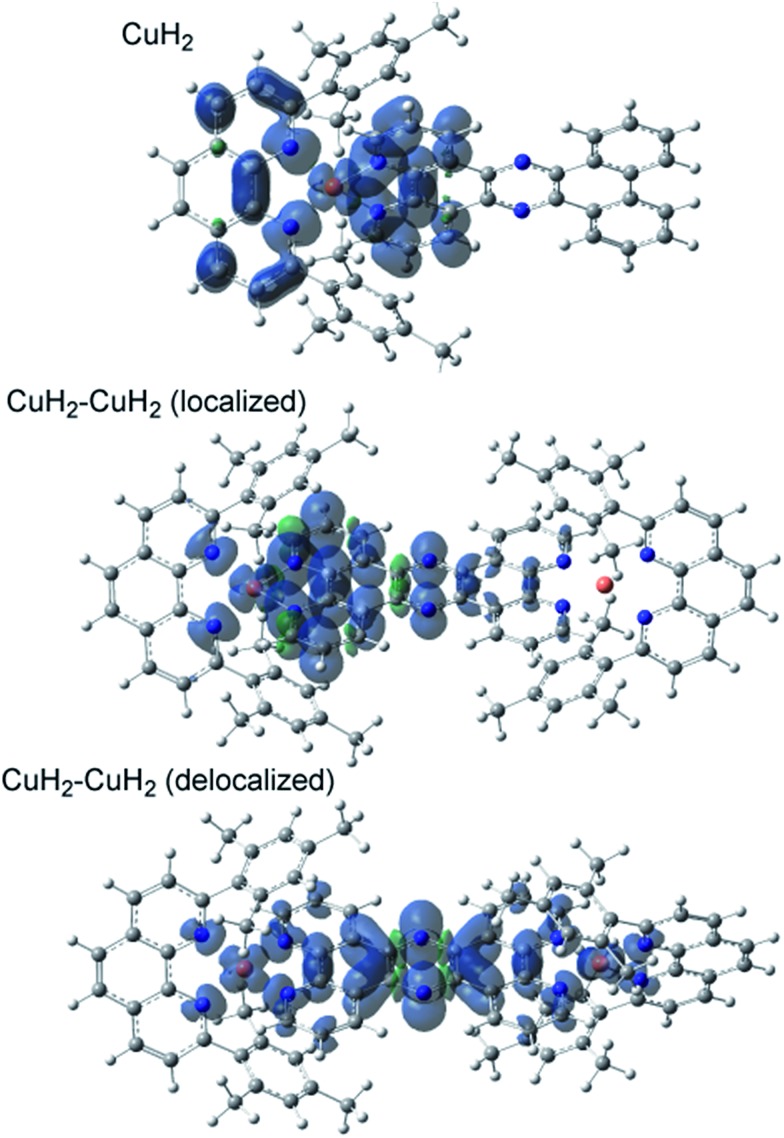
Triplet state spin-density plots for **CuH_2_** and **CuH_2_–CuH_2_** in both localized and delocalized states. Shading: α spin, blue; β spin, green. Atom labels: carbon, gray; nitrogen, blue; hydrogen, white; and copper, pink.

The TD-DFT calculated absorption spectra of **CuH_2_**, **CuMe_2_**, **CuH_2_–CuH_2_**, and **CuH_2_–RuH_2_** are compared to experimental data in Fig. S45,† and good agreement between theory and experiment is observed across these structural perturbations. The individual states and their donor and acceptor orbitals and assignments are given in Tables S3–S6.† Donor and acceptor orbital plots are also given in Fig. S46–S49.† From the calculated acceptor orbitals, the MLCT excited states of **CuH_2_** and **CuMe_2_** are seen to be delocalized onto both the phenanthroline moiety of the bridging ligand and the blocking ligand (**L**). This is also consistent with the spin density plots of the fully optimized ^3^MLCT states of these complexes, as shown in Fig. 4. However, when an additional metal is bound to the tpphz ligand, the acceptor orbitals localize to the tpphz ligand (Fig. S48 and S49†). Again, this is also consistent with spin density plots for the ^3^MLCT states in Fig. 4. We note that both localized and delocalized (from the Cu perspective) ^3^MLCT states could be converged and independently optimized. Using the B3LYP functional, the localized wavefunction and geometry is ∼1 kcal mol^–1^ lower in energy than the delocalized analog. For both cases, electron density is localized on the tpphz ligand, with significant pyrazine character.

In addition to TD-DFT calculated spectra, the singlet ground state-^3^MLCT energy gaps have been calculated from the difference in energy between the fully optimized geometries of **CuH_2_**, **CuMe_2_**, **CuH_2_–CuH_2_**, and **CuH_2_–RuH_2_** and are 1.82, 1.91, 1.67, and 1.42 eV, respectively. These differences in energy gaps are qualitatively consistent with the experimental lifetimes and the energy gap law. Going from **CuMe_2_** to **CuH_2_**, the lifetime decreases, as does the energy gap (1.91 to 1.82 eV, respectively). Additionally, binding a second metal decreases both the lifetimes and the energy gaps (1.92 to 1.67 and 1.42 eV for **CuH_2_** to **CuH_2_–CuH_2_** and **CuH_2_–RuH_2_**, respectively).

### 6. Optical transient absorption

**Fig. 5:**
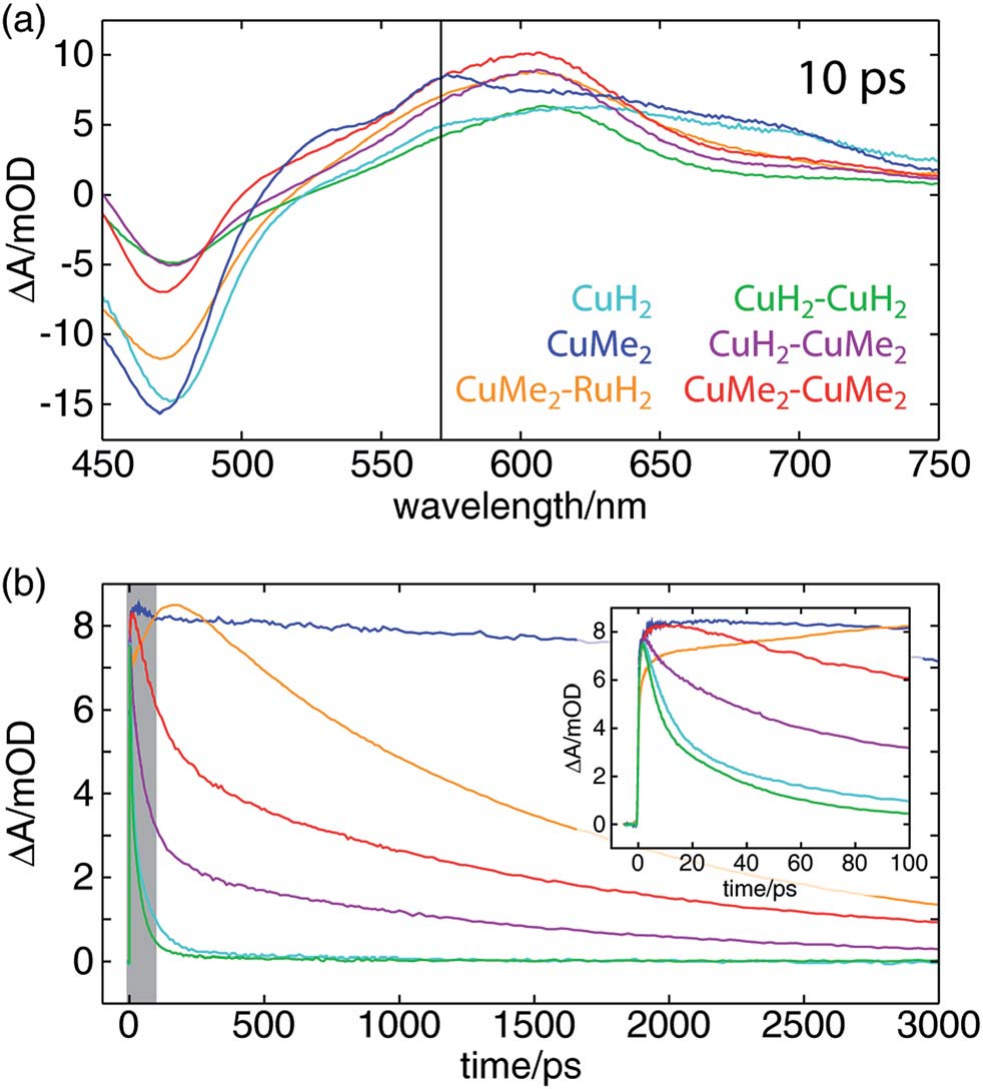
(a) OTA spectra of a series of heteroleptic Cu(i) bis(phen) complexes 10 ps after excitation of the MLCT band at 415 nm. The probe wavelength used to obtain the kinetic traces in panel (b) is indicated by a vertical black line. (b) OTA kinetic traces of the same complexes taken at a probe wavelength of 570 nm. The window plotted in the inset is indicated by a gray box. (Inset) A zoom of the early-time behavior of the OTA kinetic traces. The amplitude of the **CuMe_2_–RuH_2_** trace is clearly still rising at 100 ps, indicative of intermetallic charge transfer on that timescale.

The OTA spectra of the mono- and dinuclear copper complexes and one of the heterodinuclear complexes at a delay time of 10 ps following excitation at 415 nm are shown in Fig. 5a. The mononuclear **CuH_2_** (cyan) and **CuMe_2_** (blue) spectra show the familiar pair of excited state absorption (ESA) peaks at 525 and 575 nm common to homo- and heteroleptic Cu(i) bis(phen) complexes that have previously been assigned to absorption by the phenanthroline radical anion and the corresponding vibronic progression.^105^ These spectra also exhibit a broad ESA feature extending from 600 nm into the near-infrared that is not shared by the corresponding bis(phen) complexes, suggesting a tpphz radical anion provenance. The dinuclear **CuH_2_–CuH_2_** (green), **CuH_2_–CuMe_2_** (magenta), and **CuMe_2_–CuMe_2_** (red) spectra overlap well, sharing a broad ESA feature consisting of multiple incompletely resolved peaks and a negative ground state bleach (GSB) feature shallower than that observed in the mononuclear spectra. The **CuMe_2_–RuH_2_** also shares this broad ESA feature but shows a GSB more consistent with those of the mononuclear species.

Time traces of the OTA spectra taken at a probe wavelength of 570 nm (indicated by the vertical black line in Fig. 5a) are shown in Fig. 5b, and the corresponding exponential time constants (*vide infra*) are collected in Table 2. As in our previous report on a series of heteroleptic (**L**)Cu(i)(phen) complexes, 3,6-dimethyl substitution of the bridging ligand significantly increases the excited state lifetime of the mononuclear complex, in this case by more than two orders of magnitude (**CuMe_2_***vs.***CuH_2_**). Additionally, in comparing the mononuclear complexes to their respective symmetric dinuclear counterparts, we observe an acceleration in recovery of the ground state for the latter. While this acceleration is only marginal for the compounds bearing **L1**, the excited state lifetime of the dinuclear **CuMe_2_–CuMe_2_** species is 19 times shorter than that of the mononuclear **CuMe_2_**. The lifetime of the heterodinuclear **CuMe_2_–RuH_2_** complex is comparable to that of **CuMe_2_–CuMe_2_**, despite the presumptive partial excitation of the usually much longer lived ruthenium-centered MLCT. Similar dynamics have been reported by Scandola and coworkers in a series of mono- and dinuclear ruthenium and osmium complexes, for which excited state lifetimes were found to decrease by more than an order of magnitude in going from mononuclear to symmetric dinuclear species.^47^ Notably, this behavior was attributed to metalation of the distal binding site of **L1** rather than metal–metal interactions, as a similar decrease in lifetime was observed upon protonation of the nitrogens in the mononuclear species. Nevertheless, the **CuMe_2_–RuH_2_** lifetime is two orders of magnitude shorter than similar Ru(ii)–Ru(ii) dinuclear complexes.

**Table 2:** Summary of time constants measured by OTA and/or XTA in CH_3_CN with 415 nm excitation

	*τ*_1_, ISC/JT (ps)	*τ*_2_, ILET (ps)	*τ*_3_, IMCT (ps)	*τ*_4_, ^3^MLCT_0_ (ps)
**CuH_2_**	1.1 ± 0.1^*a*^	8.5 ± 0.6	N/A	82 ± 4
**CuMe_2_**	n.m.	170 ± 30	N/A	32 500 ± 500^*b*^
**CuH_2_–CuH_2_**	0.9 ± 0.2^*a*^	4.7 ± 0.2	N/A	38 ± 4
**CuH_2_–CuMe_2_**	0.8 ± 0.2^*a*^	4.3 ± 0.4; 140 ± 30	n.m.	47 ± 6; 1460 ± 60^*a*^
**CuMe_2_–CuMe_2_**	n.m.	120 ± 20	N/A	1720 ± 50
**CuH_2_–RuH_2_**	0.6 ± 0.1^*a*^	2.4 ± 0.2	21 ± 3	73 ± 1
**CuMe_2_–RuH_2_**	5.2 ± 0.5	35 ± 4	53 ± 5	1430 ± 30
**RuH_2_–RuH_2_**	n.m.	n.m.	N/A	70 000 ± 1000

^*a*^Tentative assignments.

^*b*^From XTA only, n.m. = not measured, N/A = not applicable.

We also find a clear trend within the series of dinuclear copper complexes, with **CuH_2_–CuMe_2_** exhibiting an excited state lifetime that falls between those of **CuH_2_–CuH_2_** and **CuMe_2_–CuMe_2_**. This stands in contrast not only to our own results for **CuMe_2_–RuH_2_** and **CuMe_2_–CuMe_2_** but also to the results reported by Scandola and coworkers. In that work, the asymmetric Ru(ii)–Os(ii) dinuclear complex exhibits dynamics identical to those of the faster symmetric Os(ii)–Os(ii) species.^50^ This apparent disparity, however, may be resolved upon consideration of the ^3^MLCT energies and reorganization energies of the relevant metal centers in the context of intermetallic charge transfer (IMCT). In such a picture, the excited state fraction of an asymmetric dinuclear sample consists of a mixture of two states, each bearing one of two possible oxidized metal sites following photoinduced electron transfer to the bridging tpphz ligand. One corresponds to the thermodynamically favored excited state and simply exhibits ground state recovery kinetics similar to those of the matching symmetric dinuclear complex. In the other, however, IMCT competes with relaxation to the ground state, and the overall behavior depends upon the relative rates of these two processes.

In the case of **CuMe_2_–RuH_2_** (reported here) and the Ru(ii)–Os(ii) complex reported by Scandola and coworkers,^50^ Ru(iii) is the stronger oxidant. Accordingly, hole transfer occurs from Ru(iii)* to Cu(i) or Os(ii) when the Ru(ii) MLCT band is excited, but IMCT does not occur when the Cu(i) or Os(ii) center absorbs a photon. This behavior is evident from the OTA data plotted in Fig. 5b. The **CuMe_2_–RuH_2_** ESA time trace (orange) exhibits both impulsive (<300 fs) and non-impulsive growth, only reaching its maximum after 160 ps. These two growth terms may be assigned to direct excitation of the Cu(i) and Ru(ii) sites and hole transfer from Ru(iii)* to Cu(i), respectively. Beyond 200 ps, however, **CuMe_2_–RuH_2_** and **CuMe_2_–CuMe_2_** (red) follow the same trajectory, relaxing to the ground state with a time constant of ∼1.5 ns.

We pause here to note the functional equivalence of hole and energy transfer in this particular class of complexes. According to our time-dependent density functional theory (TD-DFT) calculations (*vide supra*), the electron lost by either metal upon MLCT excitation of the dinuclear complexes resides on the bridging tpphz ligand, and therefore intermetallic hole transfer yields the same final state as would be achieved by energy transfer between the two ^3^MLCT states. Thus, relaxation of **CuMe_2_–RuH_2_** following IMCT is expected to resemble that of **CuMe_2_–CuMe_2_**. Additionally, because the lifetime of the Ru(ii) ^3^MLCT state is three orders of magnitude longer than the timescale of IMCT, direct relaxation from the Ru(iii)* state is not expected to significantly modulate the observed dynamics.

Elucidating the dynamics of the asymmetric homodinuclear **CuH_2_–CuMe_2_** complex presents a much more challenging problem. The steady state and ground state optical and X-ray absorption spectra of the unsubstituted and 3,6-dimethyl copper centers overlap very closely, making direct spectroscopic discrimination of the two sides difficult. Furthermore, while the Ru(iii)/Ru(ii) reduction potential (+1.34 V *vs.* SCE) is much higher than both Cu(ii)/Cu(i) reduction potentials in the heterodinuclear complexes (+0.55 and +0.89 V *vs.* SCE for **CuH_2_–RuH_2_** and **CuMe_2_–RuH_2_**, respectively), the difference in redox potentials between the two copper sites in **CuH_2_–CuMe_2_** is only 310 mV. This difference is expected to be comparable and opposite in sign to the difference in reorganization energies between the Cu(ii)H_2_*–Cu(i)Me_2_ and the Cu(i)H_2_–Cu(ii)Me_2_* states, complicating prediction of the rate and directionality of IMCT. Accordingly, we will return to a discussion of **CuH_2_–CuMe_2_** below only after a thorough analysis of the symmetric homodinuclear and heterodinuclear complexes.

### 7. Multi-edge X-ray transient absorption

**Fig. 6:**
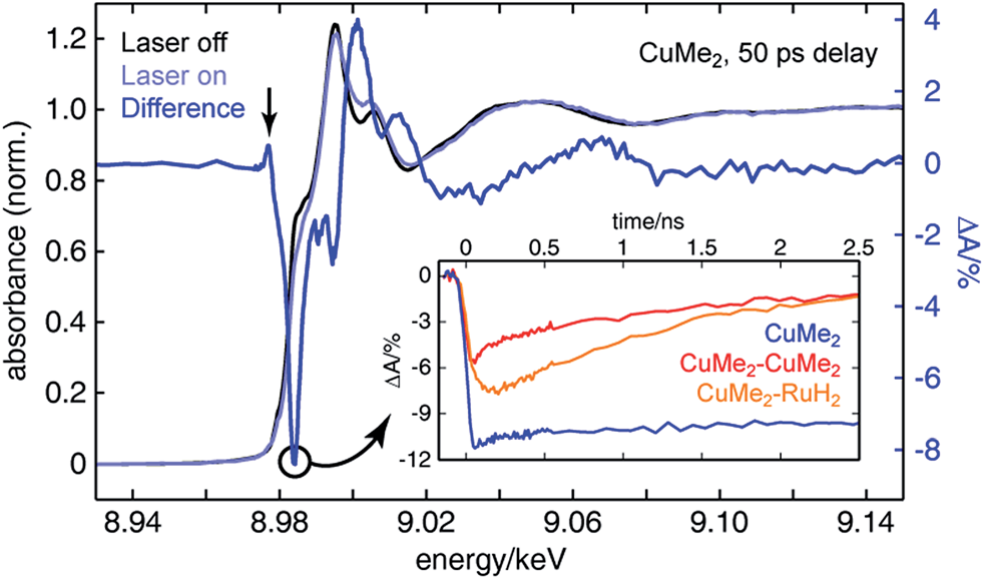
Copper K-edge ground state (black), laser on (light blue), and XTA difference spectrum (dark blue) of **CuMe_2_** 50 ps following optical excitation at 400 nm. The X-ray probe energy used to measure all Cu K-edge XTA kinetic traces is indicated by a circle. (Inset) Copper K-edge XTA kinetic traces of **CuMe_2_** (blue), **CuMe_2_–CuMe_2_** (red), and **CuMe_2_–RuH_2_** (orange). These traces illustrate both the order-of-magnitude longer lifetime of mononuclear **CuMe_2_***vs.* the dinuclear species and the non-impulsive rise time of **CuMe_2_–RuH_2_**.

To assemble a clear picture of the excited state dynamics of the entire family of dinuclear complexes, we acquired a series of XTA spectra at both the copper and ruthenium K-edges for several representative complexes following excitation at 400 nm. All X-ray absorption data was acquired at beamline 11-ID-D of the Advanced Photon Source (APS) at Argonne National Laboratory.^106^–^108^ Because X-ray absorption measurements are element-specific, we are able to monitor the electronic structures of the two metal centers in the heterodinculear complexes individually and thereby cleanly isolate the contributions to the OTA signals from both sides. Fig. 6 shows the ground state X-ray absorption near edge structure (XANES) spectrum of **CuMe_2_** at the Cu K-edge (black) with the “laser on” spectrum (light blue) and corresponding XTA difference spectrum (dark blue) obtained at a delay time of 50 ps overlain. The hallmark features associated with oxidation of the 3d^10^ Cu(i) ground state to the 3d^9^ Cu(ii) ^3^MLCT state are all exceptionally well resolved: the appearance of a pre-edge at 8.977 keV (indicated by a vertical arrow), corresponding to an ESA transition into the laser-induced 3d hole; the bleach of the 1s to 4p peak at 8.984 keV (indicated by a circle), corresponding to the pseudo Jahn–Teller flattening distortion; the hypsochromic shift of the absorption edge, corresponding to stabilization of the 1s orbital; and a phase shift in the extended X-ray absorption fine structure (EXAFS) oscillations, corresponding to a contraction of the Cu–N bond distance. Similar XTA spectra were obtained for **CuH_2_**, **CuH_2_–CuMe_2_**, **CuMe_2_–CuMe_2_**, and **CuMe_2_–RuH_2_** (Fig. S50†).

To follow the evolution of the copper oxidation state following 400 nm excitation into the ^1^MLCT, we acquired XTA time traces at 8.984 keV (the peak of the 1s to 4p bleach in the difference spectra, indicated by a circle in Fig. 6) for **CuMe_2_** (blue), **CuMe_2_–CuMe_2_** (red), and **CuMe_2_–RuH_2_** (orange), which are plotted in the inset of Fig. 6. The temporal resolution of the Cu K-edge XTA measurement (80 ps Gaussian instrument response function, or IRF) does not allow us to follow the initial flattening distortion or ISC events, but we can clearly observe a slow, non-impulsive growth in the Cu(i) depletion of **CuMe_2_–RuH_2_** that is absent in the impulsive **CuMe_2_** and **CuMe_2_–CuMe_2_** traces. As discussed above, we assign this slow growth to intermetallic hole transfer from Ru(iii)* to Cu(i) within the relevant excited state sub-ensemble. Unsurprisingly, the **CuMe_2_** and **CuMe_2_–CuMe_2_** XTA traces appear nearly identical to the corresponding OTA traces, albeit with poorer temporal resolution. The temporal window of the digitally-delayed XTA experiment is much broader, however, which allows us to follow the recovery of the **CuMe_2_** ground state completely and obtain a 32.5 ± 0.5 ns lifetime for the ^3^MLCT (Fig. S52†).

**Fig. 7:**
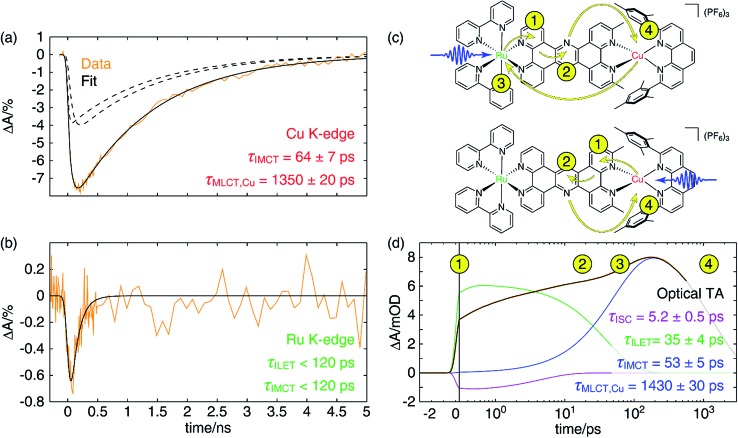
(a) Copper K-edge XTA kinetic trace (orange) of the depletion of the Cu(i) species in **CuMe_2_–RuH_2_** taken at a probe energy of 8.984 keV following 400 nm excitation, and the fit (solid black) to a linear combination of an impulsive and a non-impulsive exponential decay (dashed black). The time constants for the non-impulsive rise (*τ*_IMCT_) and excited state decay (*τ*_MLCT_) are also given. (b) Corresponding ruthenium K-edge XTA kinetic trace (orange) taken at a probe energy of 22.126 keV and the fit (black) to a single exponential decay, corresponding to IMCT. The time constant was found to be shorter than that temporal response of the measurement. (c) Scheme depicting electron transfer pathways in **CuMe_2_–RuH_2_** following optical excitation into the MLCT bands of the Ru(ii) (top) or Cu(i) (bottom) center. Blue arrows represent the excitation pulse, yellow arrows show the movement of electrons through the molecule, and the numbers adjacent to arrowheads indicate the order in which these processes occur. (d) OTA kinetic trace (orange) of **CuMe_2_–RuH_2_** taken at a probe wavelength of 605 nm following excitation at 415 nm and a fit (black) to a three-component model that includes ISC (purple), ILET (green), and IMCT and ground state recovery (blue) processes. The *x*-axis is linearly spaced from –3 to 0.3 ps and logarithmically spaced from 0.3 to 3000 ps; the break is indicated by a solid vertical line. The numbers corresponding to the electron transfer events depicted schematically in panel (c) are also arranged chronologically with the data and fit.

For the heterodinuclear **CuMe_2_–RuH_2_**, we may compare the Cu and Ru K-edge XTA time traces, plotted in Fig. 7, to determine if the data are consistent with the IMCT model described above. Immediately we see that the Ru(ii) depletion associated with formation of the Ru(iii)* MLCT state fully decays within 500 ps (22.126 keV probe energy), suggestive of hole/energy transfer to the Cu(i) site. In contrast, both the OTA and XTA time traces of the homodinuclear species **RuH_2_–RuH_2_** reveal a lifetime of 70 ± 1 ns (Fig. S53†), an order of magnitude shorter than that of the prototypical [Ru(bpy)_3_]^2+^ as expected from the trend described above but at least three orders of magnitude longer than the Ru(iii)* lifetime in the heterodinuclear complex. We note that all Cu K-edge data and the Ru K-edge data for **RuH_2_–RuH_2_** were acquired during standard 24-bunch mode operation of the APS with the aforementioned 80 ps IRF. Meanwhile, the Ru K-edge data for **CuMe_2_–RuH_2_** were acquired during hybrid bunch mode operation, which provides much higher X-ray photon flux but a comparatively long pulse duration, resulting in a 120 ps IRF. The choice to use hybrid mode for measuring the Ru K-edge was made due to the relatively low flux available at the beamline at 22 keV and the low efficiency of the avalanche photodiode fluorescence detectors at such high photon energies. Consequently, because the Ru(iii)* lifetime obtained from a single-component fit of the trace is much less than the experimental resolution, we can only report an upper bound of 120 ps for this time constant from the XTA data. Notably, this measurement is an example of “poor man's beam slicing”, meaning we successfully measured the XTA signal of a transient species with a lifetime shorter than the X-ray pulse duration.

In Fig. 7a, we have plotted a fit (solid black) of the Cu K-edge XTA trace of **CuMe_2_–RuH_2_** (orange) to the sum of two exponential decay terms (dashed black), one with an impulsive rise time and one with a non-impulsive exponential rise time, convolved with a Gaussian instrument response (a detailed description of the fitting models and procedures is given in Section 10 of the ESI†). In this model, the impulsive term corresponds to excitation of the copper MLCT, while the non-impulsive term corresponds to excitation of the ruthenium MLCT followed by hole transfer to the copper site. Notably, even when the lifetimes of the two components are allowed to vary independently, they converge to the same value of 1350 ± 20 ps. This result demonstrates that excitation of either the Cu(i) or Ru(ii) ^1^MLCT ultimately leads to formation of the same final Cu(ii)* ^3^MLCT state from which the complex relaxes to the ground state, confirming our model of simultaneous hole and energy transfer mediated by a common bridging ligand anion as described above. Importantly, the fit gives us a rate of 64 ± 7 ps for IMCT in this particular heterodinuclear system, a value that is indeed within the upper bound obtained from the Ru K-edge data. We also note that the ratio of the amplitudes of the non-impulsive and impulsive fit components is 1.2 to 1, in good agreement with the 1.35 to 1 ratio of the extinction coefficients of the corresponding homodinuclear complexes at 400 nm (see Section 10 of the ESI†), further validating this assignment.

## Discussion

### 1. Charge and energy relays in heterodinuclear complexes

Because of the overlap of the optical absorption spectra of the Cu(i) and Ru(ii) diimine species, it would be challenging to follow the excited state pathways in **CuMe_2_–RuH_2_** using only optical techniques. Armed with the results from the unambiguous element-specific XTA data, however, we may now return to the OTA data to explore the network of overlapping and interconverting ESA signals in greater depth. Fig. 7d shows a fit (black) of the optical time trace of **CuMe_2_–RuH_2_** (orange) taken at a probe wavelength of 605 nm, where both Cu(ii)*- and Ru(iii)*-centered MLCT states absorb (note the data is plotted semi-logarithmically). Because there is no steady state absorption by either the Cu(i) or Ru(ii) at this wavelength, we may exclude from our fitting model any contributions to the OTA signal from GSB response pathways. The simplest model capable of reproducing the data consists of an impulsive ultrafast (few ps) component (purple), an impulsive intermediate (tens of ps) component (green), a non-impulsive slow (few ns) component with an intermediate rise time (blue), and a coherent artifact (FWHM < 1 ps, not pictured). We note that this model affords excellent fits across nearly the entire probe spectrum, and reported time constants are the average of those obtained at all wavelengths across the FWHM of the corresponding TA features (see Section 10 of the ESI† for details).

Because the ultrafast component is negative in sign and there is no ground state absorption at this probe wavelength, we may easily assign this feature to stimulated emission from one or both ^1^MLCT states with a 5.2 ± 0.5 ps ISC time constant. However, because the ISC time constant in [Ru(bpy)_3_]^2+^ is 100 fs or shorter,^109^ it is likely that we cannot capture the ISC dynamics at the ruthenium center within the temporal resolution of our OTA measurement (∼300 fs IRF). Therefore, the 5.2 ps component corresponds exclusively to ISC at the copper center, in agreement with the ISC time constants previously measured for other Cu(i) diimine complexes.^74^ This negative signal is responsible for the apparent non-impulsive rise of the kinetic trace during the first ∼10 ps apparent in Fig. 7d. We may also easily assign the 53 ± 5 ps growth and 1430 ± 30 ps decay of the non-impulsive ESA component to intermetallic charge/energy transfer from Ru(iii)* to Cu(i) and relaxation to the ground state from the Cu(ii)* ^3^MLCT, respectively, based on the good agreement between these two time constants and those obtained from the Cu and Ru K-edge XTA results. Of course, loss of the ESA signal from the Ru(iii)* ^3^MLCT should also occur on the 53 ps timescale, but this decay is simply subsumed into the rise of the non-impulsive component.

Assignment of the impulsive 35 ± 4 ps time constant, on the other hand, is not as immediately obvious. Scandola and coworkers previously reported time constants on the order of tens of ps in related dinuclear compounds and assigned them to intraligand electron transfer (ILET) within **L1**.^50^ Based on extended Hückel calculations,^39^ and the model previously proposed by Flamigni *et al.*,^95^ they concluded that the metal-derived electron is mostly localized within the proximal phenanthroline-like part of **L1** in the initial ^3^MLCT_1_ state but then migrates to the pyrazine-like central ring during relaxation to a lower-lying ^3^MLCT_0_ state. Our calculations support this conclusion as well (see Table S5 and Fig. S49†), and thus we assign the 35 ps time constant to the ILET process. Although there may be some difference in the ILET rates for the Cu(ii)* and Ru(iii)* MLCT states, we find that the data is well modeled with only a single time constant.

The overall electron transfer dynamics/pathways are depicted schematically in Fig. 7c, and the timescales, indicated by numbered circles next to the corresponding arrowheads, are also shown alongside the OTA time trace in Fig. 7d (note the schematic and the following discussion are presented in terms of *electron* transfer, while the previous discussion was presented in terms of hole/energy transfer). Upon excitation at either side, an electron from the metal is immediately transferred to the proximal phenanthroline moiety of **L2**. This is indicated by step 1 and corresponds to the impulsive rise of the ESA signal. Next, ISC results in formation of the ^3^MLCT_1_ state, which relaxes to the ^3^MLCT_0_ state by ILET in 35 ps as indicated by step 2. Shortly thereafter, IMCT occurs in 53 ps as indicated by step 3, but *only* within the sub-ensemble in which the Ru(ii) ^1^MLCT was initially excited (top). Finally, the electron migrates back to the Cu(ii)* in 1.4 ns as the complex relaxes back to the ground state, indicated by step 4.

**Fig. 8:**
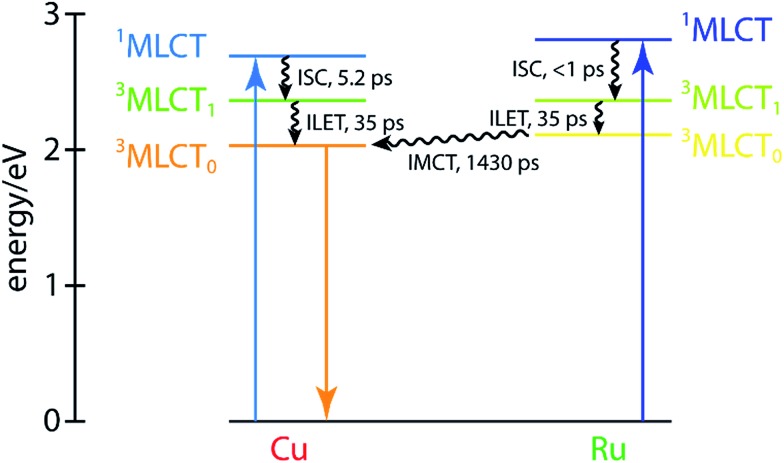
Jablonski diagram for **CuMe_2_–RuH_2_** with the copper- and ruthenium-centered states shown on the left and right sides, respectively. The energies of both ^1^MLCT states and the copper-centered ^3^MLCT_0_ state are estimated from steady-state absorption and emission measurements, and all other energies are qualitative estimates.

These dynamics are also depicted in the Jablonski diagram shown in Fig. 8. The energies of both ^1^MLCT states are estimated from the absorption spectra of the corresponding symmetric dinuclear complexes (Fig. 3), and the copper-centered ^3^MLCT_0_ energy is obtained from the room-temperature emission spectrum of **CuMe_2_–RuH_2_** (Fig. S42†). The energies of the ruthenium-centered ^3^MLCT_1_ or ^3^MLCT_0_ states and the copper-centered ^3^MLCT_0_ state cannot be obtained from the measurements presented here, and thus the driving forces implied by the level spacings are not quantitative.

The OTA data for **CuH_2_–RuH_2_** may also be fit using the same model supplemented by an additional term with a time constant set to be infinite on the 3 ns timescale of the experiment (Fig. S54†). For this complex, we obtain the following time constants: *τ*_ISC/JT_ = 0.6 ± 0.1 ps; *τ*_ILET_ = 2.4 ± 0.2 ps; *τ*_IMCT_ = 21 ± 3 ps; and *τ*_MLCT,Cu_ = 73 ± 1 ps. Because all of the observed time constants are shorter than the IRF of the XTA experiment, however, the assignments in this case are not necessarily unambiguous. For example, the negatively signed, sub-ps component likely includes contributions from both ISC and the pseudo Jahn–Teller distortion, while the value of the lifetime could also be significantly skewed by the presence of a coherent artifact. Furthermore, because both the IMCT and ground state recovery dynamics fall in the range of tens of ps, the network of competing and overlapping processes is not possible to model completely with such a minimal set of fit components. For example, the 52 ps difference between the Cu(ii)* ^3^MLCT lifetime and the IMCT time constant is similar to the 38 ps Cu(ii)* ^3^MLCT lifetime in **CuH_2_–CuH_2_**, suggesting the possibility that the observed lifetime of the copper-based triplet state is extended by the time required for hole/energy transfer from the Ru(iii)* ^3^MLCT to occur. On the other hand, including additional fit components and invoking arguments such as this introduce the risk of overanalyzing the data, so instead we choose to emphasize that these assignments are tentative and made simply in analogy to the temporally distinct dynamics of **CuMe_2_–RuH_2_**. The additional component with *τ* ≫ 3 ns represents only 5% of the total OTA signal and is assigned to minor impurities from mononuclear and/or homodinuclear ruthenium complexes. Although we cannot conclusively verify this origin, the relative magnitude of this component varies significantly between different preparations of **CuH_2_–RuH_2_** while the magnitudes of all other fit components remain constant.

### 2. Charge and energy relays in homodinuclear complexes

The most challenging dynamics to model, of course, are those of the asymmetric homodinuclear copper complex **CuH_2_–CuMe_2_**. For the heterodinuclear complexes, the element specificity of XTA allowed us to spectroscopically separate the transient signals from the two sides and thereby propose, fit, and validate a model of the excited state dynamics. For **CuH_2_–CuMe_2_**, however, both the OTA and XTA spectra of the two coppers overlap nearly completely. While the ground state redox potentials suggest hole/energy transfer should occur from the **CuMe_2_** side to the **CuH_2_** side, the more appropriate values to compare to predict the directionality of transfer would be the excited state redox potentials. Since we cannot measure these values directly, we do not know *a priori* in which direction IMCT should occur in **CuH_2_–CuMe_2_** or even whether it occurs at all. Compounding matters further, the ^3^MLCT_0_ lifetimes obtained for the symmetric homodinuclear complexes (Fig. S51†) are 38 ± 4 ps (**CuH_2_–CuH_2_**) and 1720 ± 50 ps (**CuMe_2_–CuMe_2_**), neither of which may be approximated as impulsive or static on the timescale of the overall dynamics, as with **RuH_2_**. And while even a simple visual comparison of the OTA kinetics traces for **CuMe_2_–RuH_2_**, **CuMe_2_–CuMe_2_**, and **RuH_2_–RuH_2_** clearly demonstrates the occurrence and direction of IMCT in the heterodinuclear complex, the trace of **CuH_2_–CuMe_2_** does not similarly exhibit a lifetime comparable to that of the shorter-lived **CuH_2_–CuH_2_**.

For the above reasons, it is hardly possible to construct an analytical model with which the OTA data of **CuH_2_–CuMe_2_** may be completely and reliably described. Instead, we adopted a semi-empirical approach to inform our evaluation of different models describing the dynamics. Samples of **CuH_2_–CuMe_2_**, **CuH_2_–CuH_2_**, and **CuMe_2_–CuMe_2_** were simultaneously prepared under identical conditions (see Section 12 of the ESI† for details), and OTA spectra were acquired in immediate succession after the entire laser system had stabilized. The dynamics of **CuH_2_–CuH_2_** and **CuMe_2_–CuMe_2_** were then modeled by fitting the kinetic traces at each measured wavelength across the entire probe spectrum (Fig. S51†). Finally, the **CuH_2_–CuMe_2_** kinetics traces were fit to a linear combination of the components used to fit the data of the two symmetric dinculear complexes, using the corresponding average amplitudes and time constants as initial guesses but allowing those parameters to vary.

**Fig. 9:**
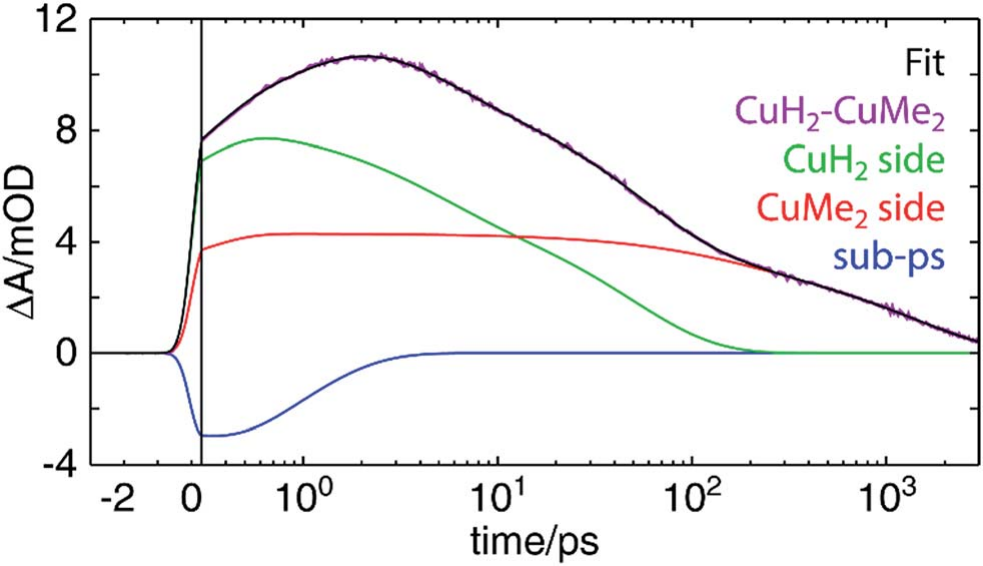
OTA kinetic trace of **CuH_2_–CuMe_2_** at a probe wavelength of 605 nm (purple) and a fit (black) to a linear combination of decay components corresponding to the **CuH_2_** (green) and **CuMe_2_** (red) sides and a sub-ps component (blue). The *x*-axis is linearly spaced from –3 to 0.3 ps and logarithmically spaced from 0.3 to 3000 ps; the break is indicated by a solid vertical line.

Perhaps surprisingly, we see in Fig. 9 that an excellent fit is obtained with time constants very closely matching those found for **CuH_2_–CuH_2_** and **CuMe_2_–CuMe_2_**. These values, as well as those previously discussed for other complexes, are collected in Table 2. For all components but one, the time constants obtained from the **CuH_2_–CuMe_2_** data and the **CuH_2_–CuH_2_** or **CuMe_2_–CuMe_2_** data are the same within experimental error: 0.8 ± 0.2 *vs.* 0.9 ± 0.2 ps for ISC/JT; 4.3 ± 0.4 and 140 ± 30 *vs.* 4.7 ± 0.2 and 120 ± 20 ps for ILET; and 47 ± 6 *vs.* 38 ± 4 ps for Cu(ii)H_2_*^3^MLCT_0_ relaxation. This strongly suggests that the dynamics of the two sides of the asymmetric complex are largely unperturbed from those of their respective symmetric dinuclear analogs, and the assignments made for **CuH_2_–CuH_2_** and **CuMe_2_–CuMe_2_** may also be applied to **CuH_2_–CuMe_2_**. Again, because the sub-ps component is on the order of the IRF, this assignment to ISC/JT is only tentative. The fit components in Fig. 9 combine the ILET and ground state recovery terms for the **CuH_2_** (green) and **CuMe_2_** (red) sides, while the sub-ps component is plotted separately (blue). The same fit with all components plotted separately is shown in Fig. S55.†

The one exception to this trend, however, is the 1460 ± 60 ps time constant, which is of the same magnitude as but notably shorter than the 1720 ± 50 ps ^3^MLCT_0_ lifetime of **CuMe_2_–CuMe_2_**. One possible explanation is that this lifetime simply is shorter in the asymmetric complex, as in the case of **CuMe_2_–RuH_2_**, and the two sides simply do not interact. On the other hand, the shorter lifetime could instead arise from hole/energy transfer from Cu(ii)Me_2_* to **Cu(i)H_2_**. In such a model, if the IMCT time constant were shorter but of the same magnitude as the ^3^MLCT_0_ lifetime, the lifetime obtained from the fit would reflect an intermediate value. This is illustrated numerically in Fig. S57 (see Section 11 of the ESI† for details), where we show that an IMCT time constant of 1286 ps and ground state recovery times corresponding to those found for **CuH_2_–CuH_2_** (38 ps) and **CuMe_2_–CuMe_2_** (1720 ps) would give rise to kinetics that could be fit nearly perfectly with only a 38 ps and a 1460 ps component. Such analysis is not necessary for **CuMe_2_–RuH_2_**, where the IMCT rate is orders of magnitude faster than the Ru(iii)H_2_* MLCT lifetime and thus the overall lifetime is not modulated by relaxation from the Ru(iii)H_2_* state.

A 1286 ps IMCT time constant for this complex is indeed reasonable in comparison to those found for **CuH_2_–RuH_2_** (21 ps) and **CuMe_2_–RuH_2_** (52 ps). The driving force given by the ground state redox potentials, while again not an ideal metric, is much smaller for the asymmetric complex (310 mV) than for the heterodinuclear complexes (790 and 450 mV for **CuH_2_–RuH_2_** and **CuMe_2_–RuH_2_**, respectively). Furthermore, the large reorganization energy associated with the flattening distortion of the oxidized **CuH_2_** side in **CuH_2_–CuMe_2_** would be expected to slow the charge transfer rate relative to that of **CuMe_2_–RuH_2_**.

The model shown in Fig. S57† shows the branching of the Cu(i)H_2_–Cu(ii)Me_2_* population as it relaxes to either the Cu(ii)H_2_*–Cu(i)Me_2_ or **Cu(i)H_2_–Cu(i)Me_2_** state. Because the lifetime of the Cu(ii)H_2_*–Cu(i)Me_2_ state is so short, the population of this intermediate species that arises from IMCT never accumulates above a marginal amount. Nevertheless, because IMCT is slightly faster than relaxation from the Cu(i)H_2_–Cu(ii)Me_2_* state, more than half of the initial excitation on the **CuMe_2_** side migrates to the **CuH_2_** side. From this perspective, the slow IMCT rate effectively acts to lengthen the lifetime of the Cu(ii)H_2_*–Cu(i)Me_2_ state by delaying population of that state. Such behavior could be exploited to engineer molecular systems in which slow photocatalytic processes may be performed using metal centers with otherwise prohibitively short excited state lifetimes. A similar approach using the triplet intraligand state of pyrene as a molecular “battery” in derivatized Ru(ii) bipyridine complexes was first reported by Ford and Rodgers in 1992,^110^ and these systems have since been thoroughly characterized^111^–^113^ and employed in applications ranging from photodynamic therapy^114^,^115^ to photoredox chemistry.^116^

While we cannot conclusively demonstrate that we observe intermetallic hole/energy transfer from the Cu(ii)Me_2_* side to the **CuH_2_** side in **CuH_2_–CuMe_2_** on the timescale of ∼1.3 ps, we can exclude the model in which hole/energy transfer occurs in the opposite direction. In such a case, the additional population of the longer-lived Cu(ii)Me_2_* state resulting from IMCT would skew the relative amplitudes of the long and short decay components toward the long components. Instead, the opposite trend is clear from Fig. 9, where the components associated with the **CuH_2_** side are seen to be substantially greater in amplitude than those of the **CuMe_2_** side. Of course, if IMCT were in fact favorable in this direction but the rate were much slower than the 38 ps lifetime of the Cu(ii)H_2_* state, IMCT would not occur to any significant extent, and the two sides would appear unperturbed.

We may also consider the likelihood of a model in which IMCT does not occur and the Cu(ii)Me_2_* lifetime simply happens to be shorter than in **CuMe_2_–CuMe_2_** by again using a semi-empirical approach. In Fig. S56,† the **CuH_2_–CuMe_2_** kinetic trace at a probe wavelength of 605 nm is fit to a linear combination of the corresponding traces from **CuH_2_–CuH_2_** and **CuMe_2_–CuMe_2_** multiplied by exponential decays to allow the overall lifetimes of the two sides to vary from those of the symmetric complexes. The fit does not satisfactorily reproduce the data from 5 to 30 ps or from 50 to 300 ps, demonstrating that the asymmetric complex cannot be modeled simply as two non-interacting halves. Although processes other than IMCT could be responsible for this disparity, we believe that the model consistent with that of **CuMe_2_–RuH_2_** and **CuH_2_–RuH_2_** is most likely, as internal electrochemical gradients are present in both the asymmetric homodinuclear and heterodinuclear complexes.

Finally, we may not exclude a model in which the hole is delocalized over both coppers and the delocalized MLCT has a uniform ground state recovery time. But given the large metal–metal distance, the electronic coupling between the metal centers is likely small, favoring a localized initial ^1^MLCT excited state. Thus, such a scenario also involves partial hole/energy transfer from the Cu(ii)Me_2_* side to the **Cu(i)H_2_** side (and *vice versa*).

### 3. Novel electronic structural information from ultrafast optical and multi-edge X-ray spectroscopies

The most direct way to obtain a complete description of the excited state dynamics of multimetallic transition metal complexes would be to excite a transition of interest and independently monitor the electronic structure at each metal site. XTA offers exactly this capability for any system bearing only one of each type of metal, while ultrafast optical methods only permit such analysis for exceptionally spectrally distinct compounds. And yet while an extensive body of synthetic and spectroscopic work on heterodinuclear and higher-order transition metal complexes exists, no such multi-edge XTA studies have been reported to our knowledge. We have previously reported a combined metal and ligand K-edge XTA study of photoinduced transient species in hematite thin films,^117^ but the current work is the first XTA study of a molecular species measured at the absorption edges of multiple metals.

Although our ultimate goal was to understand charge and energy transfer dynamics and directionality in an asymmetric homodinuclear Cu(i) complex, we first prepared a heterodinuclear Cu(i)–Ru(ii) analog to allow us to conduct the aforementioned multi-edge XTA analysis. Additionally, the use of a Ru(ii) polypyridine moiety in place of one of the Cu(i) sites greatly simplifies the interpretation of the overall relaxation dynamics of the system, as the Ru(iii)* ^3^MLCT lifetime is 40 times longer than that of the Cu(ii)* state and thus allows us to exclude from our model any contributions from Ru(iii)* relaxation to the ground state. Yet given the prominence of [Ru(bpy)_3_]^2+^ and its derivatives in the photophysical and photochemical literature, there are only a handful of examples of XTA studies at the ruthenium K-edge^107^,^118^–^121^ and L-edge^122^–^124^ that have been reported to date. This dearth of reports is likely due to a combination of the poor efficiency of detectors and the low X-ray flux generally available at time-resolved beamlines at such high photon energies. Fortunately, recent improvements at beamline 11-ID-D at the APS have made such measurements much more feasible. Indeed, in this work we demonstrate the measurement of a Ru(iii)* state with a lifetime only half the duration of the X-ray probe pulse in an example of “poor man's beam slicing” at an energy above 22 keV.

The heterodinuclear multi-edge XTA approach gave us a means of independently monitoring the oxidation states of both the ruthenium and copper with temporal resolution sufficient to unequivocally observe IMCT in **CuMe_2_–RuH_2_**. Tracking this phenomenon in both **CuMe_2_–RuH_2_** and **CuH_2_–RuH_2_** was also simplified by the fact that the timescale for IMCT is orders of magnitude faster than the lifetime of the Ru(iii)H_2_* MLCT state. For the asymmetric homodinuclear **CuH_2_–CuMe_2_**, however, neither OTA nor XTA provides clear, spectrally resolved signals unique to either metal site. Nevertheless, we were able to fit the OTA kinetics of this complex to a model that is consistent with the IMCT model used to describe **CuMe_2_–RuH_2_**, suggesting that we have observed IMCT between spectroscopically overlapping copper sites with differing ligation environments on the nanosecond timescale. This could be further verified through the preparation of additional asymmetric homodinuclear complexes with varying electrochemical gradients by again taking advantage of substitution at the 3,6-tpphz positions.

## Conclusions

An impressive library of Cu(i) bis(phen) complexes exhibiting a broad range of absorption spectra, redox potentials, steric accessibilities, and excited state lifetimes has accumulated over the course of three decades of synthetic efforts toward the goal of creating robust, broadly absorbing earth-abundant photosensitizers and photocatalysts. Incorporating different Cu(i) bis(phen) units into functional assemblies with targeted applications, however, requires a detailed knowledge of the synergistic influence(s) on the overall performance of the assembly that may not be easily predicted solely from the properties of the individual components. Moreover, elucidating the excited state dynamics of asymmetric homodinuclear transition metal complexes represents a considerable challenge, as standard ultrafast spectroscopic tools generally cannot probe individual metal sites independently. We have outlined an experimental strategy for addressing this problem by first synthesizing and characterizing the dynamics of a heterodinuclear analog by multi-edge X-ray transient absorption and traditional ultrafast optical methods. After developing a clear model of the dynamics of this system, we applied this model to the asymmetric homodinuclear case and found evidence that the particular complex explored in this work exhibits directional intermetallic charge transfer in the direction predicted by its ground state electrochemical properties. This behavior suggests a possible design principle for extending the effective lifetimes of otherwise rapidly relaxing chromophores by incorporating them into dinuclear (or multinuclear) assemblies alongside moieties with longer excited state lifetimes. Chromophores with desirable absorption spectra or photocatalytic activity but prohibitively short lifetimes could then be employed without modifying first- or even second-shell coordination geometry, an approach that generally effects steady-state properties. We are currently engaged in continuing experimental and theoretical work to establish an accurate means of predicting the rate and directionality of charge/energy transfer in multimetallic complexes with shared ligands to allow synthetic chemists to take advantage of this behavior. Finally, because the intermetallic dynamics reported here are on the timescale of tens of picoseconds, we believe our strategy could provide even greater insight when executed at X-ray free electron laser sources offering femtosecond resolution.

## Conflicts of interest

There are no conflicts of interest to declare.

## Supplementary Material

Supplementary information

Crystal structure data

## References

[cit1] Lewis N. S., Nocera D. G. (2006). Proc. Natl. Acad. Sci. U. S. A..

[cit2] Morris A. J., Meyer G. J., Fujita E. (2009). Acc. Chem. Res..

[cit3] McDaniel N. D., Bernhard S. (2010). Dalton Trans..

[cit4] Young K. J., Martini L. A., Milot R. L., Snoeberger R. C., Batista V. S., Schmuttenmaer C. A., Crabtree R. H., Brudvig G. W. (2012). Coord. Chem. Rev..

[cit5] Jortner J. (1980). Biochim. Biophys. Acta, Rev. Bioenerg..

[cit6] Breton J., Martin J.-L., Petrich J., Migus A., Antonetti A. (1986). FEBS Lett..

[cit7] Blankenship R. E. (1992). Photosynth. Res..

[cit8] Ort D. R. and Yocum C. F., in Oxygenic Photosynthesis: The Light Reactions, Springer, Dordrecht, 1996, pp. 1–9.

[cit9] Blankenship R. E., Molecular Mechanisms of Photosynthesis, Blackwell Science, Malden, MA, 2002.

[cit10] Holten D., Windsor M. W., Parson W. W., Gouterman M. (1978). Photochem. Photobiol..

[cit11] Meyer T. J. (1989). Acc. Chem. Res..

[cit12] Wasielewski M. R. (1992). Chem. Rev..

[cit13] Sun L., Berglund H., Davydov R., Norrby T., Hammarström L., Korall P., Börje A., Philouze C., Berg K., Tran A., Andersson M., Stenhagen G., Mårtensson J., Almgren M., Styring S., Åkermark B. (1997). J. Am. Chem. Soc..

[cit14] Sun L., Hammarström L., Åkermark B., Styring S. (2001). Chem. Soc. Rev..

[cit15] Gust D., Moore T. A., Moore A. L. (2001). Acc. Chem. Res..

[cit16] Gust D., Moore T. A., Moore A. L. (2009). Acc. Chem. Res..

[cit17] Wasielewski M. R. (2009). Acc. Chem. Res..

[cit18] Magnuson A., Anderlund M., Johansson O., Lindblad P., Lomoth R., Polivka T., Ott S., Stensjö K., Styring S., Sundström V., Hammarström L. (2009). Acc. Chem. Res..

[cit19] Fihri A., Artero V., Razavet M., Baffert C., Leibl W., Fontecave M. (2008). Angew. Chem., Int. Ed..

[cit20] Arachchige S. M., Brown J. R., Chang E., Jain A., Zigler D. F., Rangan K., Brewer K. J. (2009). Inorg. Chem..

[cit21] Wenger O. S. (2009). Coord. Chem. Rev..

[cit22] Mulfort K. L., Tiede D. M. (2010). J. Phys. Chem. B.

[cit23] Concepcion J. J., House R. L., Papanikolas J. M., Meyer T. J. (2012). Proc. Natl. Acad. Sci. U. S. A..

[cit24] Schulz M., Karnahl M., Schwalbe M., Vos J. G. (2012). Coord. Chem. Rev..

[cit25] Vagnini M. T., Smeigh A. L., Blakemore J. D., Eaton S. W., Schley N. D., D'Souza F., Crabtree R. H., Brudvig G. W., Co D. T., Wasielewski M. R. (2012). Proc. Natl. Acad. Sci. U. S. A..

[cit26] Veldkamp B. S., Han W.-S., Dyar S. M., Eaton S. W., Ratner M. A., Wasielewski M. R. (2013). Energy Environ. Sci..

[cit27] Mukherjee A., Kokhan O., Huang J., Niklas J., Chen L. X., Tiede D. M., Mulfort K. L. (2013). Phys. Chem. Chem. Phys..

[cit28] Weingarten A. S., Kazantsev R. V., Palmer L. C., McClendon M., Koltonow A. R., Samuel A. P. S., Kiebala D. J., Wasielewski M. R., Stupp S. I. (2014). Nat. Chem..

[cit29] Bartelmess J., Francis A. J., El Roz K. A., Castellano F. N., Weare W. W., Sommer R. D. (2014). Inorg. Chem..

[cit30] Ashford D. L., Gish M. K., Vannucci A. K., Brennaman M. K., Templeton J. L., Papanikolas J. M., Meyer T. J. (2015). Chem. Rev..

[cit31] Wang L., Mirmohades M., Brown A., Duan L., Li F., Daniel Q., Lomoth R., Sun L., Hammarström L. (2015). Inorg. Chem..

[cit32] Duan L., Wang L., Li F., Li F., Sun L. (2015). Acc. Chem. Res..

[cit33] Mulfort K. L., Utschig L. M. (2016). Acc. Chem. Res..

[cit34] Favereau L., Makhal A., Pellegrin Y., Blart E., Petersson J., Göransson E., Hammarström L., Odobel F. (2016). J. Am. Chem. Soc..

[cit35] Orazietti M., Kuss-Petermann M., Hamm P., Wenger O. S. (2016). Angew. Chem., Int. Ed..

[cit36] Mulfort K. L. (2017). C. R. Chim..

[cit37] Knapp R., Schott A., Rehahn M. (1996). Macromolecules.

[cit38] Bolger J., Gourdon A., Ishow E., Launay J.-P. (1995). J. Chem. Soc., Chem. Commun..

[cit39] Bolger J., Gourdon A., Ishow E., Launay J.-P. (1996). Inorg. Chem..

[cit40] Bodige S., Torres A. S., Maloney D. J., Tate D., Kinsel G. R., Walker A. K., MacDonnell F. M. (1997). J. Am. Chem. Soc..

[cit41] Ishow E., Gourdon A., Launay J.-P., Lecante P., Verelst M., Chiorboli C., Scandola F., Bignozzi C.-A. (1998). Inorg. Chem..

[cit42] Campagna S., Serroni S., Bodige S., MacDonnell F. M. (1999). Inorg. Chem..

[cit43] Kim M.-J., MacDonnell F. M., Gimon-Kinsel M. E., Du Bois T., Asgharian N., Griener J. C. (2000). Angew. Chem., Int. Ed..

[cit44] MacDonnell F. M., Bodige S. (1996). Inorg. Chem..

[cit45] Komatsuzaki N., Katoh R., Himeda Y., Sugihara H., Arakawa H., Kasuga K. (2000). J. Chem. Soc., Dalton Trans..

[cit46] Frey J., Kraus T., Heitz V., Sauvage J.-P. (2007). Chem.–Eur. J..

[cit47] Chiorboli C., Bignozzi C. A., Scandola F., Ishow E., Gourdon A., Launay J.-P. (1999). Inorg. Chem..

[cit48] Tysoe S. A., Kopelman R., Schelzig D. (1999). Inorg. Chem..

[cit49] Torieda H., Nozaki K., Yoshimura A., Ohno T. (2004). J. Phys. Chem. A.

[cit50] Chiorboli C., Rodgers M. A. J., Scandola F. (2003). J. Am. Chem. Soc..

[cit51] Canton S. E., Zhang X., Zhang J., van Driel T. B., Kjaer K. S., Haldrup K., Chabera P., Harlang T., Suarez-Alcantara K., Liu Y., Pérez J., Bordage A., Pápai M., Vankó G., Jennings G., Kurtz C. A., Rovezzi M., Glatzel P., Smolentsev G., Uhlig J., Dohn A. O., Christensen M., Galler A., Gawelda W., Bressler C., Lemke H. T., Møller K. B., Nielsen M. M., Lomoth R., Wärnmark K., Sundström V. (2013). J. Phys. Chem. Lett..

[cit52] Pfeffer M. G., Pehlken C., Staehle R., Sorsche D., Streb C., Rau S. (2014). Dalton Trans..

[cit53] Sorsche D., Schaub M., Heinemann F. W., Habermehl J., Kuhri S., Guldi D., Guthmuller J., Rau S. (2016). Dalton Trans..

[cit54] Schmittel M., Ganz A. (1997). Chem. Commun..

[cit55] Schmittel M., Lüning U., Meder M., Ganz A., Michel C., Herderich M. (1997). Heterocycl. Commun..

[cit56] Miller M. T., Gantzel P. K., Karpishin T. B. (1999). J. Am. Chem. Soc..

[cit57] Champin B., Mobian P., Sauvage J.-P. (2007). Chem. Soc. Rev..

[cit58] Listorti A., Accorsi G., Rio Y., Armaroli N., Moudam O., Gégout A., Delavaux-Nicot B., Holler M., Nierengarten J.-F. (2008). Inorg. Chem..

[cit59] De S., Mahata K., Schmittel M. (2010). Chem. Soc. Rev..

[cit60] Kabehie S., Xue M., Stieg A. Z., Liong M., Wang K. L., Zink J. I. (2010). J. Am. Chem. Soc..

[cit61] Pellegrin Y., Sandroni M., Blart E., Planchat A., Evain M., Bera N. C., Kayanuma M., Sliwa M., Rebarz M., Poizat O., Daniel C., Odobel F. (2011). Inorg. Chem..

[cit62] Kayanuma M., Bera N., Sandroni M., Pellegrin Y., Blart E., Odobel F., Daniel C. (2012). C. R. Chim..

[cit63] Lazorski M. S., Gest R. H., Elliott C. M. (2012). J. Am. Chem. Soc..

[cit64] Sandroni M., Kayanuma M., Planchat A., Szuwarski N., Blart E., Pellegrin Y., Daniel C., Boujtita M., Odobel F. (2013). Dalton Trans..

[cit65] Sandroni M., Kayanuma M., Rebarz M., Akdas-Kilig H., Pellegrin Y., Blart E., Bozec H. L., Daniel C., Odobel F. (2013). Dalton Trans..

[cit66] Fraser M. G., van der Salm H., Cameron S. A., Blackman A. G., Gordon K. C. (2013). Inorg. Chem..

[cit67] Mohankumar M., Monti F., Holler M., Niess F., Delavaux-Nicot B., Armaroli N., Sauvage J.-P., Nierengarten J.-F. (2014). Chem.–Eur. J..

[cit68] Sandroni M., Maufroy A., Rebarz M., Pellegrin Y., Blart E., Ruckebusch C., Poizat O., Sliwa M., Odobel F. (2014). J. Phys. Chem. C.

[cit69] Sandroni M., Pellegrin Y., Odobel F. (2016). C. R. Chim..

[cit70] Kohler L., Hayes D., Hong J., Carter T. J., Shelby M. L., Fransted K. A., Chen L. X., Mulfort K. L. (2016). Dalton Trans..

[cit71] Kohler L., Hadt R. G., Hayes D., Chen L. X., Mulfort K. L. (2017). Dalton Trans..

[cit72] Blaskie M. W., McMillin D. R. (1980). Inorg. Chem..

[cit73] Ruthkosky M., Kelly C. A., Castellano F. N., Meyer G. J. (1998). Coord. Chem. Rev..

[cit74] Mara M. W., Fransted K. A., Chen L. X. (2015). Coord. Chem. Rev..

[cit75] Iwamura M., Takeuchi S., Tahara T. (2015). Acc. Chem. Res..

[cit76] Cunningham C. T., Cunningham K. L. H., Michalec J. F., McMillin D. R. (1999). Inorg. Chem..

[cit77] Chen L. X., Shaw G. B., Novozhilova I., Liu T., Jennings G., Attenkofer K., Meyer G. J., Coppens P. (2003). J. Am. Chem. Soc..

[cit78] Chen L. X. (2003). Faraday Discuss..

[cit79] Siddique Z. A., Yamamoto Y., Ohno T., Nozaki K. (2003). Inorg. Chem..

[cit80] Iwamura M., Takeuchi S., Tahara T. (2007). J. Am. Chem. Soc..

[cit81] Armaroli N., Accorsi G., Cardinali F. and Listorti A., in Photochemistry and Photophysics of Coordination Compounds I, Springer, Berlin, Heidelberg, 2007, pp. 69–115.

[cit82] Shaw G. B., Grant C. D., Shirota H., Castner E. W., Meyer G. J., Chen L. X. (2007). J. Am. Chem. Soc..

[cit83] Iwamura M., Watanabe H., Ishii K., Takeuchi S., Tahara T. (2011). J. Am. Chem. Soc..

[cit84] Huang J., Buyukcakir O., Mara M. W., Coskun A., Dimitrijevic N. M., Barin G., Kokhan O., Stickrath A. B., Ruppert R., Tiede D. M., Stoddart J. F., Sauvage J.-P., Chen L. X. (2012). Angew. Chem., Int. Ed..

[cit85] Tromp M., Dent A. J., Headspith J., Easun T. L., Sun X.-Z., George M. W., Mathon O., Smolentsev G., Hamilton M. L., Evans J. (2013). J. Phys. Chem. B.

[cit86] Penfold T. J., Karlsson S., Capano G., Lima F. A., Rittmann J., Reinhard M., Rittmann-Frank M. H., Braem O., Baranoff E., Abela R., Tavernelli I., Rothlisberger U., Milne C. J., Chergui M. (2013). J. Phys. Chem. A.

[cit87] McCusker C. E., Castellano F. N. (2013). Inorg. Chem..

[cit88] Mara M. W., Jackson N. E., Huang J., Stickrath A. B., Zhang X., Gothard N. A., Ratner M. A., Chen L. X. (2013). J. Phys. Chem. B.

[cit89] Bozic-Weber B., Constable E. C., Fürer S. O., Housecroft C. E., Troxler L. J., Zampese J. A. (2013). Chem. Commun..

[cit90] Mara M. W., Bowman D. N., Buyukcakir O., Shelby M. L., Haldrup K., Huang J., Harpham M. R., Stickrath A. B., Zhang X., Stoddart J. F., Coskun A., Jakubikova E., Chen L. X. (2015). J. Am. Chem. Soc..

[cit91] Kelley M. S., Shelby M. L., Mara M. W., Haldrup K., Hayes D., Hadt R. G., Zhang X., Stickrath A. B., Ruppert R., Sauvage J.-P., Zhu D., Lemke H. T., Chollet M., Schatz G. C., Chen L. X. (2017). J. Phys. B: At., Mol. Opt. Phys..

[cit92] Ferraudi G., Muralidharan S. (1981). Coord. Chem. Rev..

[cit93] Kutal C. (1990). Coord. Chem. Rev..

[cit94] Horváth O. (1994). Coord. Chem. Rev..

[cit95] Flamigni L., Encinas S., Barigelletti F., MacDonnell F. M., Kim K.-J., Puntoriero F., Campagna S. (2000). Chem. Commun..

[cit96] Scandola F., Bignozzi C. A., Chiorboli C., Indelli M. T., Rampi M. A. (1990). Coord. Chem. Rev..

[cit97] Bignozzi C. A., Argazzi R., Chiorboli C., Roffia S., Scandola F. (1991). Coord. Chem. Rev..

[cit98] Serroni S., Campagna S., Denti G., Keyes T. E., Vos J. G. (1996). Inorg. Chem..

[cit99] Argazzi R., Bertolasi E., Chiorboli C., Bignozzi C. A., Itokazu M. K., Murakami Iha N. Y. (2001). Inorg. Chem..

[cit100] Canton S. E., Kjær K. S., Vankó G., van Driel T. B., Adachi S., Bordage A., Bressler C., Chabera P., Christensen M., Dohn A. O., Galler A., Gawelda W., Gosztola D., Haldrup K., Harlang T., Liu Y., Møller K. B., Németh Z., Nozawa S., Pápai M., Sato T., Sato T., Suarez-Alcantara K., Togashi T., Tono K., Uhlig J., Vithanage D. A., Wärnmark K., Yabashi M., Zhang J., Sundström V., Nielsen M. M. (2015). Nat. Commun..

[cit101] Chen L. X., in X-Ray Absorption and X-Ray Emission Spectroscopy, ed. J. A. V. Bokhoven and C. Lamberti, John Wiley & Sons, Ltd, Hoboken, NJ, 2016, pp. 213–249.

[cit102] Huang W., Ogawa T. (2006). Polyhedron.

[cit103] Finck S., Issenhuth J.-T., Despax S., Sirlin C., Pfeffer M., Poidevin C., Gourlaouen C., Boeglin A., Daniel C. (2014). J. Organomet. Chem..

[cit104] Chen L. X., Jennings G., Liu T., Gosztola D. J., Hessler J. P., Scaltrito D. V., Meyer G. J. (2002). J. Am. Chem. Soc..

[cit105] Gordon K. C., McGarvey J. J. (1991). Inorg. Chem..

[cit106] Jennings G., Jäger W. J. H., Chen L. X. (2002). Rev. Sci. Instrum..

[cit107] Zhang X., Smolentsev G., Guo J., Attenkofer K., Kurtz C., Jennings G., Lockard J. V., Stickrath A. B., Chen L. X. (2011). J. Phys. Chem. Lett..

[cit108] Chen L. X., Zhang X. (2013). J. Phys. Chem. Lett..

[cit109] Damrauer N. H., Cerullo G., Yeh A., Boussie T. R., Shank C. V., McCusker J. K. (1997). Science.

[cit110] Ford W. E., Rodgers M. A. J. (1992). J. Phys. Chem..

[cit111] Simon J. A., Curry S. L., Schmehl R. H., Schatz T. R., Piotrowiak P., Jin X., Thummel R. P. (1997). J. Am. Chem. Soc..

[cit112] Kozlov D. V., Tyson D. S., Goze C., Ziessel R., Castellano F. N. (2004). Inorg. Chem..

[cit113] McClenaghan N. D., Leydet Y., Maubert B., Indelli M. T., Campagna S. (2005). Coord. Chem. Rev..

[cit114] Monro S., Scott J., Chouai A., Lincoln R., Zong R., Thummel R. P., McFarland S. A. (2010). Inorg. Chem..

[cit115] Lincoln R., Kohler L., Monro S., Yin H., Stephenson M., Zong R., Chouai A., Dorsey C., Hennigar R., Thummel R. P., McFarland S. A. (2013). J. Am. Chem. Soc..

[cit116] Guerzo D., Leroy S., Fages F., Schmehl R. H. (2002). Inorg. Chem..

[cit117] Hayes D., Hadt R. G., Emery J. D., Cordones A. A., Martinson A. B. F., Shelby M. L., Fransted K. A., Dahlberg P. D., Hong J., Zhang X., Kong Q., Schoenlein R. W., Chen L. X. (2016). Energy Environ. Sci..

[cit118] Harpham M. R., Nguyen S. C., Hou Z., Grossman J. C., Harris C. B., Mara M. W., Stickrath A. B., Kanai Y., Kolpak A. M., Lee D., Liu D.-J., Lomont J. P., Moth-Poulsen K., Vinokurov N., Chen L. X., Vollhardt K. P. C. (2012). Angew. Chem., Int. Ed..

[cit119] Sato T., Nozawa S., Tomita A., Hoshino M., Koshihara S., Fujii H., Adachi S. (2012). J. Phys. Chem. C.

[cit120] Harpham M. R., Stickrath A. B., Zhang X., Huang J., Mara M. W., Chen L. X., Liu D.-J. (2013). J. Phys. Chem. A.

[cit121] Borfecchia E., Garino C., Salassa L., Ruiu T., Gianolio D., Zhang X., Attenkofer K., Chen L. X., Gobetto R., Sadler P. J., Lamberti C. (2013). Dalton Trans..

[cit122] Saes M., Bressler C., Abela R., Grolimund D., Johnson S. L., Heimann P. A., Chergui M. (2003). Phys. Rev. Lett..

[cit123] Gawelda W., Johnson M., de Groot F. M. F., Abela R., Bressler C., Chergui M. (2006). J. Am. Chem. Soc..

[cit124] Van Kuiken B. E., Huse N., Cho H., Strader M. L., Lynch M. S., Schoenlein R. W., Khalil M. (2012). J. Phys. Chem. Lett..

