# Supplementary material for: Excited state electron and energy relays in supramolecular dinuclear complexes revealed by ultrafast optical and X-ray transient absorption spectroscopy
Source: Chem Sci. 2017 Nov 28;9(4):860–75. doi: 10.1039/c7sc04055e (PMC5873173; doi:10.1039/c7sc04055e)
Supplement: Supplementary file 1 [file SC-009-C7SC04055E-s001.pdf]

Electronic Supplementary Material (ESI) for Chemical Science.
This journal is © The Royal Society of Chemistry 2017

Electronic Supplementary Information

Excited state electron and energy relays in supramolecular dinuclear complexes revealed by ultrafast optical and X-ray transient absorption spectroscopy

Dugan Hayes,*‡[a] Lars Kohler,§[a] Ryan G. Hadt,§[a] Xiaoyi Zhang,[b] Cunming Liu,[b] Karen L. Mulfort,*[a] and Lin X. Chen*[ac]

[a]Chemical Sciences and Engineering Division, Argonne National Laboratory, Argonne, IL 60439, USA. E-mail: dugan@uri.edu, mulfort@anl.gov, lchen@anl.gov
[b]X-ray Science Division, Argonne National Laboratory, Argonne, IL 60439, USA
[c]Department of Chemistry, Northwestern University, Evanston, IL 60208, USA
‡ Current address: Department of Chemistry, University of Rhode Island, Kingston, Rhode Island 02881.
§ These authors contributed equally.

1. Synthesis schemes (Schemes 1-3; pp. 2-3)
2. Synthetic experimental details (pp. 4-8)
3. NMR spectra (Figures S1-S41; pp. 9-29)
4. Single crystal X-ray diffraction (Tables S1-S2; pp. 30-31)
5. Steady-state spectroscopic characterization (Figure S42; pp. 32)
6. Cyclic voltammetry (Figures S43-S44; pp. 33-34)
7. Electronic structure calculations (Tables S3-S6, Figures S45-S49; pp. 35-43)
8. Femtosecond and nanosecond optical transient absorption (p. 44)
9. Copper and ruthenium K-edge absorption (Figure S50; pp. 45-46)
10. Data analysis and fitting (Figures S51-S56, Equations S1-S4; pp. 47-50)
11. Numerical modeling of IMCT (Figure S57, Equations S5-S7; pp. 51-52)
12. Sample preparation for semi-empirical OTA modeling (Figure S58; p. 53)
13. References (p. 54)

1. Synthesis schemes

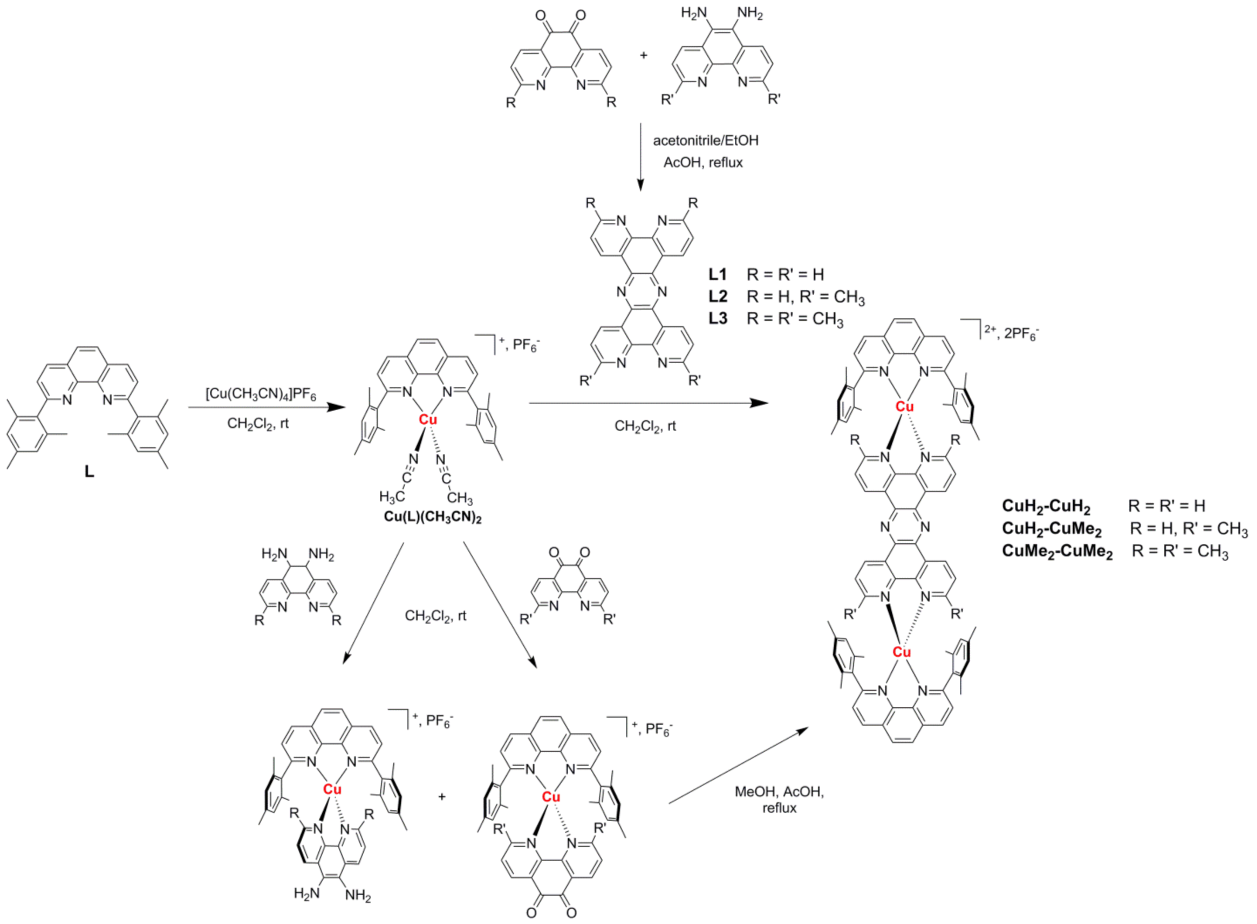

Scheme S1. Synthesis of bridging ligands **L1**, **L2**, and **L3** and dinuclear Cu(I)-Cu(I) complexes.

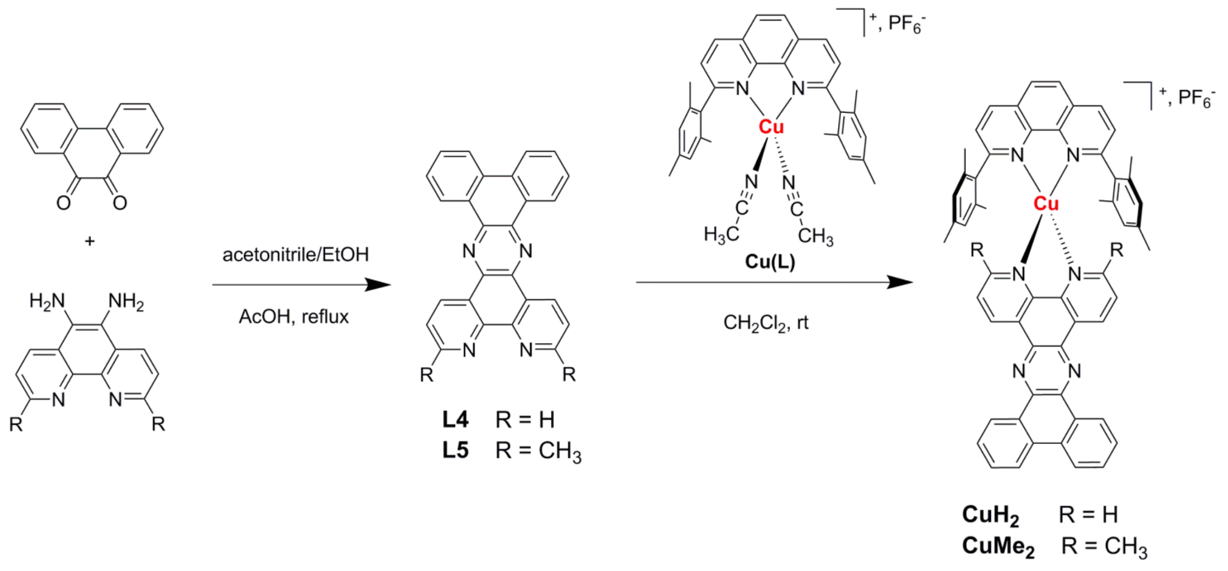

Scheme S2. Synthesis of mononuclear Cu(I) complexes.

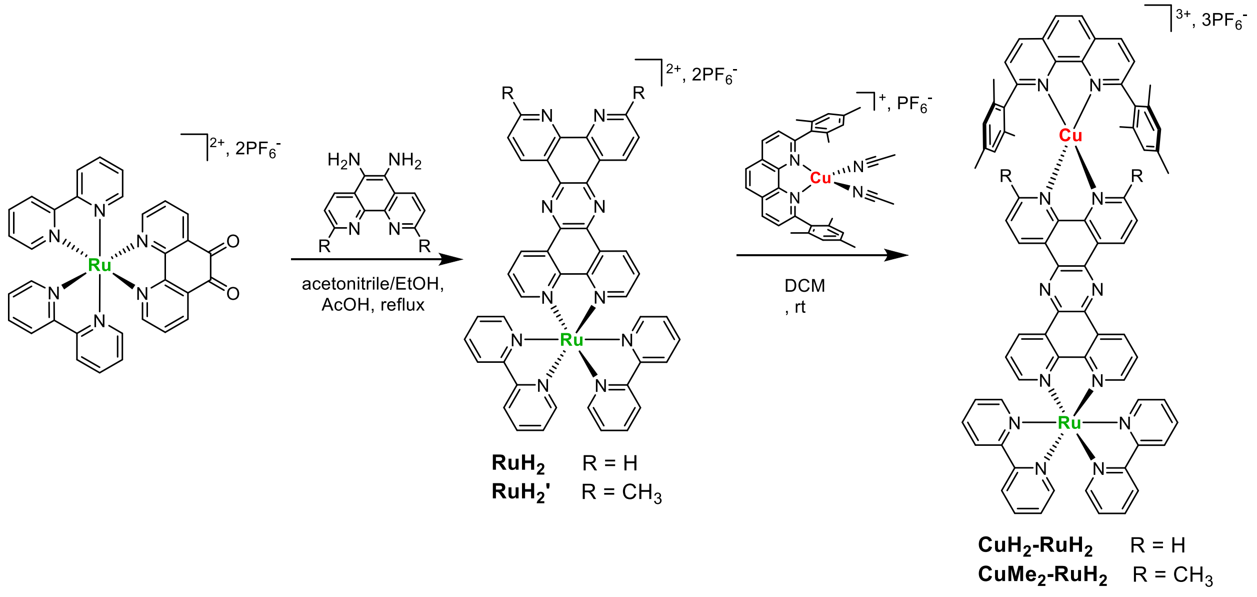

Scheme S3. Synthesis of dinuclear Cu(I)-Ru(II) complexes.

2. Synthetic experimental details

General considerations. All reagents and solvents were purchased from commercial sources and were used as received. Several ligands were prepared according to published procedures: 2,9-dimethyl-1,10-phenanthroline-5,6-dione;[1] 6-nitro-1,10-phenanthroline-5-amine, **L1** (tetrapyrido[3,2-*a*:2',3'-*c*:3'',2''-*h*:2''',3'''-*j*]phenazine), **RuH$_2$**, and **RuH$_2$-RuH$_2$**;[2] 2,9-dimethyl-6-nitro-1,10-phenanthroline-5-amine diamine and 2,9-dimethyl-1,10-phenanthroline-5,6-diamine;[3] **L** (2,9-dimesityl-1,10-phenanthroline);[4,5] and **L4** (dibenzo-[*a*,*c*]dipyrido[3,2-*h*:2',3'-*j*]phenazine).[6] ^{1}H NMR was performed on a Bruker DMX 500 and referenced to TMS or residual solvent peak. ESI-MS was collected on a ThermoFisher LCQ Fleet from dilute CH_3CN or methanol solutions in positive ionization mode. Elemental analysis was performed by Midwest Microlab, LLC (Indianapolis, IN, USA).

L2 (3,6-dimethyltetrapyrido[3,2-*a*:2',3'-*c*:3'',2''-*h*:2''',3'''-*j*]phenazine): 2,9-Dimethyl-1,10-phenanthroline-5,6-diamine (100 mg, 0.42 mmol) and 1,10-phenanthroline-5,6-dione (88.2 mg, 0.42 mmol) were suspended in an acetonitrile/ethanol mixture (2:1, 30 mL) and refluxed for 16 hours. The reaction mixture was allowed to cool to room temperature, the precipitate was filtered and washed with acetonitrile. The product was isolated as an off-white powder in 59% yield (102 mg). ^{1}H NMR ($CDCl_3$): δ 9.60 (2H, dd, J = 8.1 Hz, J = 1.7 Hz); 9.51 (2H, d, J = 8.2 Hz); 9.31 (2H, dd, J = 4.3 Hz, J = 1.8); 7.80 (2H, dd, J = 8.1 Hz, J = 4.4 Hz); 7.68 (2H, d, J = 8.3 Hz); 3.06 (6H, s). ^{13}C NMR ($CDCl_3$): δ 162.3; 152.3; 147.7; 147.2; 139.5; 139.1; 133.4; 133.0; 126.8; 124.6; 124.3; 123.9; 26.0. Anal. calcd. for **L2**, $C_{26}H_{16}N_6{\cdot}1.25H_2O$: C, 71.79; H, 4.29; N, 19.32. Found: C, 71.54; H, 4.02; N, 19.09.

L3 (3,6,12,15-tetramethyltetrapyrido[3,2-*a*:2',3'-*c*:3'',2''-*h*:2''',3'''-*j*]phenazine): 2,9-Dimethyl-1,10-phenanthroline-5,6-diamine (60.0 mg, 0.25 mmol) and 2,9-dimethyl-1,10-phenanthroline-5,6-dione (60.0 mg, 0.25 mmol) were suspended in an acetonitrile/ethanol mixture (2:1, 15 mL) and refluxed for 16 hours. The reaction mixture was concentrated and the solid residue was suspended in acetone. The solid was isolated by filtration, washed with acetone and a small amount of acetonitrile to give the product as an off-white powder in 78% yield (87 mg). ^{1}H NMR ($CDCl_3$): δ 9.64 (4H, d, J = 8.0 Hz); 7.72 (4H, d, J = 8.0 Hz); 3.05 (12H, s). ^{13}C NMR ($CDCl_3$): δ 162.0; 147.2; 139.3; 133.4; 124.9; 124.3; 26.0. Anal. calcd. for **L3**, $C_{28}H_{20}N_6{\cdot}2H_2O$: C, 70.57; H, 5.08; N, 17.64. Found: C, 70.68; H, 4.78; N, 17.36.

L5 (3,6-dimethyldibenzo[*a*,*c*]dipyrido[3,2-*h*:2',3'-*j*]phenazine): 2,9-Dimethyl-1,10-phenanthroline-5,6-diamine (100 mg, 0.42 mmol) and 9,10-phenanthrenequinone (87.4 mg, 0.42 mmol) were suspended in an acetonitrile/ethanol mixture (2:1, 15 mL) and refluxed for 16 hours. The reaction mixture was allowed to cool to room temperature, and the precipitate was filtered and washed with acetonitrile, ethanol and dichloromethane. The product was isolated as a yellow powder in 99% yield (170 mg). ^{1}H NMR ($(CD_3)_2SO$): δ 9.62 (2H, d, J = 7.3 Hz); 9.49 (2H, d, J = 7.6 Hz); 8.88 (2H, d, J = 7.9 Hz); 7.94 (2H, t, J = 7.1 Hz); 7.89 (2H, t, J = 7.2 Hz); 7.83 (2H, d, J = 8.0 Hz); 2.88 (6H, s). Anal. calcd. for **L5**, $C_{28}H_{18}N_4{\cdot}0.75CH_2Cl_2$: C, 72.82; H, 4.15; N, 11.82. Found: C, 72.75; H, 4.26; N, 11.93.

CuH$_2$: $[Cu(CH_3CN)_4]PF_6$ (68.2 mg, 0.18 mmol) was added to a round bottom flask and dissolved in dichloromethane (20 ml) with stirring. The clear colorless solution was deaerated with N_2. A similarly deaerated solution of **L** (76.2 mg, 0.18 mmol) in dichloromethane (10 ml) was added to the reaction mixture. Upon addition of **L**, the solution turned bright yellow, and was allowed to stir at room temperature for five minutes. **L4** (70.0 mg, 0.18 mmol) was then added followed by dichloromethane (20 mL). The reaction mixture stayed cloudy after 2 hours stirring at room temperature. Acetonitrile (2 mL)

was added and the resulting clear red solution was stirred under N_2 at room temperature for two hours. The mixture was filtered and concentrated by rotary evaporation and the product was precipitated with diethyl ether. The orange solid was isolated by filtration and allowed to dry in air to give complex **CuH₂** (184 mg, 99% yield). ^1H NMR ($(CD_3)CO$): δ 9.64 (2H, d, J = 8.2 Hz), 9.47 (2H, d, J = 7.9 Hz), 9.06 (2H, dd, J = 4.7 Hz, J = 1.4Hz), 9.00 (2H, d, J = 8.2 Hz), 8.45 (2H, s), 8.06-8.12 (4H, m), 8.00 (2H, d, J = 8.2 Hz), 7.83 (2H, t, J = 7.3 Hz), 7.66 (2H, t, J = 6.9 Hz), 6.21 (4H, s), 1.88 (12H, s), 1.30 (6H, s). Anal. calcd. for **CuH_2**, $C_{56}H_{42}CuF_6N_6P·H_2O$: C, 65.59; H, 4.32; N, 8.20. Found: C, 65.41; H, 4.28; N, 8.21.

$CuMe_2$: $[Cu(CH_3CN)_4]PF_6$ (63.5 mg, 0.17 mmol) was added to a round bottom flask and dissolved in dichloromethane (20 mL) with stirring. The clear colorless solution was deaerated with N_2. A similarly deaerated solution of **L** (71.0 mg, 0.17 mmol) in dichloromethane (10 mL) was added to the reaction mixture. Upon addition of **L**, the solution turned bright yellow, and was allowed to stir at room temperature for five minutes. **L5** (70.0 mg, 0.17 mmol) was then added followed by dichloromethane (20 mL). The resulting red solution was stirred under N_2 at room temperature for three hours. The mixture was filtered and concentrated by rotary evaporation and the product was precipitated with diethyl ether. The orange solid was isolated by filtration and allowed to dry in air to give complex **$CuMe_2$** (166 mg, 94% yield). ^1H NMR ($(CD_3)CO$): δ 9.58 (2H, d, J = 8.2 Hz), 9.49 (2H, d, J = 7.5 Hz), 9.03 (2H, d, J = 8.2 Hz), 8.47 (2H, s), 8.31 (2H, d, J = 7.9 Hz), 8.03 (2H, d, J = 8.2 Hz), 7.93 (2H, d, J = 8.3 Hz), 7.83 (2H, t, J = 7.2 Hz), 7.69 (2H, t, J = 7.6 Hz), 6.27 (4H, s), 2.52 (6H, s), 1.81 (12H, s), 1.49 (6H, s). Anal. calcd. for **$CuMe_2$**, $C_{58}H_{46}CuF_6N_6P·H_2O$: C, 66.12; H, 4.59; N, 7.98. Found: C, 65.99; H, 4.37; N, 7.79.

[Cu(L)(2,9-dimethyl-1,10-phenanthroline-5,6-dione)](PF_6): The same procedure as described for **$CuMe_2$** was followed using $[Cu(CH_3CN)_4]PF_6$ (305.2 mg, 0.82 mmol), 2,9-dimesityl-1,10-phenanthroline (341.1 mg, 0.82 mmol) and 2,9-dimethyl-1,10-phenanthrolin-5,6-dione (195 mg, 0.82 mmol) to afford an red solid (689 mg, 97% yield). ^1H NMR ($(CD_3)_2CO$): δ 9.01 (2H, d, J = 8.0 Hz); 8.49 (2H, d, J = 8.0 Hz); 8.44 (2H, s); 8.07 (2H, d, J = 8.0 Hz); 7.75 (2H, d, J = 8.0 Hz); 6.51 (4H, s); 2.27 (6H, s); 1.93 (6H, s); 1.76 (12H, s). ESI-MS (CH_3CN): calcd. $[M-PF_6^-]^+$ 717.23; obsd. 717.50. Anal. calcd. for [Cu(**L**)(2,9-dimethyl-1,10-phenanthroline-5,6-dione)](PF_6), $C_{44}H_{38}CuF_6N_4O_2P·1/3CH_2Cl_2$: C, 59.72; H, 4.37; N, 6.28. Found: C, 59.72; H, 4.39; N, 6.20.

[Cu(L)(1,10-phenanthrolin-5,6-diamine)](PF_6): $[Cu(CH_3CN)_4]PF_6$ (400 mg, 1.07 mmol) was added to a round bottom flask and dissolved in dichloromethane (50 mL) with stirring. The clear colorless solution was deaerated with N_2. A similarly deaerated solution of **L** (447.1 mg, 1.07 mmol) in dichloromethane (20 mL) was added to the reaction mixture. Upon addition of **L**, the solution turned bright yellow, and was allowed to stir at room temperature for five minutes. 6-Nitro-1,10-phenanthrolin-5-amine (257.8 mg, 1.07 mmol) was then added followed by dichloromethane (20 mL). The resulting red solution was stirred under N_2 at room temperature for three hours. The mixture was filtered and concentrated by rotary evaporation and the product was precipitated with diethyl ether. The orange solid was isolated by filtration and allowed to dry in air to give complex [Cu(**L**)(6-Nitro-1,10-phenanthrolin-5-amine)](PF_6) (869 mg, 94% yield). ^1H NMR ($CDCl_3$): δ 9.10 (1H, d, J = 4.0 Hz); 9.08 (1H, d, J = 3.5 Hz); 8.63 (2H, d, J = 8.3 Hz); 8.47 (1H, d, J =4.6 Hz); 8.12-8.20 (4H, m); 7.88 (1H, dd, J = 8.2 Hz, J = 4.6 Hz); 7.81 (2H, d, J = 8.2 Hz); 7.57 (1H, dd, J = 8.4 Hz, J = 4.6 Hz); 6.21 (2H, s); 6.15 (2H, s); 1.80 (6H, s); 1.71 (12H, s). ESI-MS (CH_3OH): calcd. $[M-PF_6^-]^+$ 719.22; obsd. 719.58. [Cu(**L**)(6-Nitro-1,10-phenanthrolin-5-amine)](PF_6) was used without further purification for the next synthetic step. [Cu(**L**)(6-Nitro-1,10-phenanthrolin-5-amine)](PF_6) (400 mg, 0.46 mmol) was suspended in a methanol/ethanol mixture (1:1; 200 ml). The mixture was heated to reflux while deaerated with N_2 and palladium on carbon (10%; 400 mg) was added. Hydrazine (1 ml) dissolved

in ethanol (10 ml) was added dropwise over a 30 min period and the reaction was refluxed for 1 hour. After hot filtration through Celite the dark red solution was concentrated and the product precipitated with diethyl ether. The dark red solid was isolated by filtration and allowed to dry in air to give complex [Cu(**L**)(1,10-phenanthrolin-5,6-amine)](PF_6) (230 mg, 60% yield). ^{1}H NMR (CD_3CN): δ 8.75 (2H, d, J = 8.2 Hz); 8.37 (2H, dd, J = 8.5 Hz; J = 1.3 Hz); 8.30 (2H, dd, J = 4.6 Hz; J = 1.3 Hz); 8.24 (2H, s); 7.83 (2H, d, J = 8.2 Hz); 7.62 (2H, dd, J = 8.4 Hz; J = 4.6 Hz); 6.00 (4H, s); 4.42 (4H, s); 1.69 (12H, s); 1.64 (6H, s). ESI-MS (CH_3OH): calcd. [M-PF_6^-]$^+$ 689.25; obsd. 689.50. Anal. calcd. for [Cu(**L**)(1,10-phenanthrolin-5,6-diamine)](PF_6), $C_{42}H_{38}CuF_6N_6P·4.5H_2O$: C, 55.05; H, 5.17; N, 9.17. Found: C, 54.96; H, 4.88; N, 8.97.

[Cu(L)(2,9-dimethyl-1,10-phenanthrolin-5,6-amine)](PF_6): The same procedure as described for [Cu(**L**)(1,10-phenanthrolin-5,6-diamine)](PF_6) was followed using [Cu(CH_3CN)$_4$]PF_6 (205.6 mg, 0.55 mmol), 2,9-dimesityl-1,10-phenanthroline (229.8 mg, 0.55 mmol) and 2,9-dimethyl-6-nitro-1,10-phenanthrolin-5-amine (148 mg, 0.55 mmol) to afford [Cu(**L**)(2,9-dimethyl-6-nitro-1,10-phenanthrolin-5-amine)](PF_6) as an red solid (462 mg, 94% yield). ^{1}H NMR ($CDCl_3$): δ 8.99 (1H, d, J = 8.5 Hz); 8.91 (1H, d, J = 8.5 Hz); 8.68 (2H, d, J = 8.0 Hz); 8.22 (2H, s); 8.01 (2H, s); 7.81 (2H, d, J = 8.0 Hz); 7.72 (1H, d, J = 8.5 Hz); 7.44 (1H, d, J = 8.9 Hz); 6.32 (2H, s); 6.25 (2H, s); 2.23 (3H, s); 2.11 (3H, s); 1.92 (6H, s); 1.67 (6H, s); 1.65 (6H, s). [Cu(**L**)(2,9-dimethyl-6-nitro-1,10-phenanthrolin-5-amine)](PF_6) was used without further purification for the next synthetic step. [Cu(**L**)(6-Nitro-1,10-phenanthrolin-5-amine)](PF_6) (462 mg, 0.52 mmol) was reacted with d palladium on carbon (10%; 400 mg) and hydrazine (4 ml) affording [Cu(**L**)(2,9-dimethyl-1,10-phenanthrolin-5,6-amine)](PF_6) as a dark red solid (263 mg, 59% yield). ^{1}H NMR (CD_3CN): δ 8.75 (2H, d, J = 8.2 Hz); 8.27 (2H, d, J = 8.6 Hz); 8.25 (2H, s); 7.84 (2H, d, J = 8.2 Hz); 7.46 (2H, d, J = 8.6 Hz); 6.13 (4H, s); 4.34 (4H, s); 2.12 (6H, s); 1.78 (6H, s); 1.61 (12H, s). ESI-MS (CH_3OH): calcd. [M-PF_6^-]$^+$ 717.28; obsd. 717.58. Anal. calcd. for [Cu(**L**)(2,9-dimethyl-1,10-phenanthrolin-5,6-amine)](PF_6), $C_{44}H_{42}CuF_6N_6P·0.5H_2O$: C, 60.58; H, 4.97; N, 9.63. Found: C, 60.49; H, 4.73; N, 9.83.

RuH_2': [Ru(bpy)$_2$(5,6-dione-1,10-phenanthroline)](PF_6)$_2$ (973 mg, 1.07 mmol) and 2,9-dimethyl-1,10-phenanthroline-5,6-diamine (279.2 mg, 1.17 mmol) was added to a round bottom flask, suspended in an acetonitrile/ethanol mixture (5:1, 120 mL), and refluxed for 72 hours. After evaporation of the solvent the red residue was dissolved in acetonitrile, filtered and precipitated with diethyl ether. Chromatography on alumina (neutral), eluting with acetonitrile afforded the product as an orange solid in 57% yield (683 mg). ^{1}H NMR (CD_3CN): δ 9.57 (2H, d, J = 8.1 Hz); 9.48 (2H, d, J = 7.7 Hz, 2H); 8.57 (2H, d, J = 8.3 Hz); 8.55 (2H, d, J = 8.3 Hz); 8.31 (2H, dd, J = 5.3Hz, J = 1.2 Hz); 8.18-8.02 (6H, m); 7.90-7.84 (4H, m); 7.73 (2H, d, J = 8.2 Hz); 7.51 (2H, dt, J = 5.6 Hz, J = 1.3 Hz); 7.41 (2H, t, J = 6.0 Hz); 2.36 (6H, s). Anal. calcd. for **RuH_2'**, $C_{46}H_{32}F_{12}N_{10}P_2Ru·H_2O$: C, 48.73; H, 3.02; N, 12.35. Found: C, 48.54; H, 2.82; N, 12.08.

CuH_2-CuH_2: [Cu(CH_3CN)$_4$]PF_6 (89.5 mg, 0.24 mmol) was added to a round bottom flask and dissolved in dichloromethane (10 mL) with stirring. The clear colorless solution was deaerated with N_2. A similarly deaerated solution of **L** (100 mg, 0.24 mmol) in dichloromethane (5mL) was added to the reaction mixture. Upon addition of **L**, the solution turned bright yellow and was allowed to stir at room temperature for five minutes. **L1** (46.1 mg, 0.12 mmol) was then added followed by dichloromethane (5 mL). The red solution was allowed to stir under N_2 at room temperature for 2.5 hours. The product was precipitated with diethyl ether. The red solid was isolated by filtration and allowed to dry in air to give complex **CuH_2-CuH_2** (66 mg, 80% yield). ^{1}H NMR ((CD_3)CO): δ 10.19 (4H, d, J = 7.4 Hz); 9.14 (4H, d, J = 3.9 Hz); 9.02 (4H, d, J = 8.2 Hz); 8.46 (4H, s); 8.30 (4H, dd, J = 8.0 Hz, J = 4.7 Hz); 8.07 (4H, d, J = 8.2 Hz); 6.12 (8H, s); 1.88 (24H, s); 1.38 (12H, s). Anal. calcd. for **CuH_2-CuH_2**, $C_{84}H_{68}Cu_2F_{12}N_{10}P_2$: C, 61.72; H, 4.19; N, 8.57. Found: C, 61.60; H, 4.19; N, 8.47.

CuH_2-$CuMe_2$, method A: The same procedure as described for complex **CuH_2-CuH_2** was followed using $[Cu(CH_3CN)_4]PF_6$ (89.5 mg, 0.24 mmol), **L** (100 mg, 0.24 mmol) and **L2** (49.5 mg, 0.12 mmol) to afford an orange/red solid (153mg, 77% yield).

CuH_2-$CuMe_2$, method B: [Cu(**L**)(2,9-dimethyl-1,10-phenanthroline-5,6-dione)](PF_6) (51.7 mg, 0.060 mmol) and [Cu(**L**)(1,10-phenanthroline-5,6-diamine)](PF_6) (50.0 mg, 0.060 mmol) were suspended in ethanol (20 mL). Three drops of acetic acid were added and the reaction was refluxed for 72 hours. The reaction mixture was allowed to cool to room temperature and kept at -20°C overnight**.** Filtration and washing with ethanol and diethyl ether afforded the product as red powder in 55% yield (55 mg).

^{1}H NMR ((CD_3)CO): δ 10.17 (2H, d, J = 8.3 Hz); 10.07 (2H, d, J = 8.2 Hz); 9.13 (2H, d, J = 3.8 Hz); 9.05 (2H, d, J = 8.2 Hz); 9.01 (2H, d, J = 8.2 Hz); 8.49 (s, 2H); 8.46 (s, 2H); 8.28 (2H, dd, J = 8.1 Hz, J = 4.7 Hz); 8.14 (2H, d, J = 8.3 Hz); 8.09 (2H, d, J = 8.1 Hz); 8.07 (2H, d, J = 8.1 Hz); 6.25 (4H, s); 6.11 (4H, s); 2.49 (6H, s); 1.87 (12H, s); 1.78 (12H, s); 1.61 (6H, s); 1.39 (6H, s). Anal. calcd. for **CuH_2-$CuMe_2$**, $C_{86}H_{72}Cu_2F_{12}N_{10}P_2\cdot CH_2Cl_2\cdot 2H_2O$: C, 58.59; H, 4.41; N, 7.85. Found: C, 58.25; H, 4.18; N, 8.23.

$CuMe_2$-$CuMe_2$, method A: The same procedure as described for complex **CuH_2-CuH_2** was followed using $[Cu(CH_3CN)_4]PF_6$ (84.6 mg, 0.23 mmol), **L** (94.6 mg, 0.23 mmol) and **L3** (50 mg, 0.11 mmol) to afford an orange/red solid (146mg, 76% yield).

$CuMe_2$-$CuMe_2$, method B: [Cu(**L**)(2,9-dimethyl 1,10-phenanthroline-5,6-dione-)](PF_6) (50.0 mg, 0.058 mmol) and [Cu(**L**)(2,9-dimethyl1,10-phenanthroline-5,6-diamine)](PF_6) (50.0mg, 0.058 mmol) were suspended in ethanol (20 mL). Three drops acetic acid were added and the reaction was refluxed for 48 hours. The reaction mixture was allowed to cool to room temperature and kept at -20°C overnight**.** Filtration and washing with ethanol and diethyl ether afforded the product as red powder in 73% yield (71 mg).

^{1}H NMR ((CD_3)CO): δ 10.05 (4H, d, J = 8.5 Hz); 9.04 (4H, d, J = 8.5 Hz); 8.48 (s, 4H), 8.11 (4H, d, J = 8.0 Hz); 8.07 (4H, d, J = 8.0 Hz); 6.26 (8H, s); 2.47 (12H, s); 1.78 (24H, s); 1.63 (12H, s). Anal. calcd. for **$CuMe_2$-$CuMe_2$**, $C_{88}H_{76}Cu_2F_{12}N_{10}P_2\cdot 1.5CH_2Cl_2\cdot H_2O$: C, 58.09; H, 4.35; N, 7.74. Found: C, 58.05; H, 4.44; N, 8.06.

CuH_2-RuH_2: $[Cu(CH_3CN)_4]PF_6$ (30.0 mg, 0.064 mmol) was added to a round bottom flask and dissolved in dichloromethane (10 mL) with stirring. The clear colorless solution was deaerated with N_2. A similarly deaerated solution of **L** (26.8 mg, 0.064 mmol) in dichloromethane (5mL) was added to the reaction mixture. Upon addition of **L** the solution turned bright yellow, and was allowed to stir at room temperature for five minutes. **RuH_2** (70.0 mg, 0.064 mmol) was then added followed by dichloromethane (5 mL). The red solution was allowed to stir under N_2 at room temperature for two hours. The mixture was filtered and concentrated and the product was precipitated with diethyl ether. The red solid was isolated by filtration and allowed to dry in air to give complex **CuH_2-RuH_2** (97 mg, 88% yield). Single crystals suitable for X-ray structure analysis were obtained via diffusion of diethyl ether into a concentrated acetonitrile product solution. ^{1}H NMR ((CD_3)$_2$CO): δ 10.22 (2H, d, J = 8.3 Hz); 10.15 (2H, d, J = 7.6 Hz); 9.12 (2H, d, J = 3.9 Hz); 9.00 (2H, d, J = 8.2 Hz); 8.92 (2H, d, J = 8.3 Hz); 8.88 (2H, d, J = 7.9 Hz); 8.68 (2H, d, J = 5.2 Hz); 8.44 (2H, s), 8.31 (2H, t, J = 8.1 Hz); 8.28-8.17 (8H, m); 8.13 (2H, d, J = 5.2 Hz); 8.05 (2H, d, J = 8.2 Hz); 7.69 (2H, t, J = 6.8 Hz); 7.45 (2H, t, J = 6.6 Hz); 6.08 (2H, s); 6.07 (2H, s); 1.85 (12H, s); 1.32 (6H, s). Anal. calcd. for **CuH_2-RuH_2**, $C_{74}H_{56}CuF_{18}N_{12}P_3Ru\cdot 3H_2O$: C, 50.30; H, 3.54; N, 9.51. Found: C, 50.28; H, 3.31; N, 9.64.

$CuMe_2$-RuH_2: The same procedure as described for complex **CuH_2-RuH_2** was followed using $[Cu(CH_3CN)_4]PF_6$ (23.4 mg, 0.063 mmol), **L** (26.1 mg, 0.063 mmol) and **RuH_2'** (70 mg, 0.063 mmol) to afford an orange/red solid (98 mg, 90% yield). ^1H NMR ($(CD_3)_2CO$): δ 10.20 (2H, dd, J = 8.2 Hz, J = 1.2 Hz); 10.03 (2H, d, J = 8.3 Hz); 9.03 (2H, d, J = 8.2 Hz); 8.91 (2H, d, J = 8.3 Hz); 8.88 (2H, d, J = 8.2 Hz); 8.66 (2H, dd, J = 5.3 Hz, J = 1.2 Hz); 8.47 (2H, s); 8.31 (2H, dt, J = 8.0 Hz, J = 1.4 Hz); 8.24-8.18 (6H, m), 8.13 (2H, d, J = 5.2 Hz); 8.10 (2H, d, J = 8.3 Hz); 8.06 (2H, d, J = 8.2 Hz); 7.69 (2H, dt, J = 7.6 Hz, J = 1.2 Hz); 7.45 (2H, dt, J = 7.2 Hz, J = 1.2 Hz); 6.21 (4H, s); 2.46 (6H, s); 1.76 (6H, s); 1.75 (6H, s); 1.56 (6H, s). Anal. calcd. for **$CuMe_2$-RuH_2**, $C_{76}H_{60}CuF_{18}N_{12}P_3Ru \cdot 3H_2O$: C, 50.82; H, 3.71; N, 9.36. Found: C, 50.91; H, 3.55; N, 9.21.

3. NMR spectra

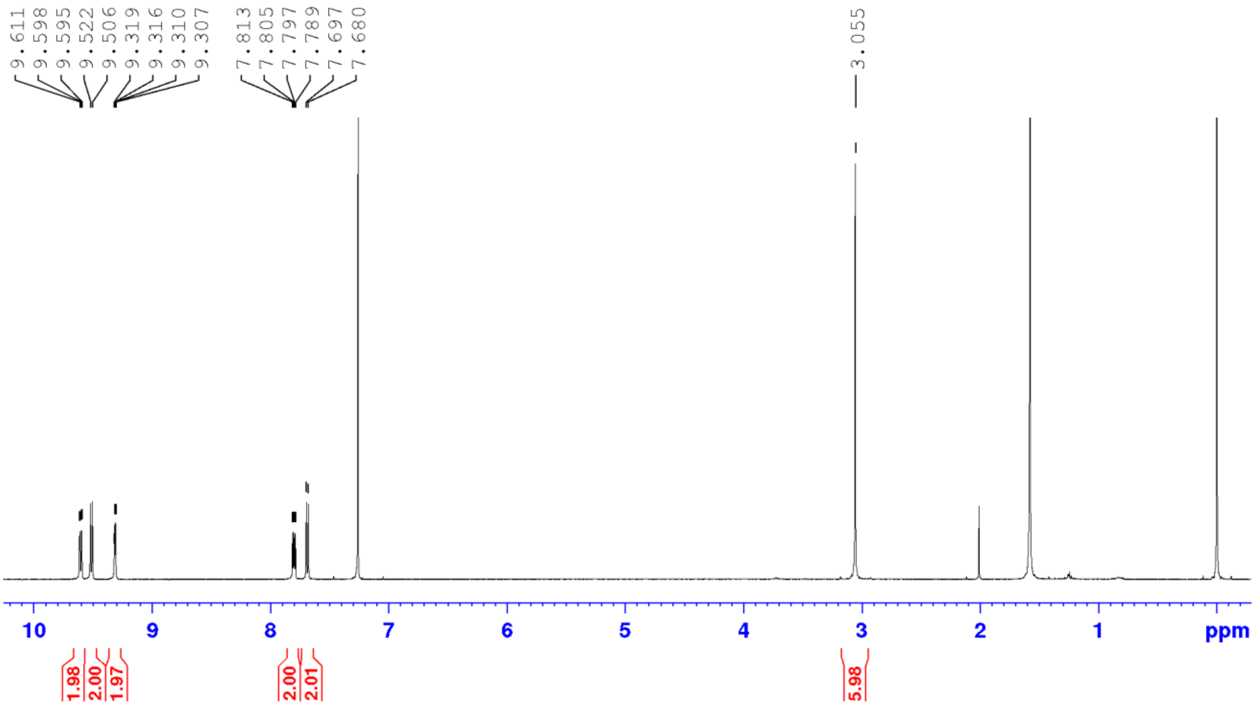

Figure S1. ^{1}H NMR spectrum of compound **L2** in $CDCl_3$.

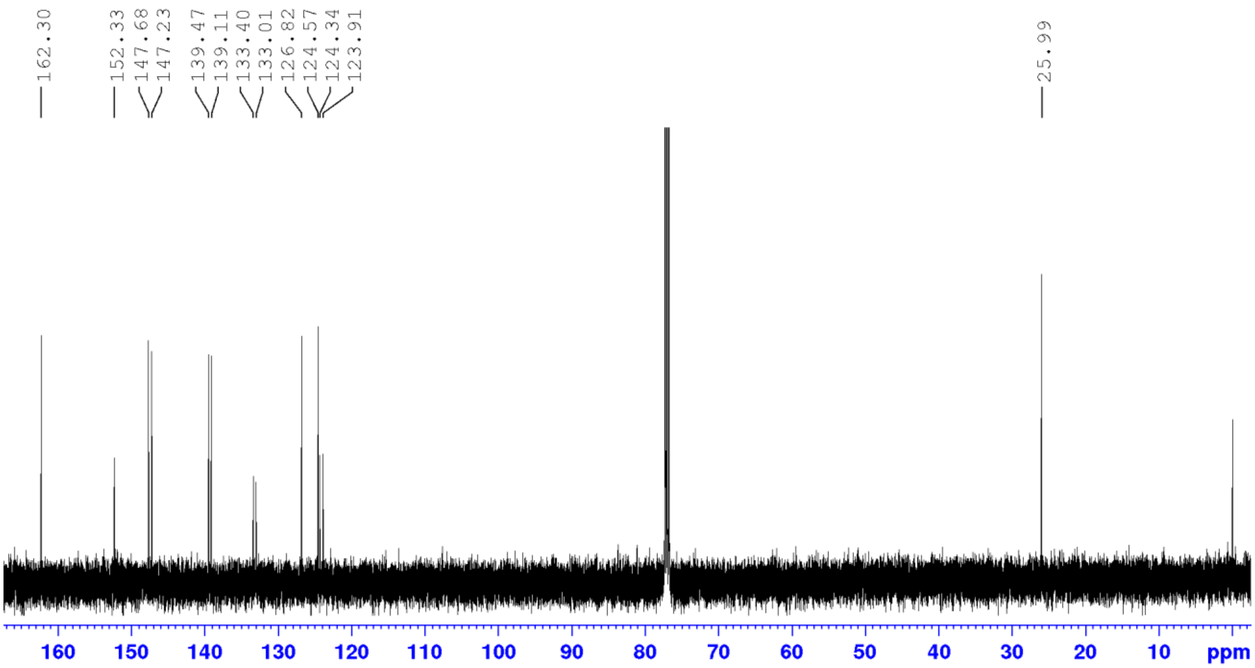

Figure S2. ^{13}C NMR spectrum of compound **L2** in $CDCl_3$.

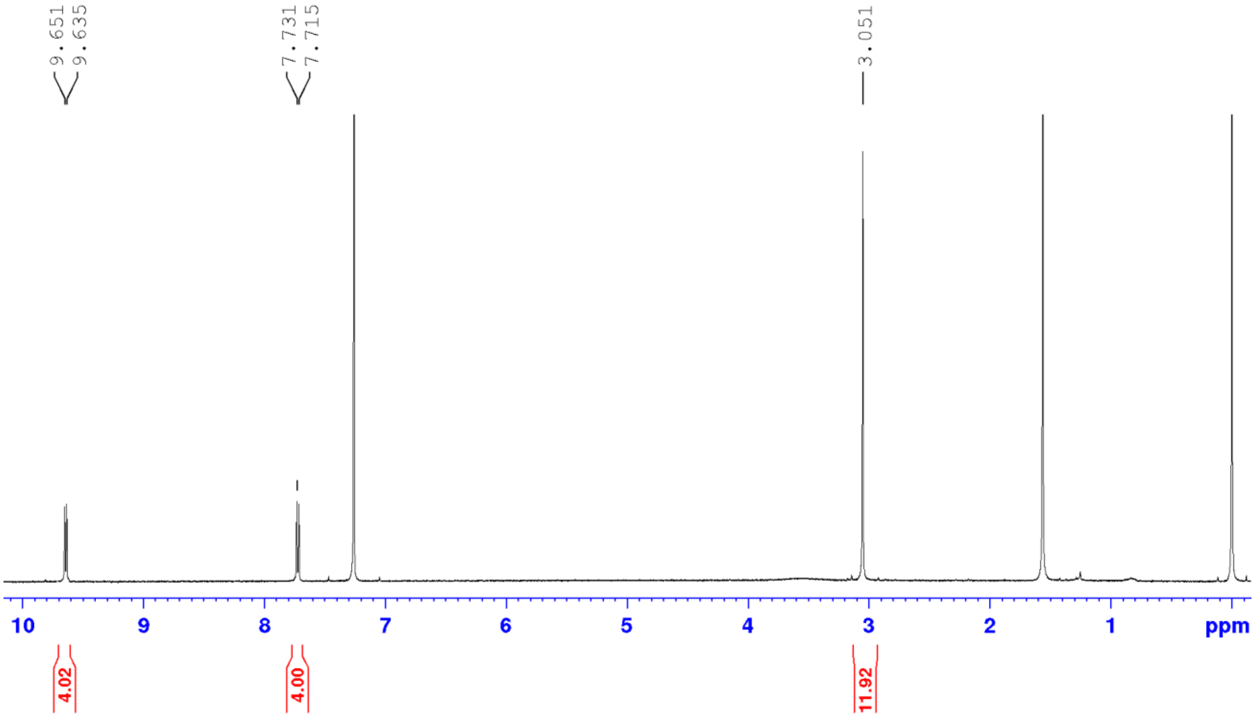

Figure S3. ^{1}H NMR spectrum of compound **L3** in $CDCl_3$.

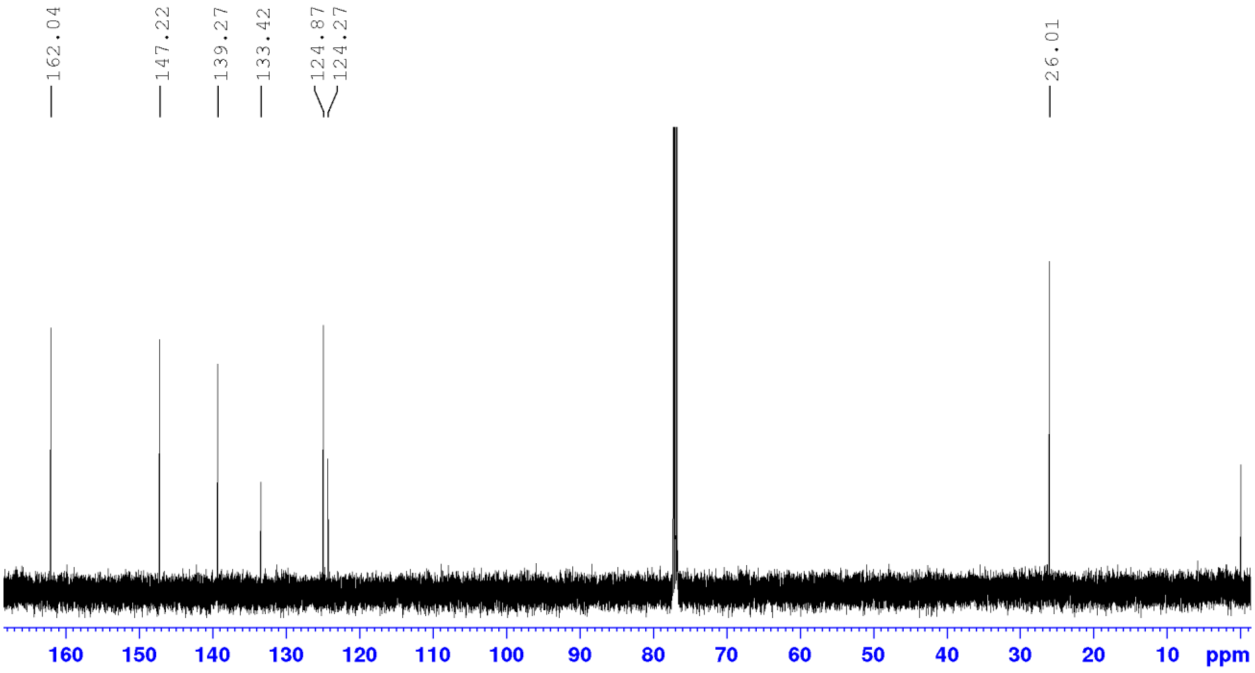

Figure S4. ^{13}C NMR spectrum of compound **L3** in $CDCl_3$.

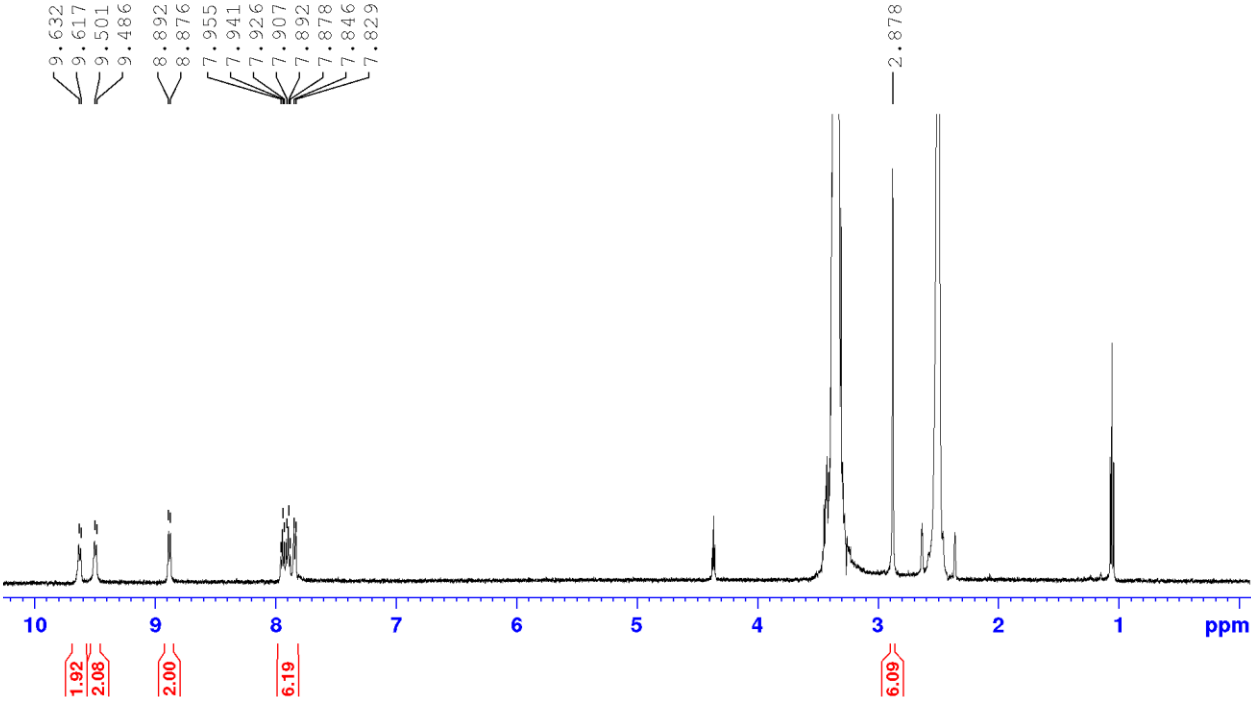

Figure S5. ^{1}H NMR spectrum of compound **L5** in $(CD_3)_2SO$.

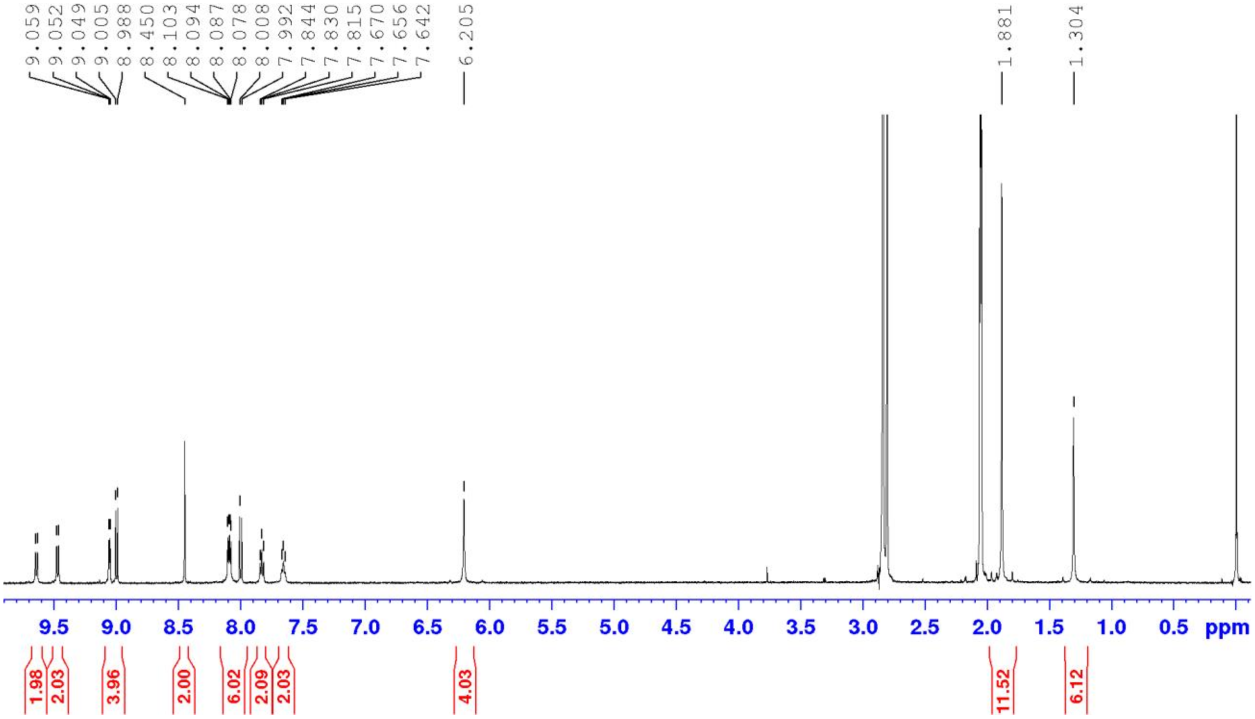

Figure S6. ^{1}H NMR of complex **CuH_2** in acetone-d_6.

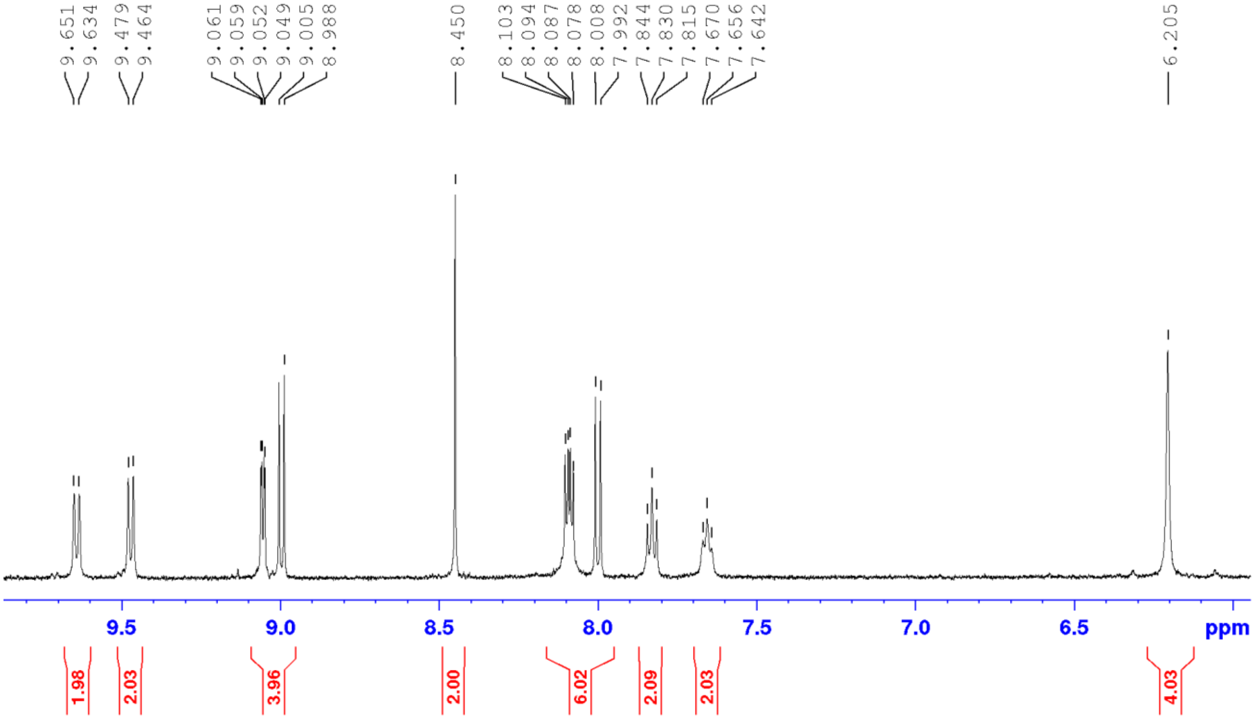

Figure S7. Aromatic region of ^{1}H NMR spectrum of complex **CuH_2** in acetone-d_6.

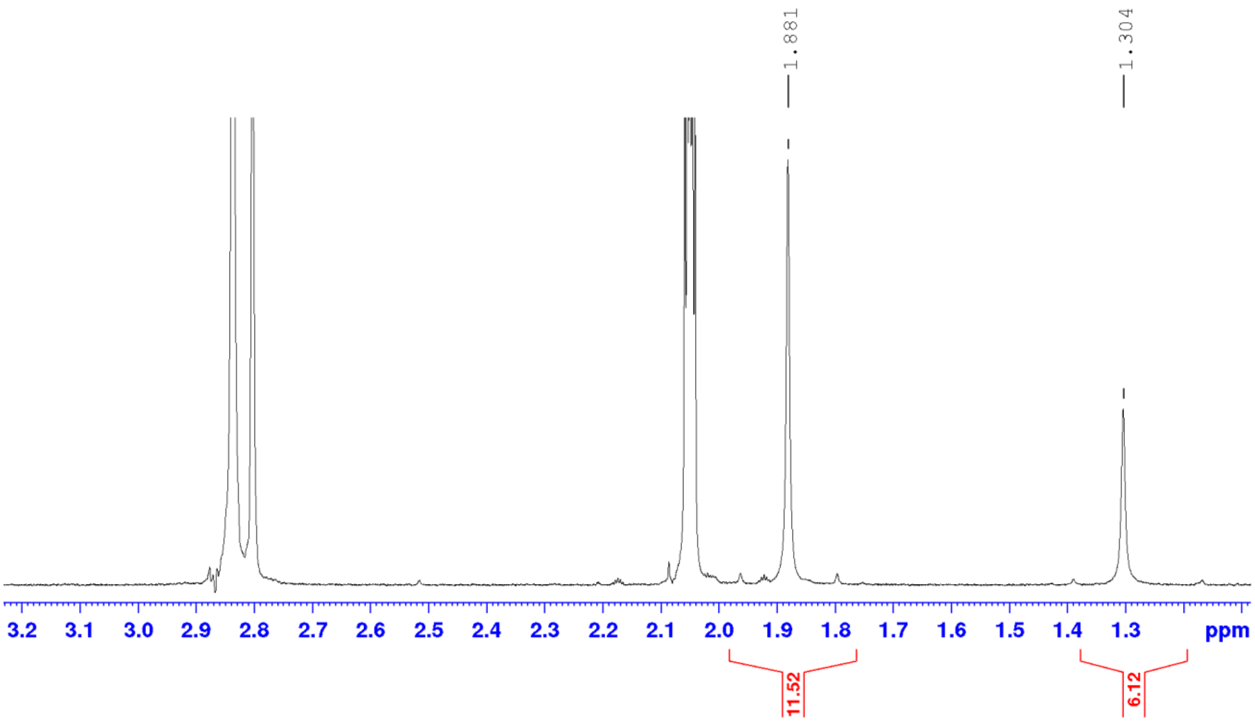

Figure S8. Aliphatic region of ^{1}H NMR spectrum of complex **CuH_2** in acetone-d_6.

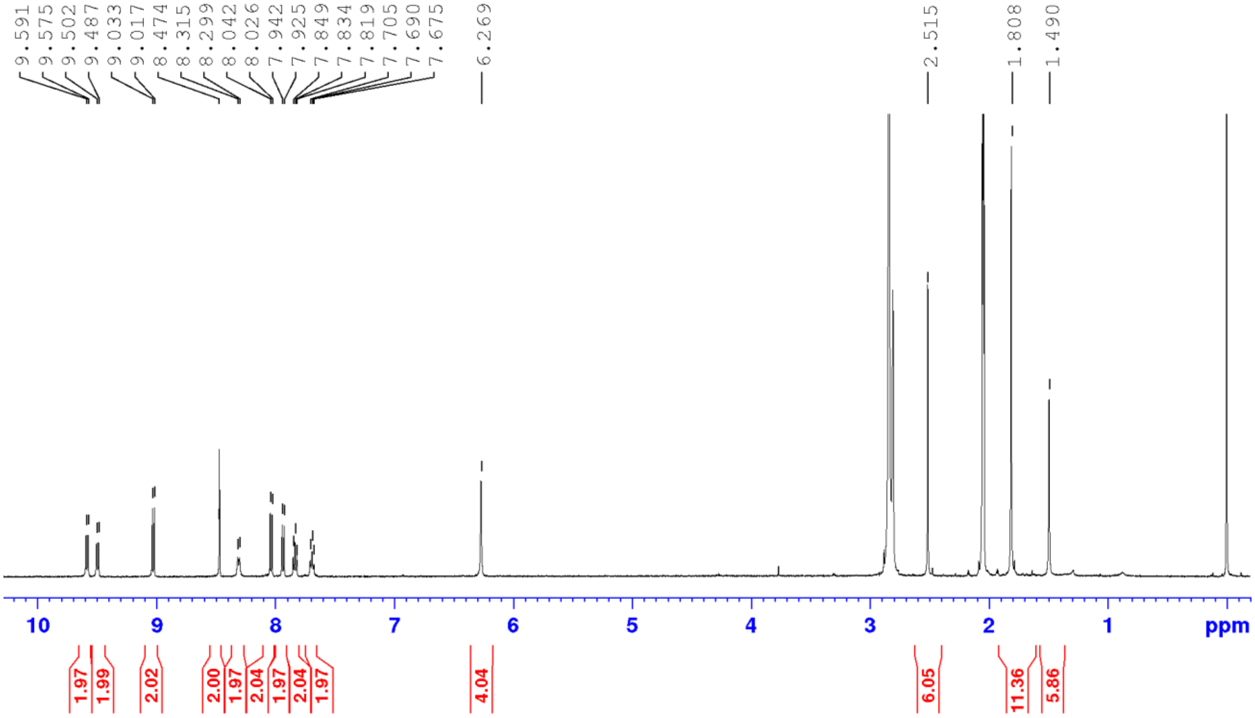

Figure S9. ^{1}H NMR of **$CuMe_2$** in acetone-d_6.

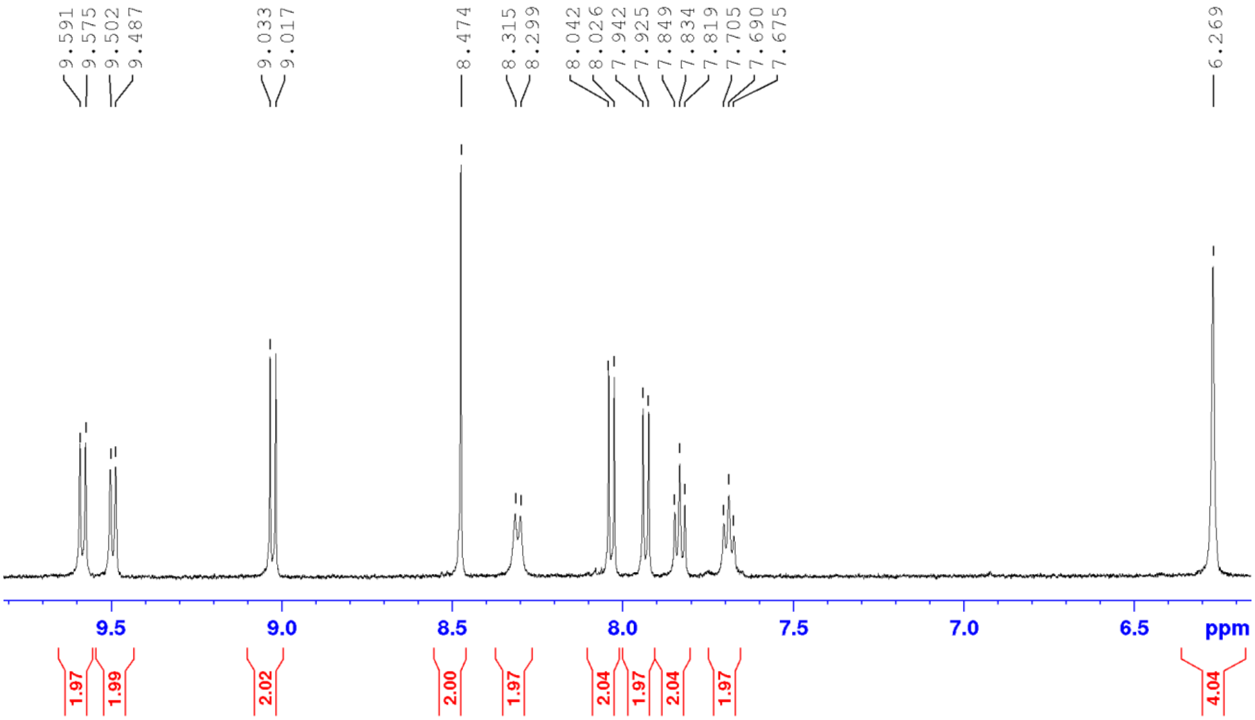

Figure S10. Aromatic region of ^{1}H NMR spectrum of **$CuMe_2$** in acetone-d_6.

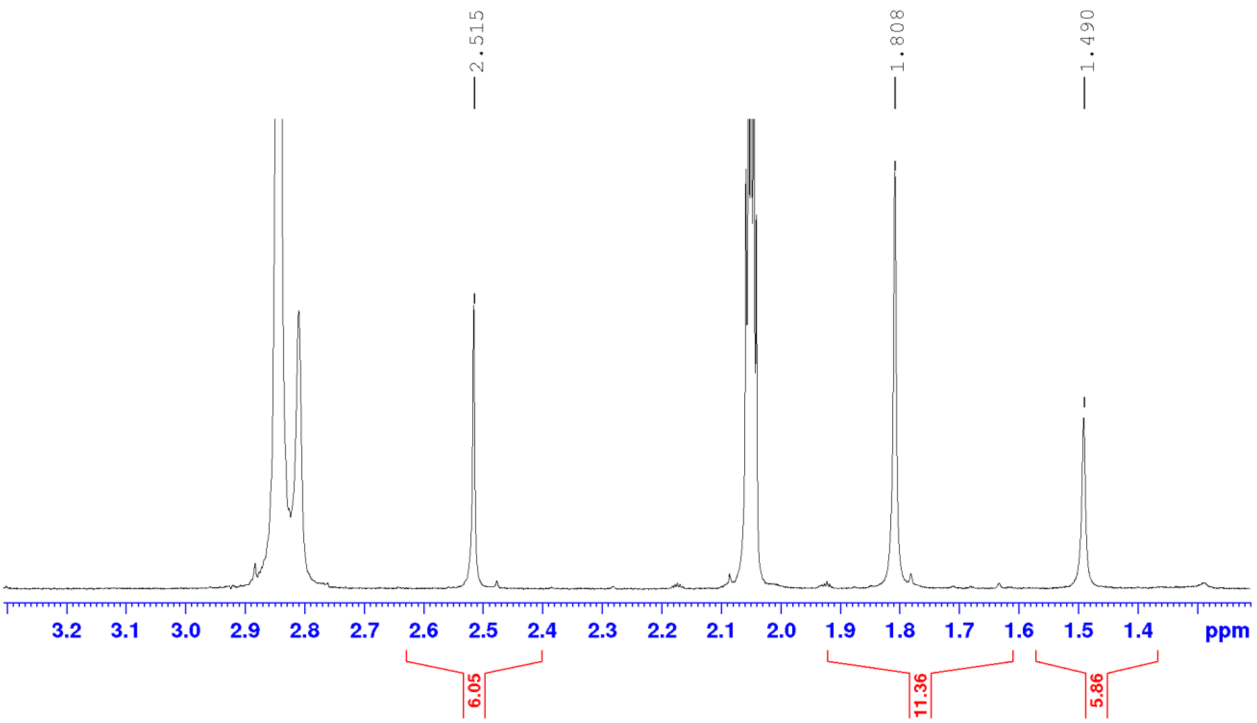

Figure S11. Aliphatic region of ^{1}H NMR spectrum of **$CuMe_2$** in acetone-d_6.

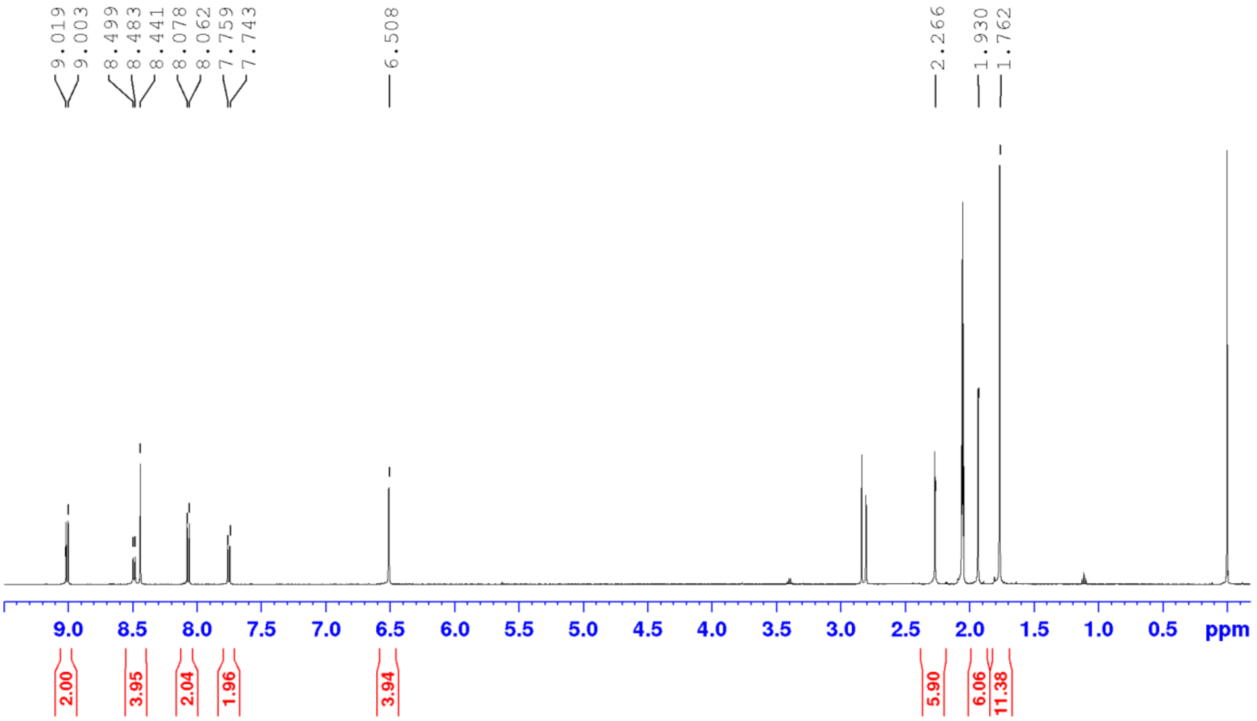

Figure S12. ^{1}H NMR of [Cu(**L**)(2,9-dimethyl-1,10-phenanthrolin-5,6-dione)](PF_6) in acetone-d_6.

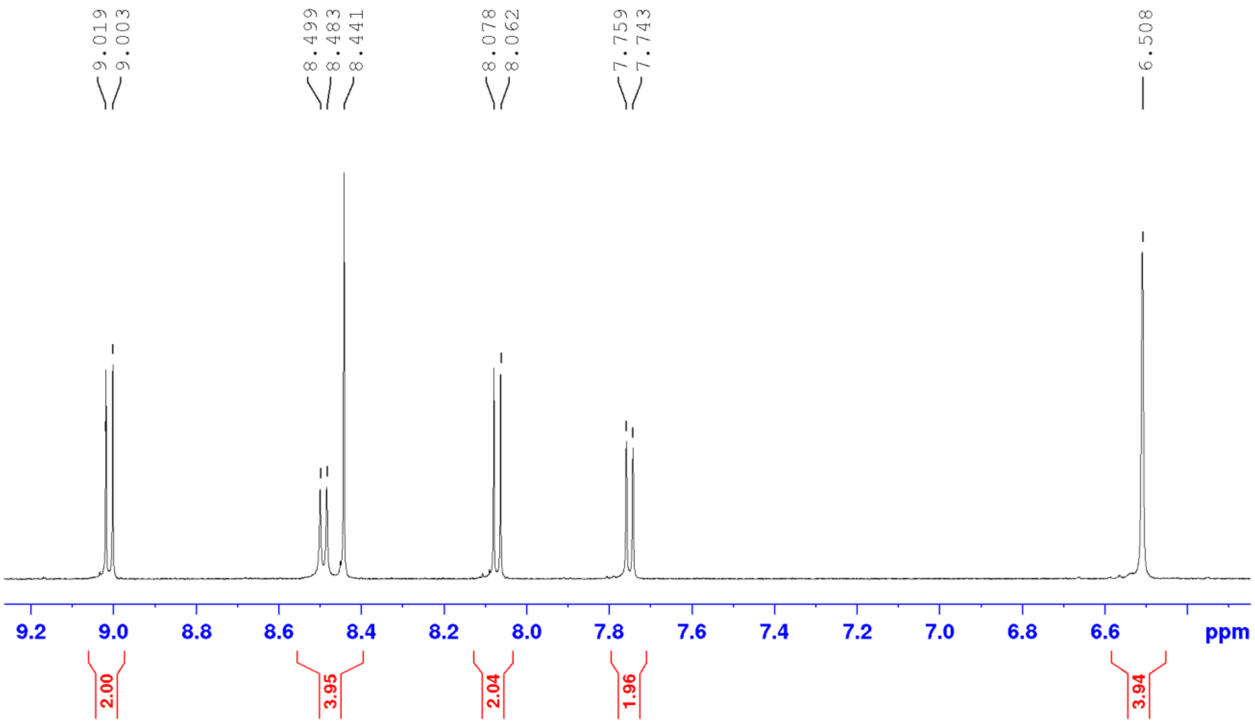

Figure S13. Aromatic region of ^{1}H NMR spectrum of [Cu(**L**)(2,9-dimethyl-1,10-phenanthrolin-5,6-dione)](PF_6) in acetone-d_6.

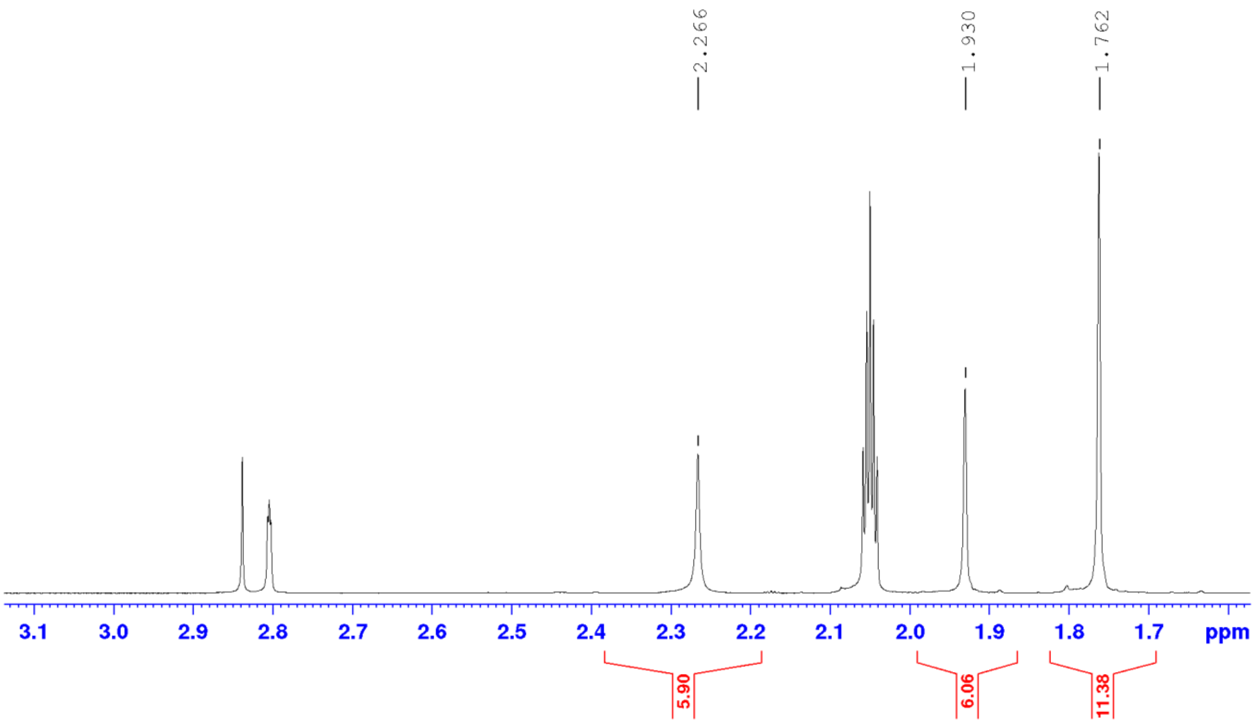

Figure S14. Aliphatic region of ^{1}H NMR spectrum of [Cu(**L**)(2,9-dimethyl-1,10-phenanthrolin-5,6-dione)](PF_6) in acetone-d_6.

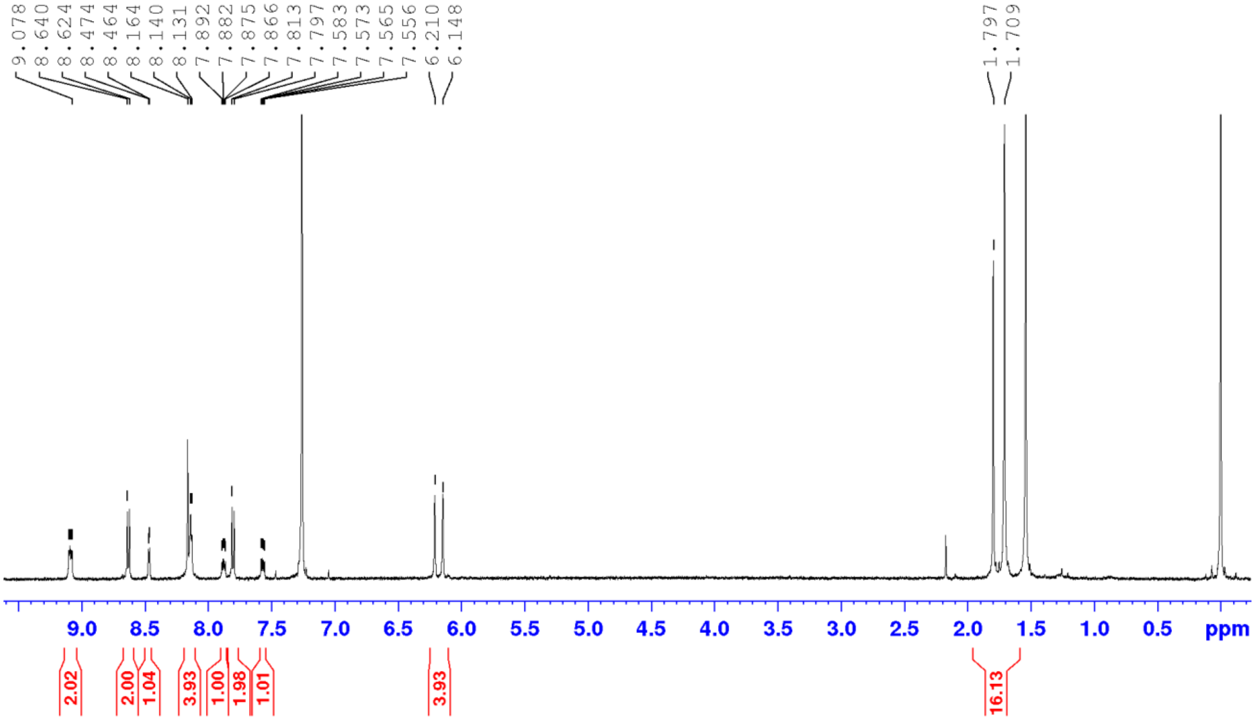

Figure S15. ^{1}H NMR spectrum of [Cu(**L**)(6-nitro-1,10-phenanthrolin-5-amine)](PF_6) in $CDCl_3$.

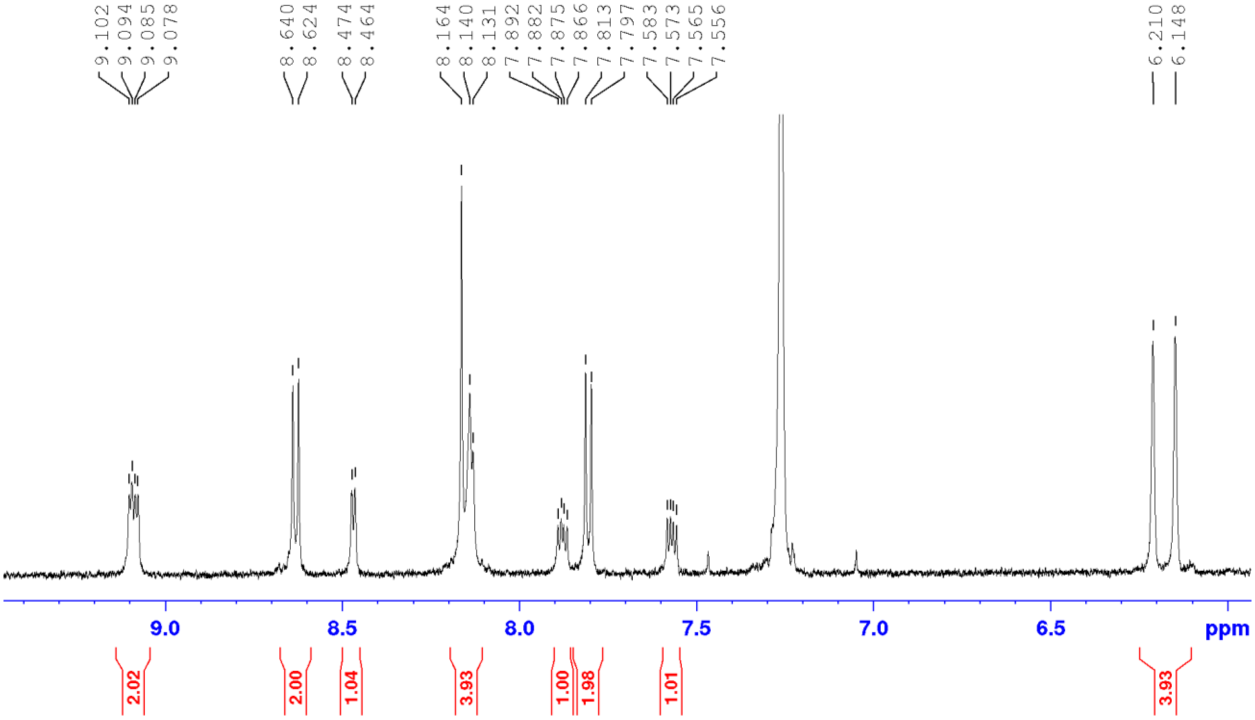

Figure S16. Aromatic region ^{1}H NMR of [Cu(**L**)(6-nitro-1,10-phenanthrolin-5-amine)](PF_6) in $CDCl_3$.

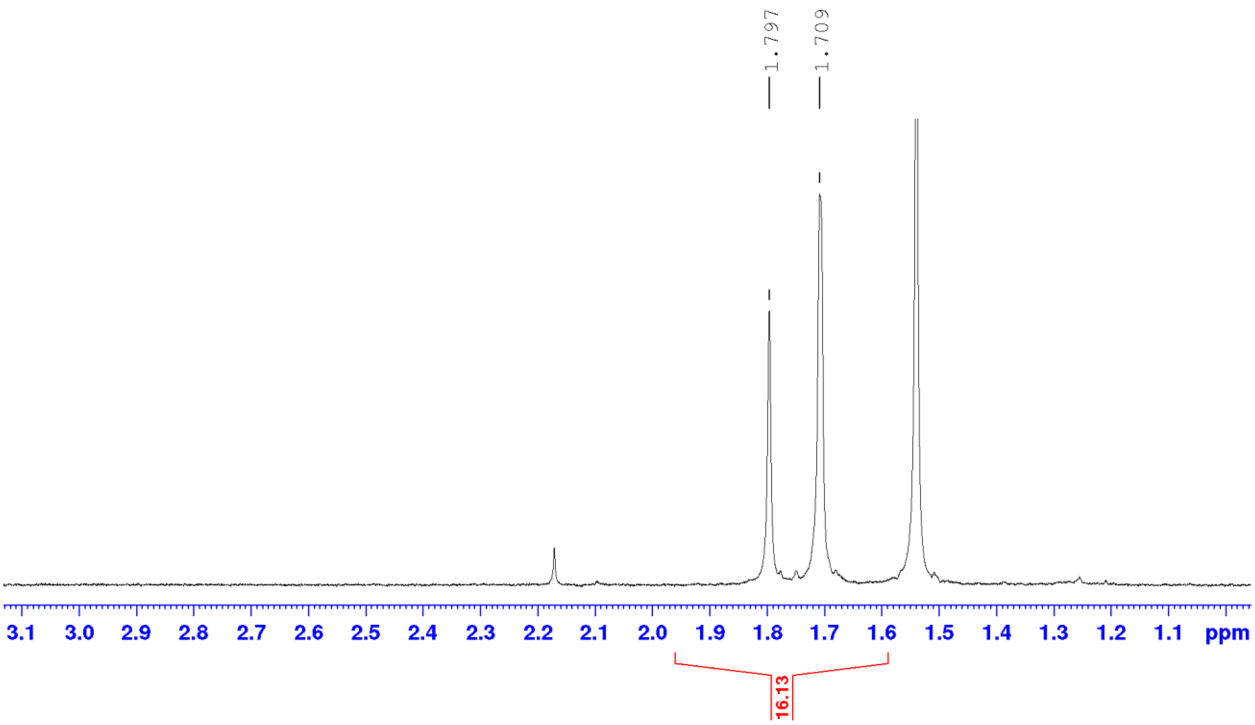

Figure S17. Aliphatic region of ^{1}H NMR spectrum of [Cu(**L**)(6-nitro-1,10-phenanthrolin-5-amine)](PF_6) in $CDCl_3$.

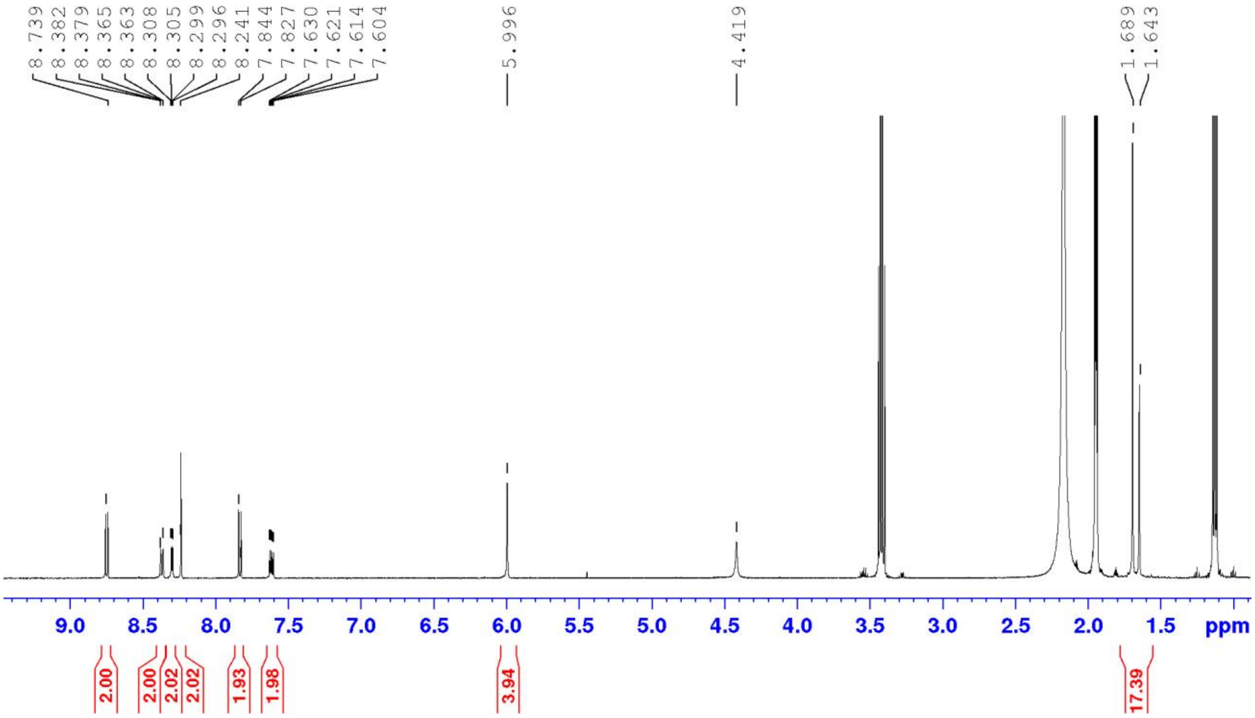

Figure S18. ^{1}H NMR spectrum of [Cu(**L**)(1,10-phenanthrolin-5,6-diamine)](PF_6) in CD_3CN.

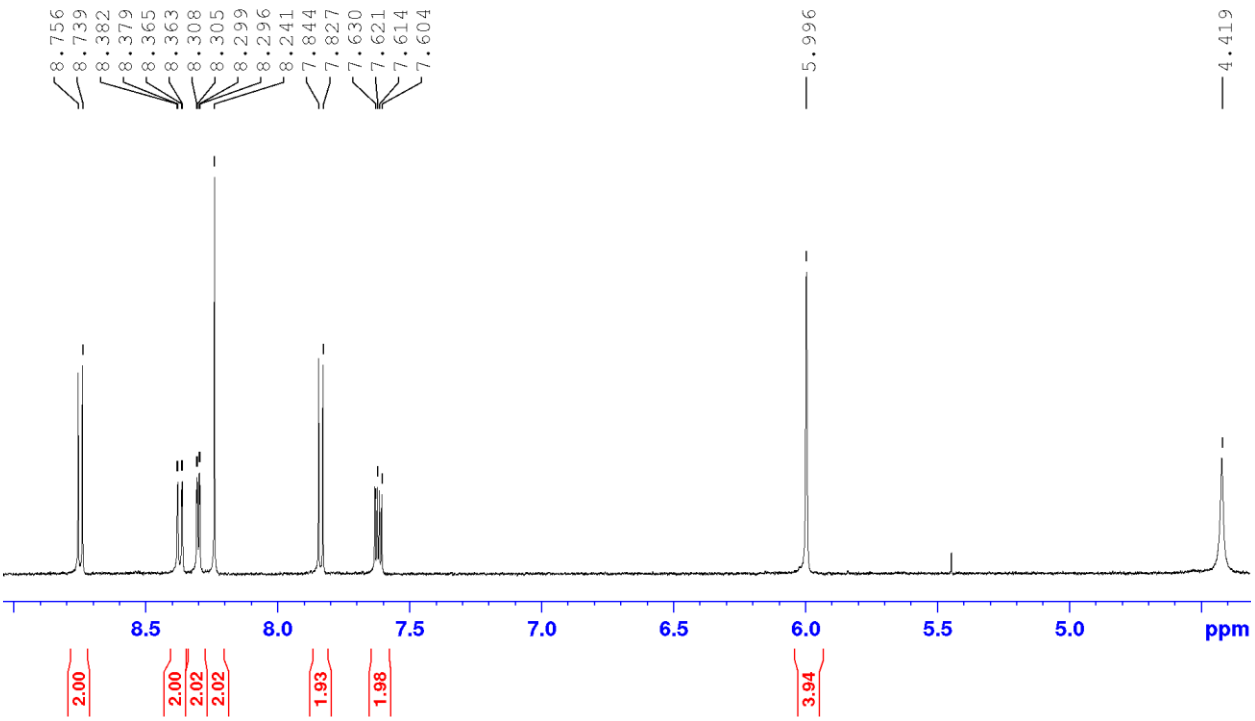

Figure S19. Aromatic region of ^{1}H NMR of [Cu(**L**)(1,10-phenanthrolin-5,6-diamine)](PF_6) in CD_3CN.

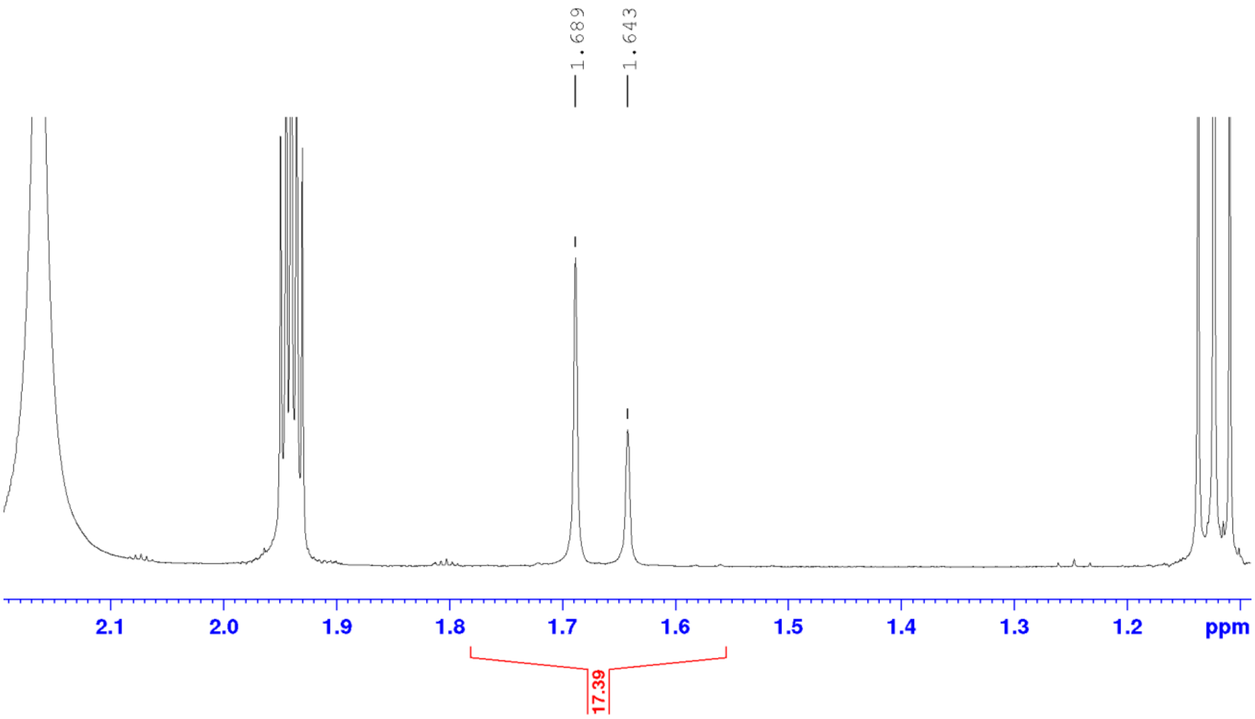

Figure S20. Aliphatic region of ^{1}H NMR spectrum of [Cu(**L**)(1,10-phenanthrolin-5,6-diamine)](PF_6) in CD_3CN.

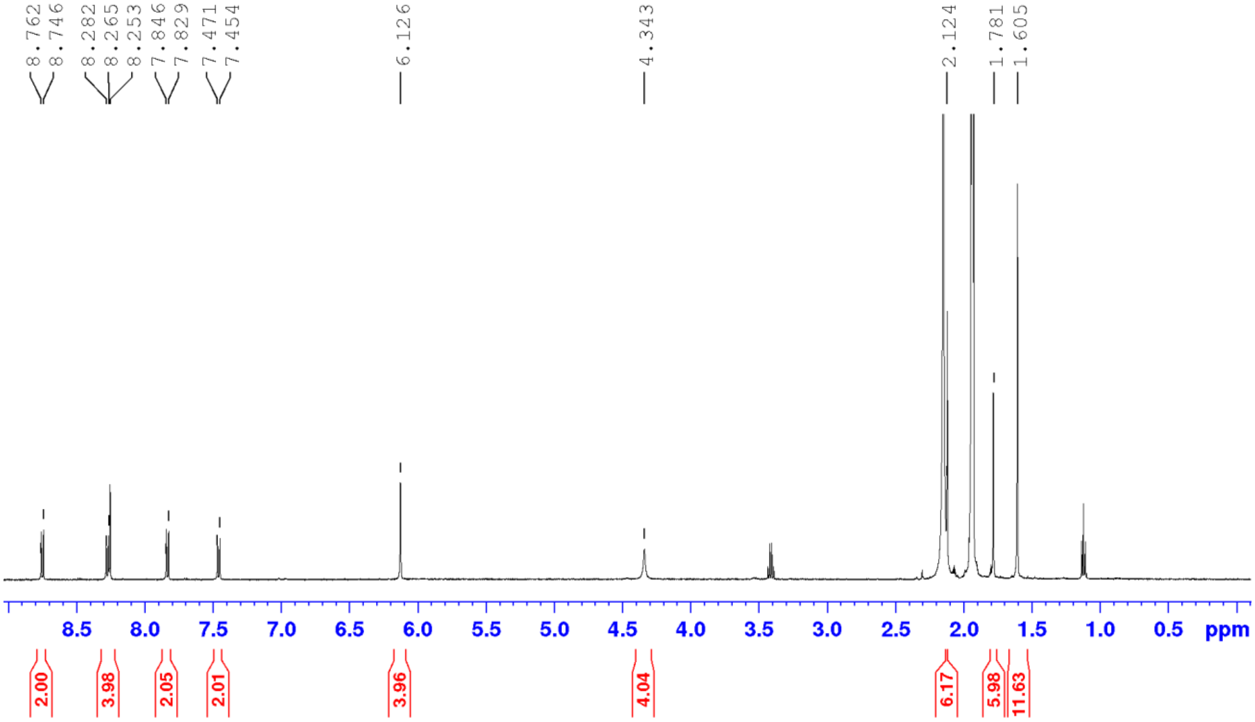

Figure S21. ^{1}H NMR spectrum of [Cu(**L**)(2,9-dimethyl-1,10-phenanthrolin-5,6-diamine)](PF_6) in CD_3CN.

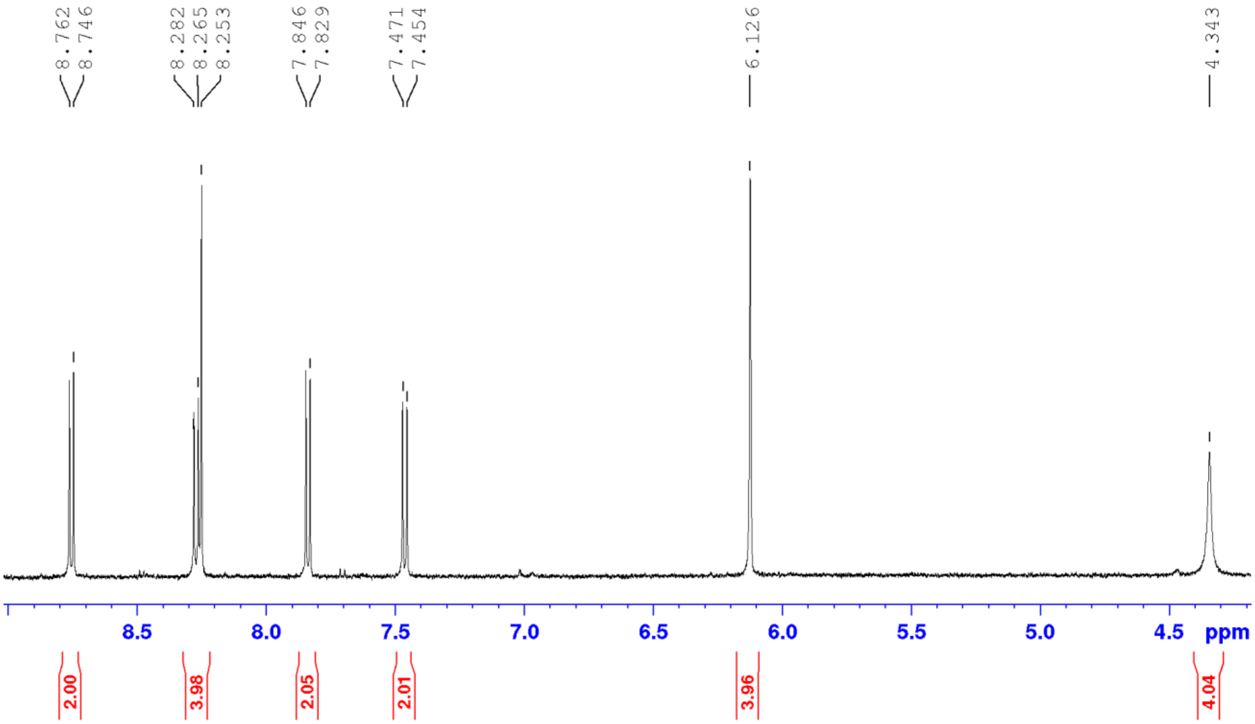

Figure S22. Aromatic region of ^{1}H NMR of [Cu(**L**)(2,9-dimethyl-1,10-phenanthrolin-5,6-diamine)](PF_6) in CD_3CN.

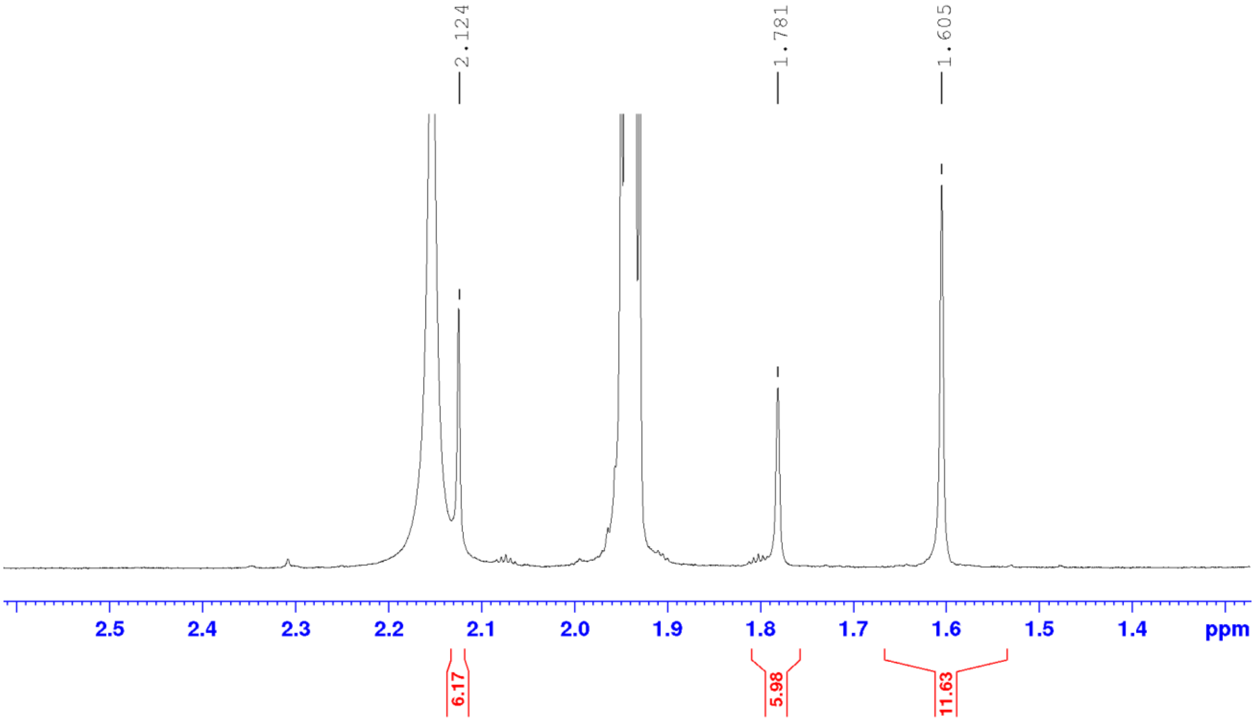

Figure S23. Aromatic region of ^{1}H NMR spectrum of [Cu(**L**)(2,9-dimethyl-1,10-phenanthrolin-5,6-diamine)](PF_6) in CD_3CN.

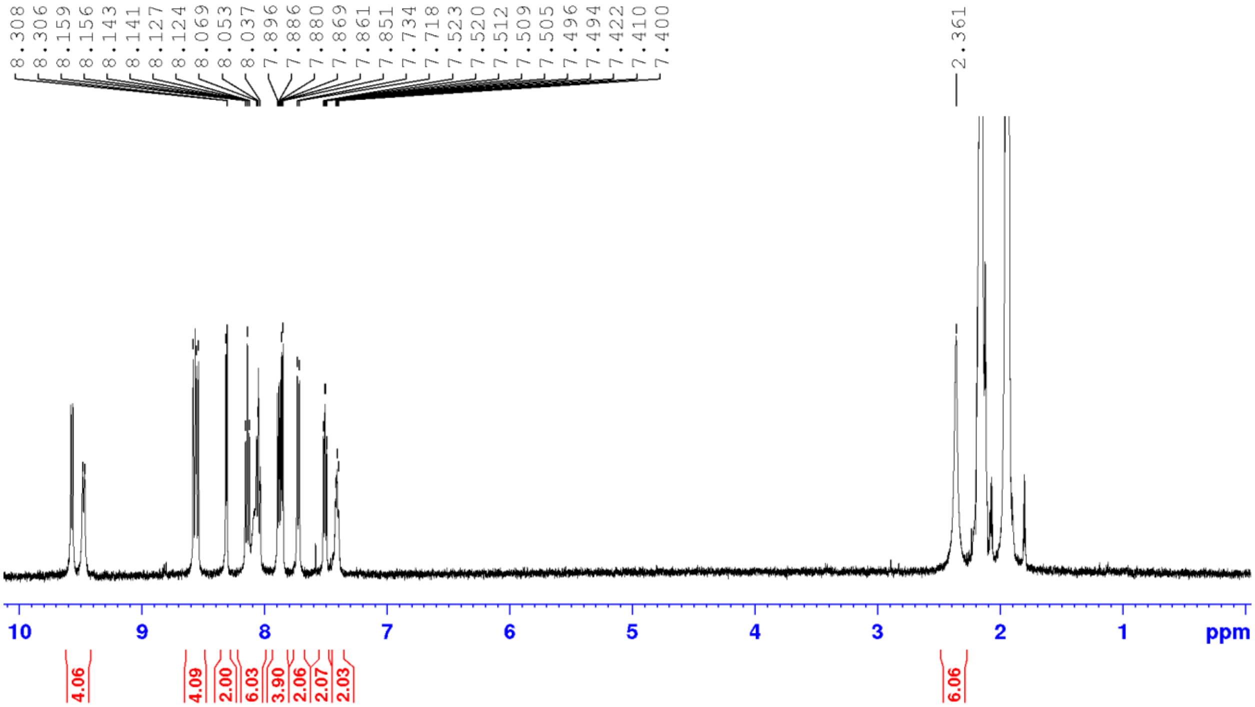

Figure S24. ^{1}H NMR spectrum of **RuH₂’** in CD_3CN.

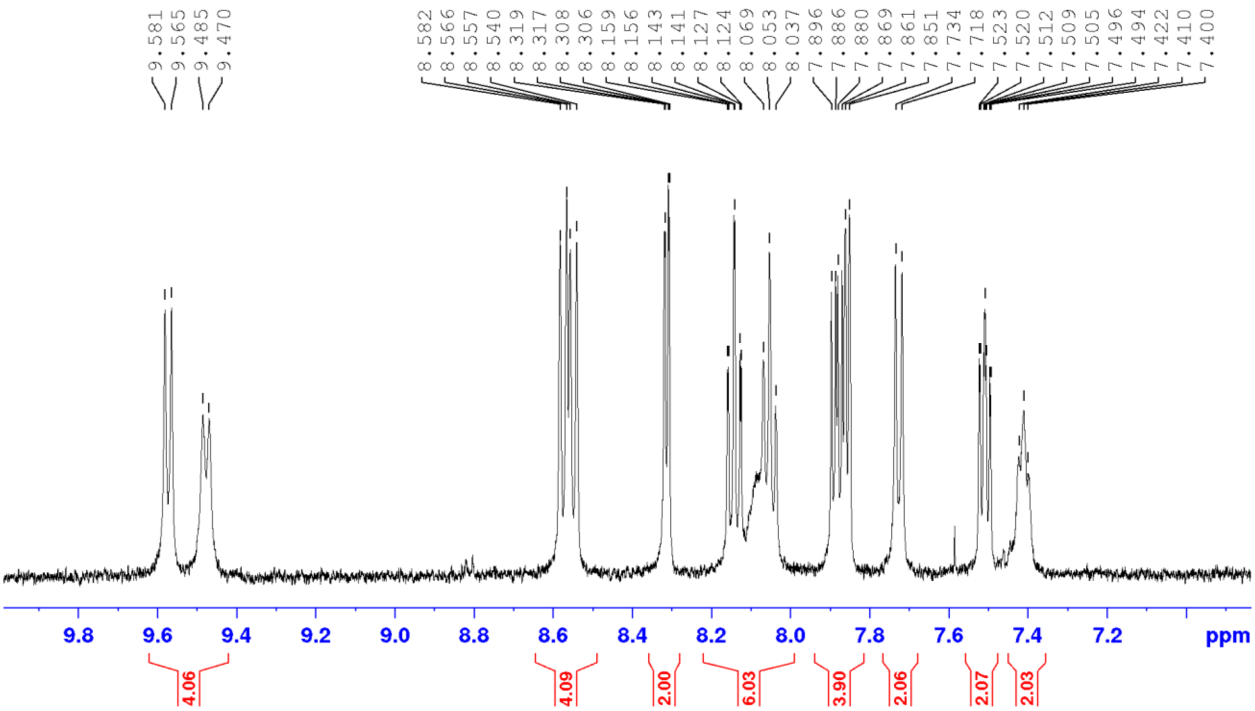

Figure S25. Aromatic region ^{1}H NMR of complex **RuH$_2$'** in CD_3CN.

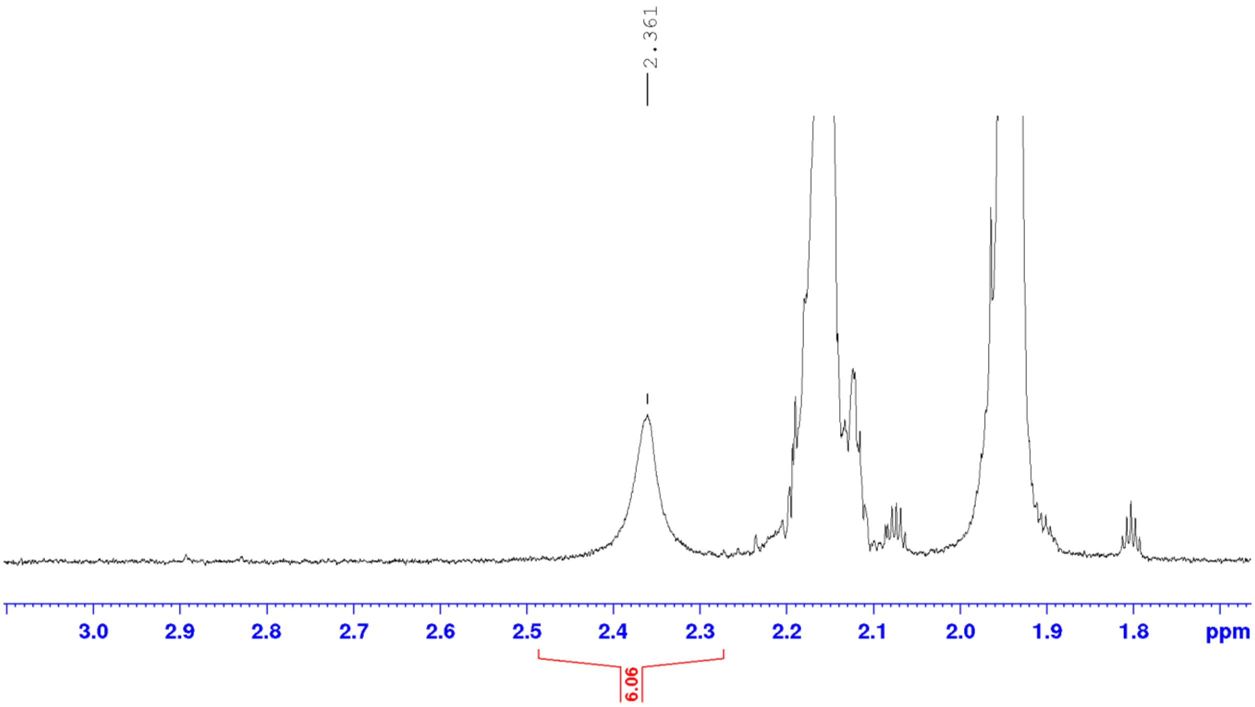

Figure S26. Aliphatic region of ^{1}H NMR spectrum of **RuH$_2$'** in CD_3CN.

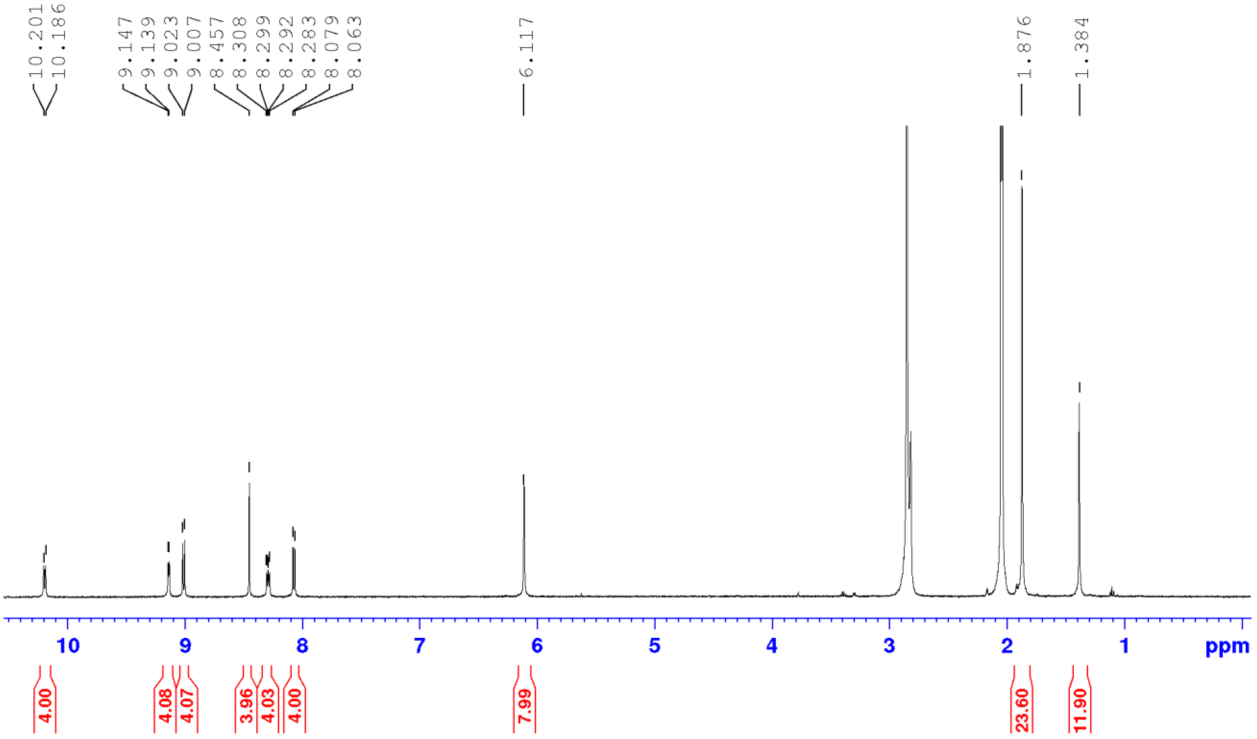

Figure S27. ${}^{1}H$ NMR of **CuH_2-CuH_2** in acetone-d_6.

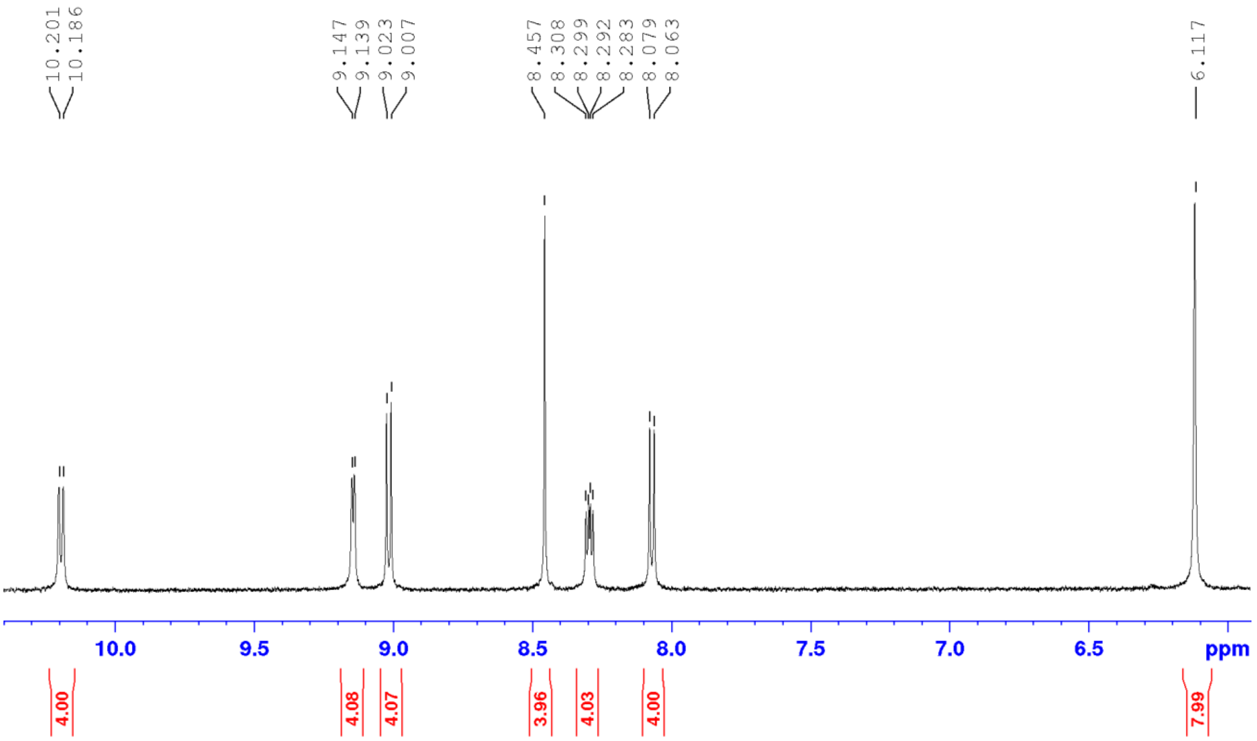

Figure S28. Aromatic region of ${}^{1}H$ NMR spectrum of **CuH_2-CuH_2** in acetone-d_6.

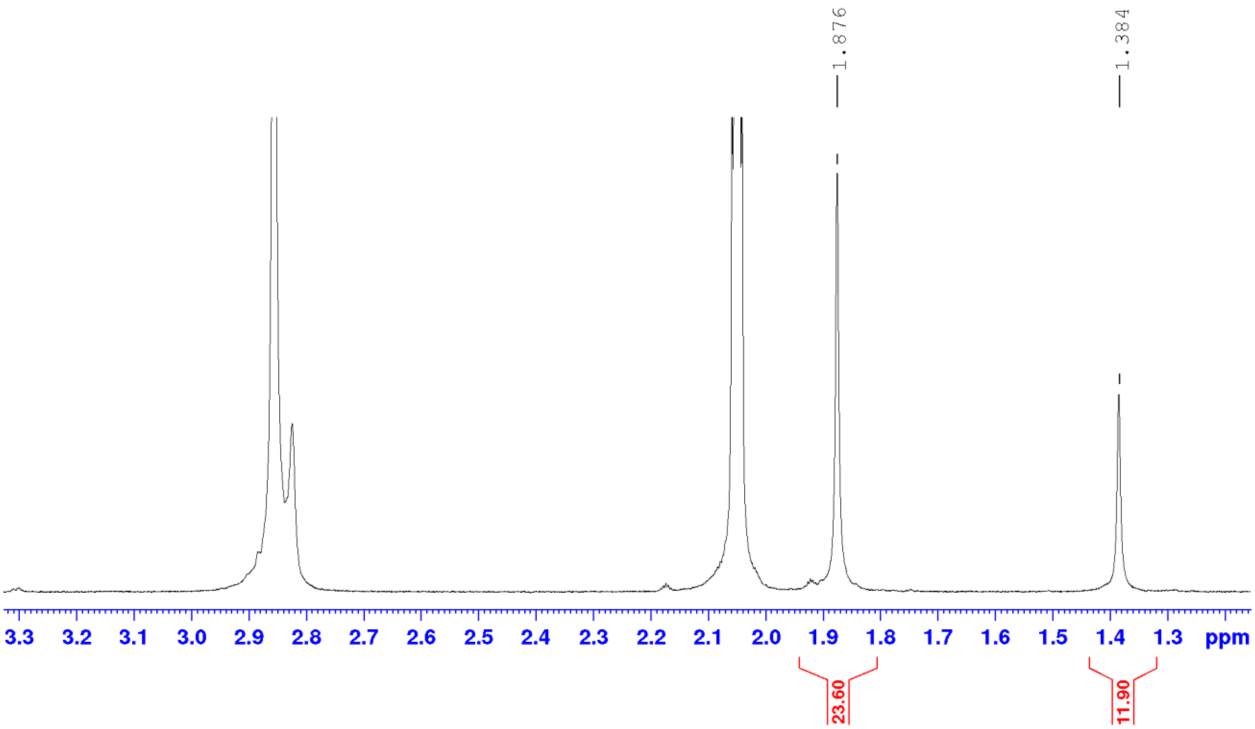

Figure S29. Aliphatic region of ^{1}H NMR spectrum of **CuH_2-CuH_2** in acetone-d_6.

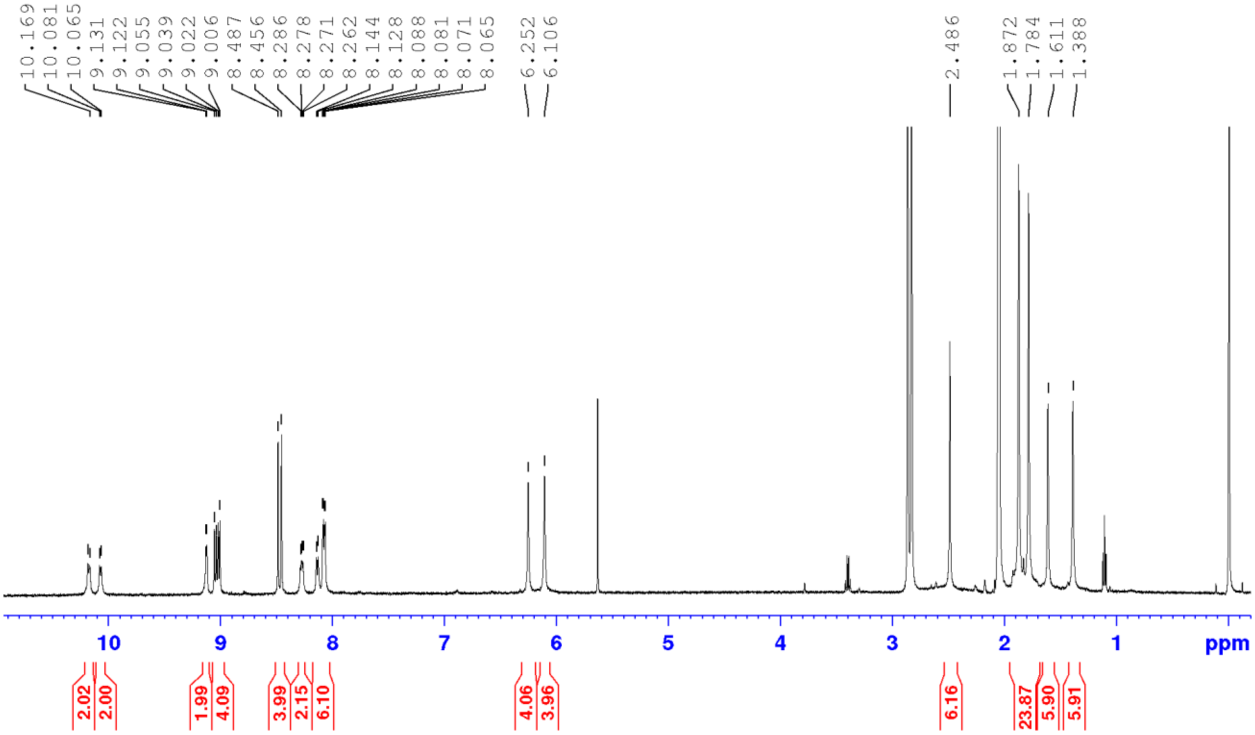

Figure S30. ^{1}H NMR of **CuH_2-$CuMe_2$** in acetone-d_6.

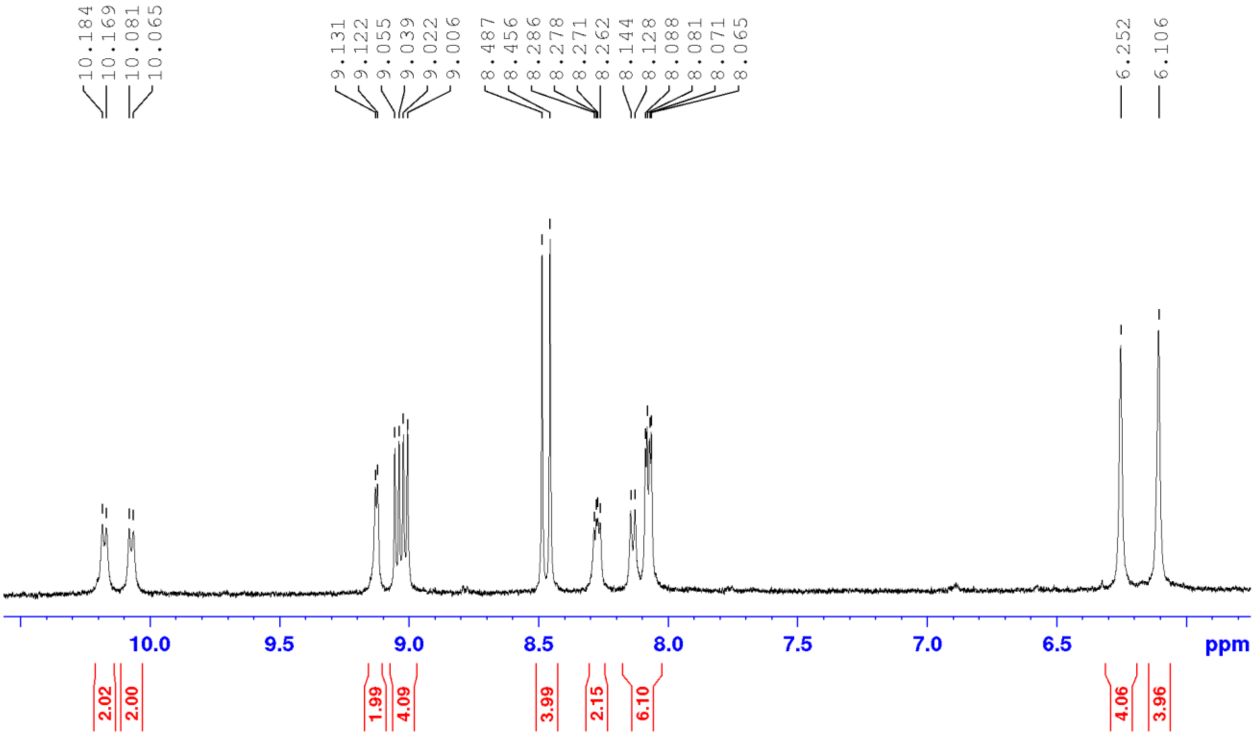

Figure S31. Aromatic region of ^{1}H NMR spectrum of **CuH_2-$CuMe_2$** in acetone-d_6.

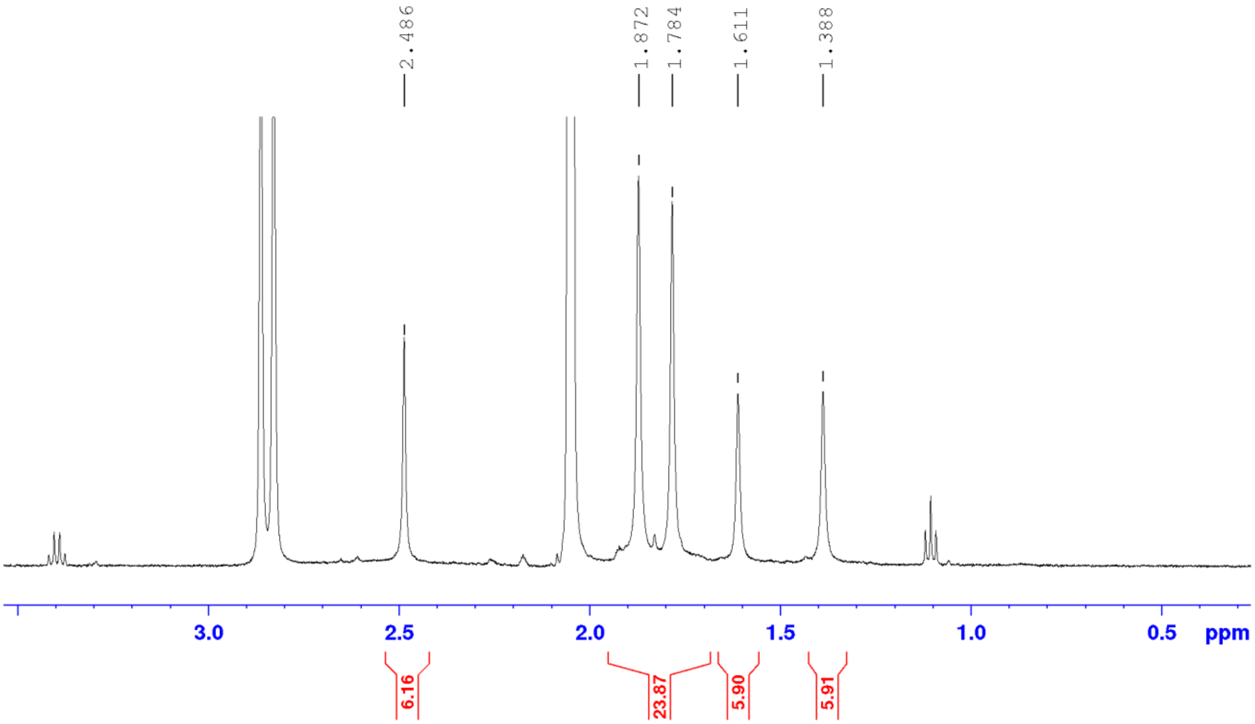

Figure S32. Aliphatic region of ^{1}H NMR spectrum of **CuH_2-$CuMe_2$** in acetone-d_6.

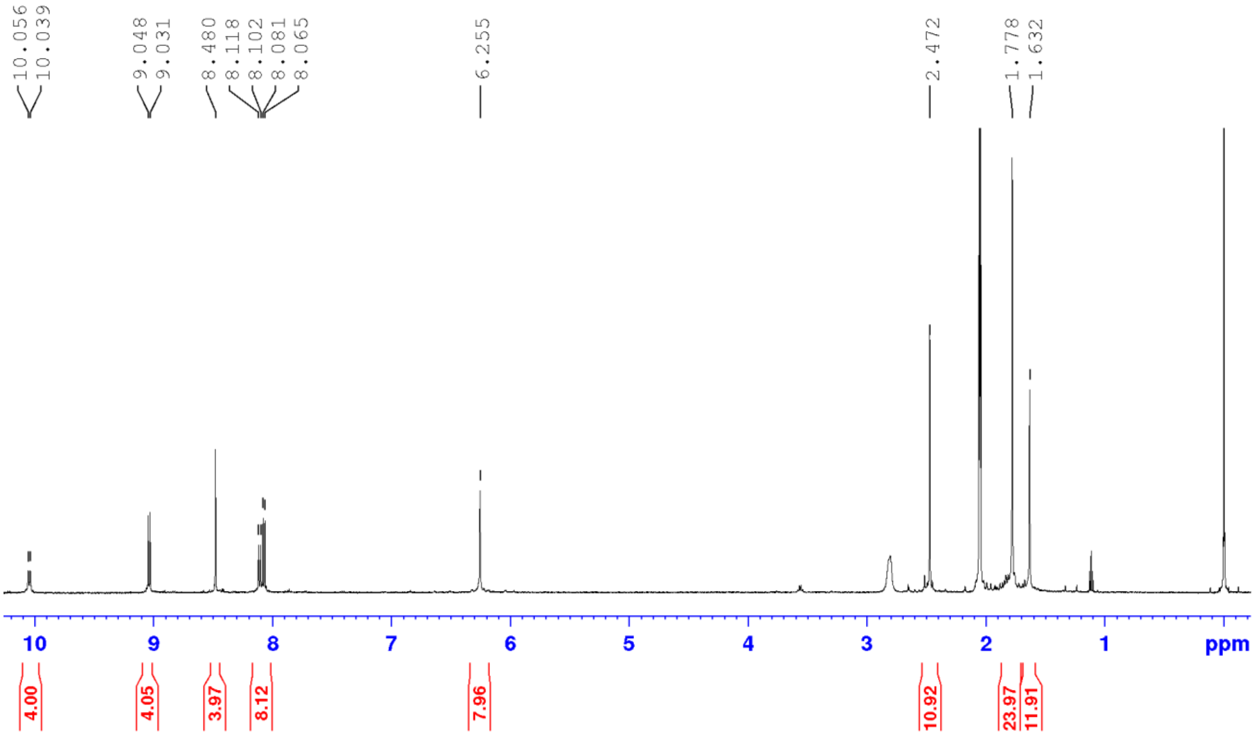

Figure S33. ^{1}H NMR of **$CuMe_2$-$CuMe_2$** in acetone-d_6.

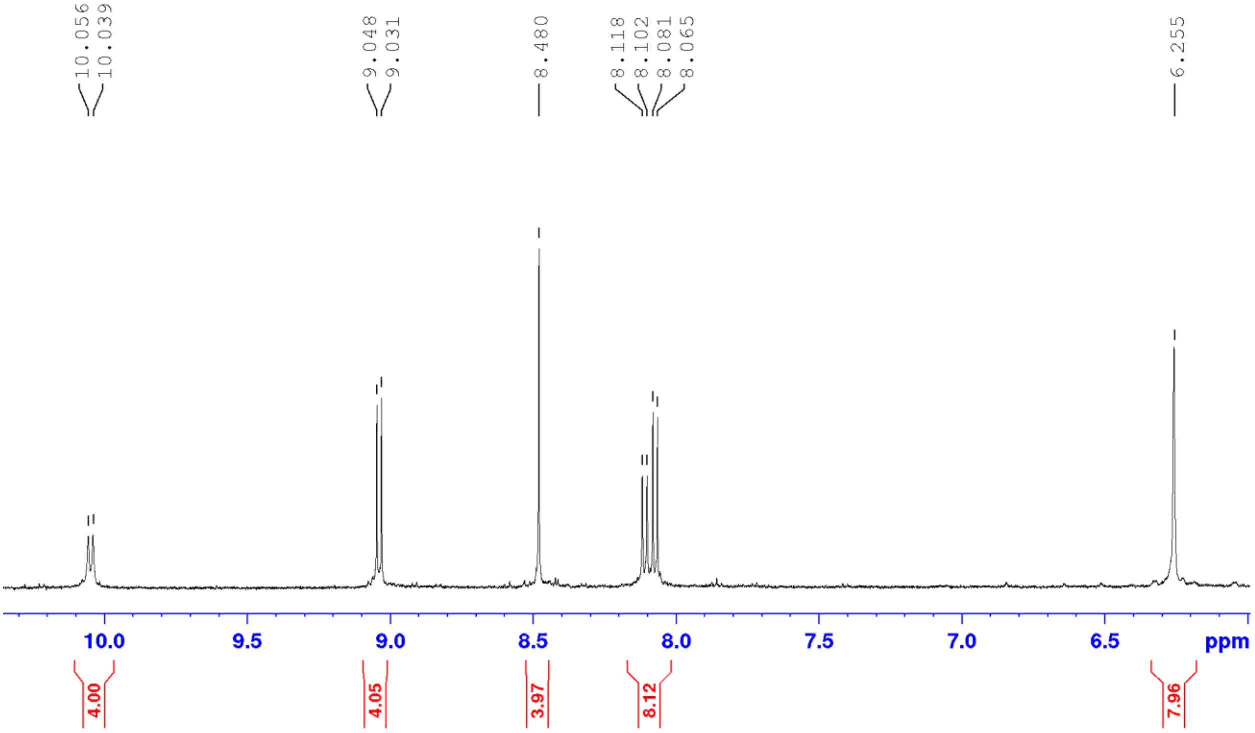

Figure S34. Aromatic region of ^{1}H NMR spectrum of **$CuMe_2$-$CuMe_2$** in acetone-d_6.

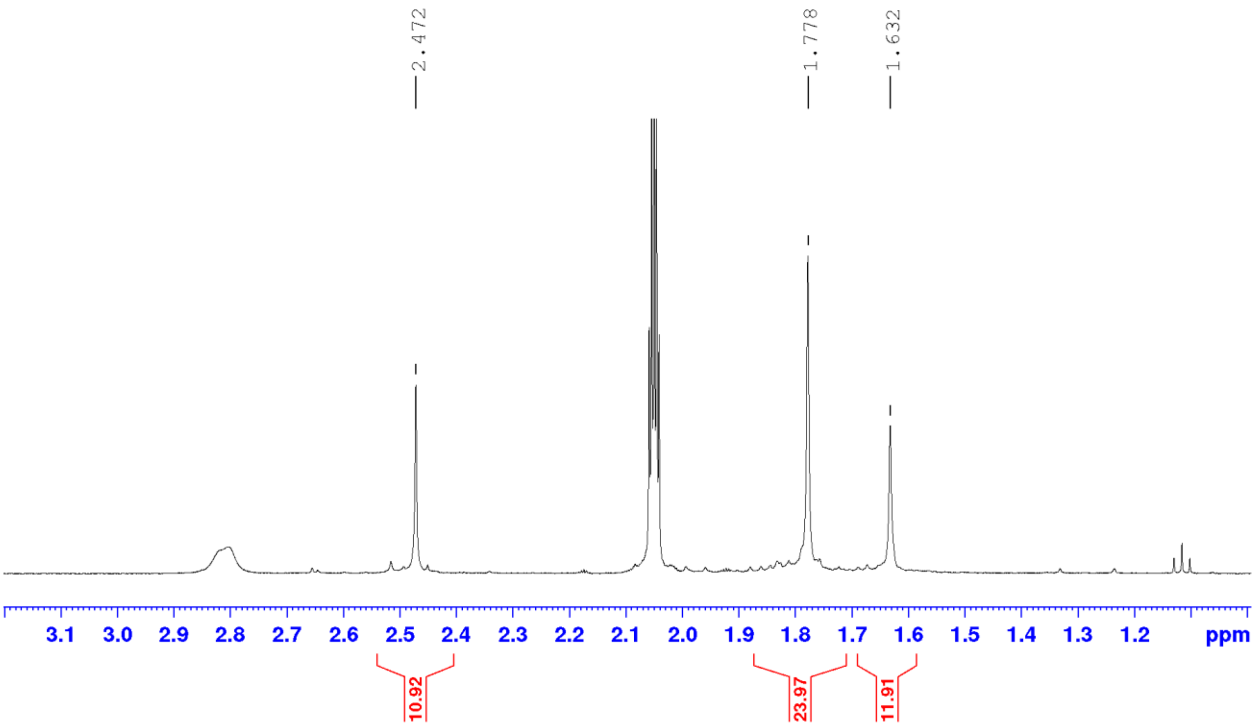

Figure S35. Aliphatic region of ^{1}H NMR spectrum of **$CuMe_2$-$CuMe_2$** in acetone-d_6.

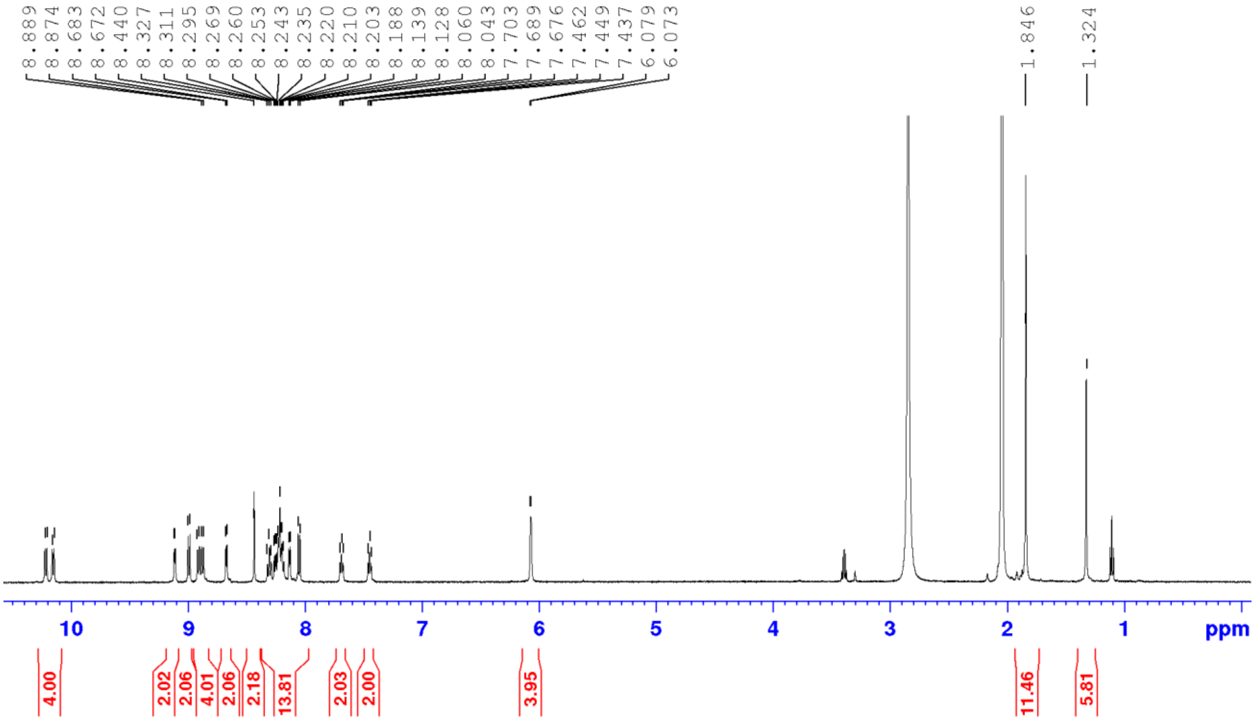

Figure S36. ^{1}H NMR of **CuH_2-RuH_2** in acetone-d_6.

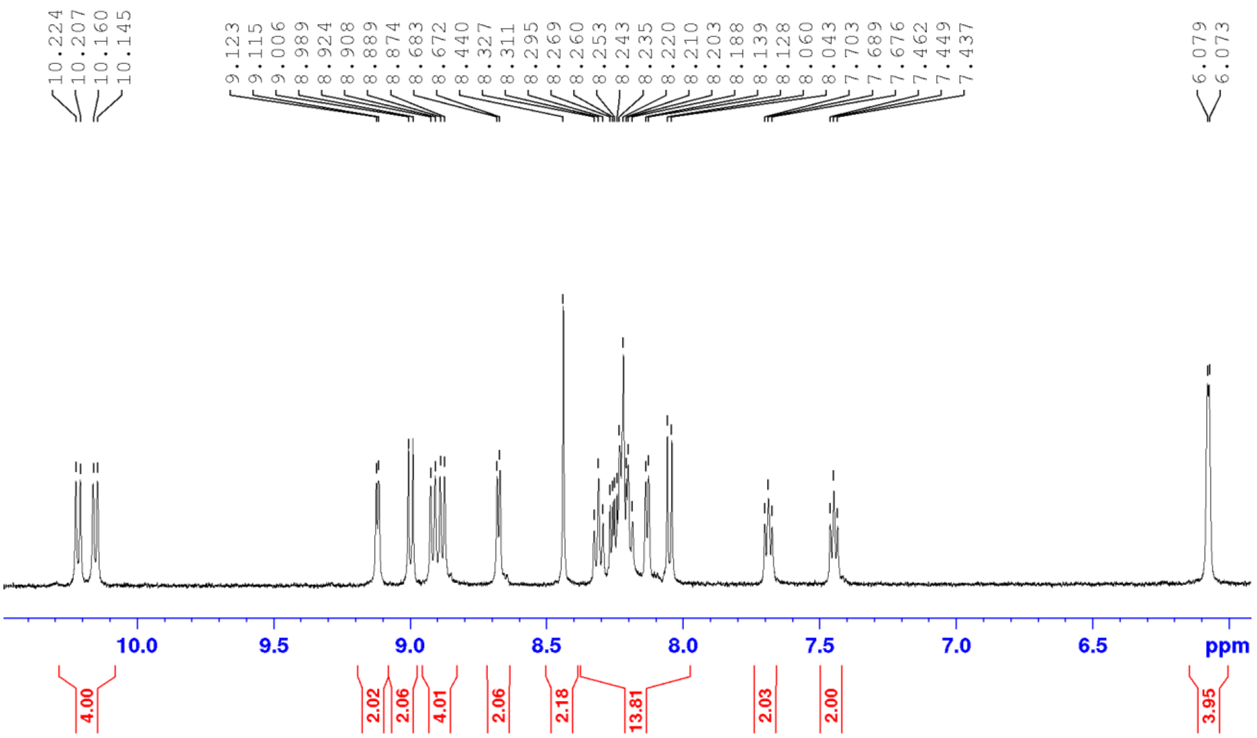

Figure S37. Aromatic region of ^{1}H NMR spectrum of **CuH_2-RuH_2** in acetone-d_6.

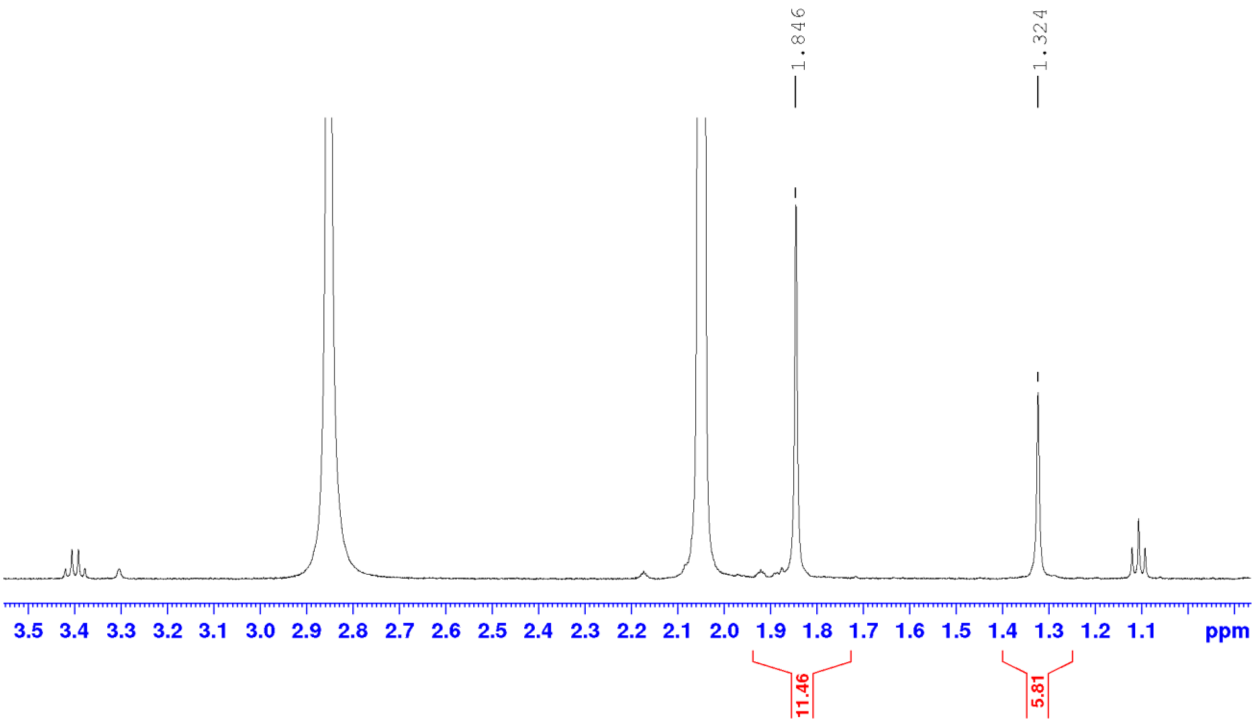

Figure S38. Aliphatic region of ^{1}H NMR spectrum of **CuH_2-RuH_2** in acetone-d_6.

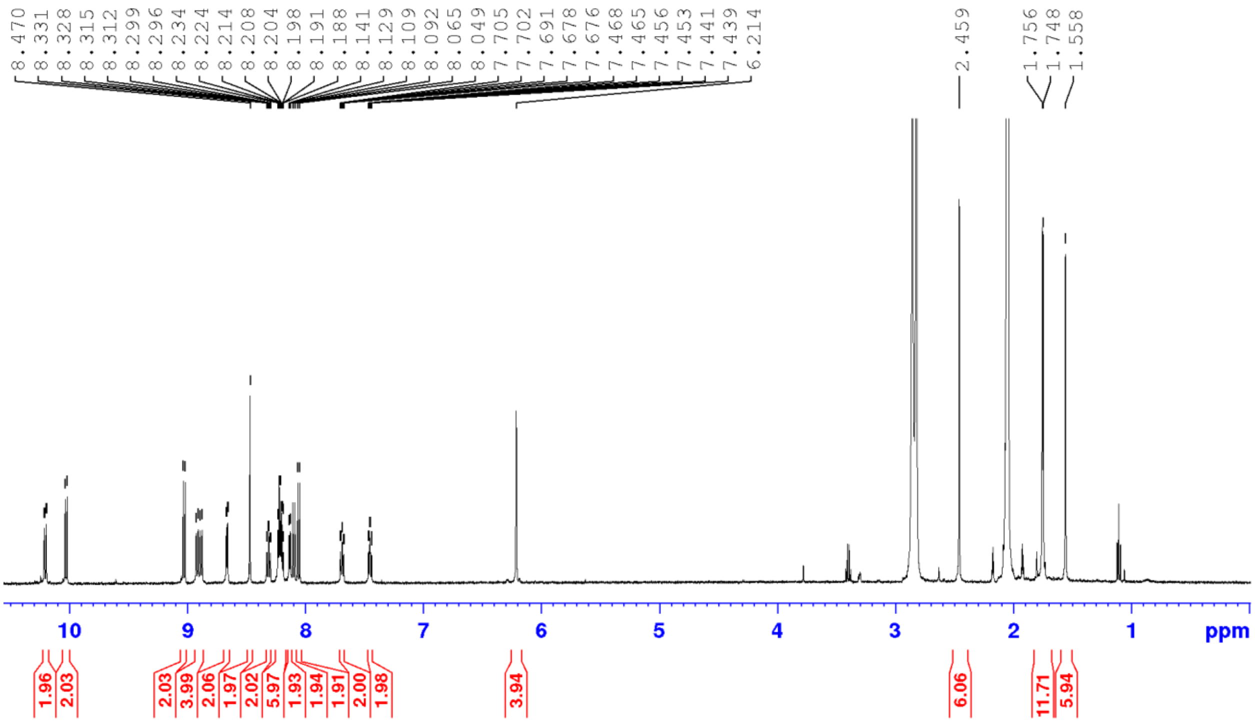

Figure S39. ^{1}H NMR of **CuMe$_2$-RuH$_2$** in acetone-d_6.

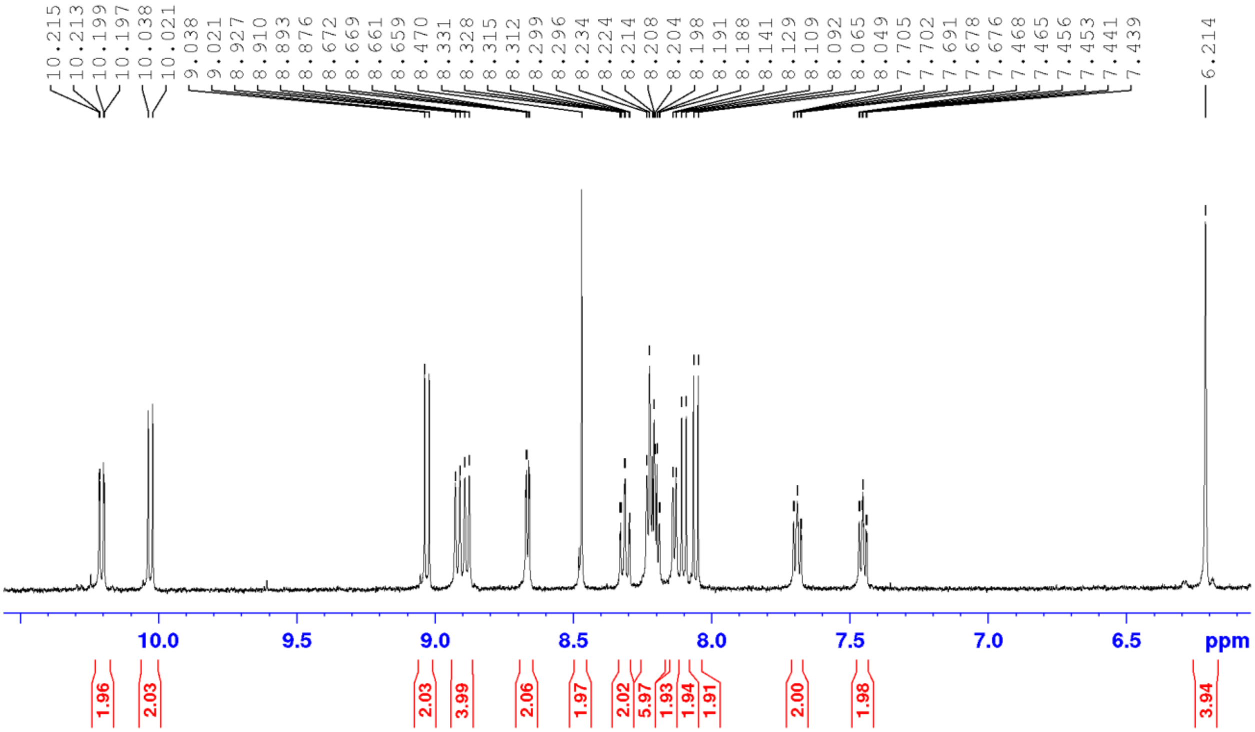

Figure S40. Aromatic region of ^{1}H NMR spectrum of **CuMe$_2$-RuH$_2$** in acetone-d_6.

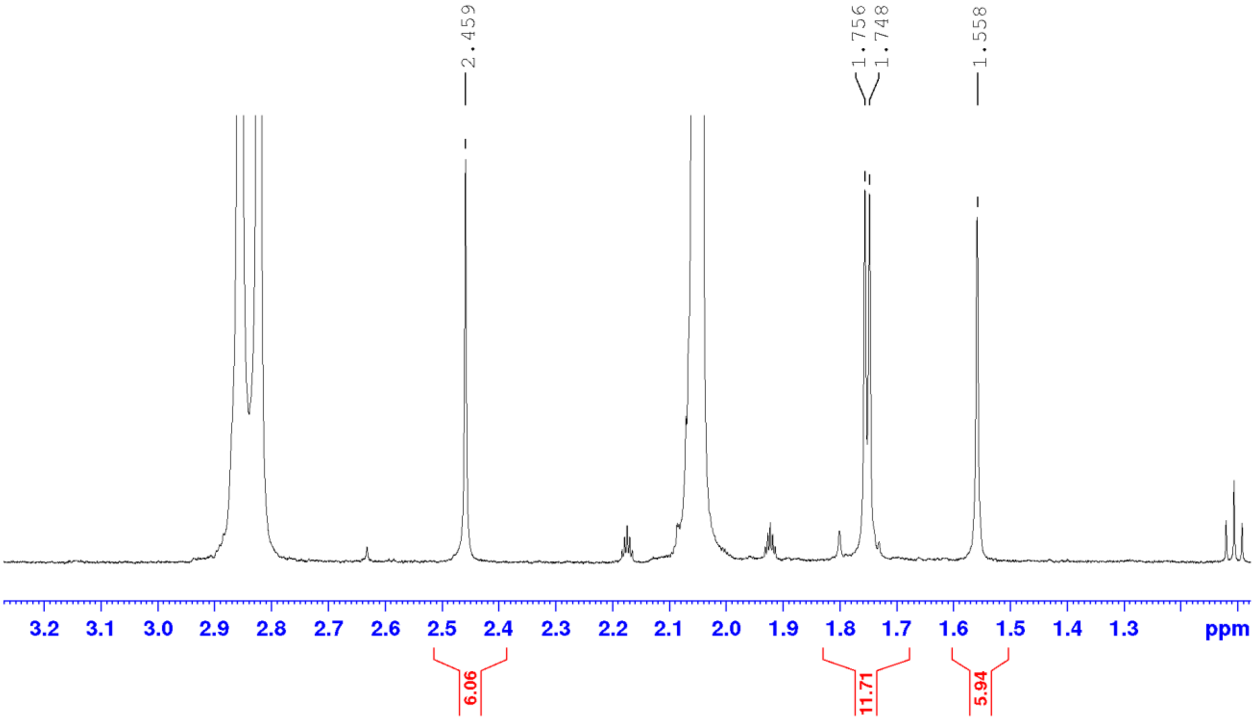

Figure S41. Aliphatic region of ^{1}H NMR spectrum of **$CuMe_2$-RuH_2** in acetone-d_6.

4. Single crystal X-ray diffraction

Crystals of **CuH_2-RuH_2** were mounted on glass fibers using a heavy oil. Full spheres of data were collected at 100 K on a Bruker Apex II diffractometer using Mo Kα radiation. The data were corrected for absorption using SADABS,[7] and the structure was solved using direct methods (SHELXS).[8] Structure refinement was carried out using SHELXL software. Hydrogen atoms were assigned to idealized positions and allowed to refine using a riding model. The data were then corrected for absorption using the program TWINABS. The crystal structure of **CuH_2-RuH_2** was deposited with the Cambridge Crystallographic Data Centre as structure CCDC 1561879.

Table S1. Crystallographic data for **CuH_2-RuH_2**.

Compound	**CuH_2-RuH_2**
Formula	$C_{164.05}H_{143.07}Cu_2F_{36}N_{29.98}O_{2.48}P_6Ru_2$
Mw (g mol^{-1})	3773.04
Lattice Type	Monoclinic
Space Group	P-1
a (Å)	12.383(4)
b(Å)	18.490(7)
c(Å)	19.983(7)
α/β/γ (°)	69.381(5) / 79.742(5) / 88.826(5)
V (Å^3)	4209(3)
Z	1
ρ_{calc} (g cm^{-1})	1.488
T (K)[a]	100
λ (Å) [Mo kα]	0.71073
μ (mm^{-1})	0.584
S (GOF)	0.994
R(F_o), wR(F_o^2)	0.051, 0.136

Table S2. Selected bond lengths and angles for **CuH_2-RuH_2**.

Selected bond lengths			
Cu1-N5	2.017(3)	Ru1-N1	2.053(3)
Cu1-N6	2.024(6)	Ru1-N2	2.060(3)
Cu1-N6B	2.06(2)	Ru1-N9	2.051(3)
Cu1-N7	2.001(3)	Ru1-N10	2.043(3)
Cu1-N7B	2.001(3)	Ru1-N11	2.066(3)
Cu1-N8	2.047(4)	Ru1-N12	2.062(4)
Ru1-Cu1	12.642		
Selected bond angles			
N2-Cu1-N1	138.52(4)	N5-Cu1-N6B	84.5(6)
N2-Cu1-N4	132.68(4)	N8-Cu1-N6B	111.5(6)
N1-Cu1-N4	81.81(3)	N10-Ru1-N9	79.70(14)
N2-Cu1-N3	82.64(4)	N10-Ru1-N1	91.57(13)
N1-Cu1-N3	109.45(3)	N9-Ru1-N1	96.44(13)
N4-Cu1-N3	109.84(4)	N10-Ru1-N2	96.32(14)
N7B-Cu1-N5	134.14(14)	N9-Ru1-N2	174.34(13)
N7-Cu1-N5	134.14(14)	N1-Ru1-N2	79.57(13)
N7-Cu1-N6	122.76(19)	N10-Ru1-N12	174.80(14)
N5-Cu1-N6	81.79(19)	N9-Ru1-N12	99.54(14)
N7B-Cu1-N8	82.34(14)	N1-Ru1-N12	93.63(13)
N7-Cu1-N8	82.34(14)	N2-Ru1-N12	84.79(14)
N5-Cu1-N8	117.40(14)	N10-Ru1-N11	95.47(14)
N6-Cu1-N8	124.26(18)	N9-Ru1-N11	86.65(13)
N7B-Cu1-N6B	128.7(6)	N1-Ru1-N11	172.73(13)
N5-Cu1-N6B	84.5(6)	N2-Ru1-N11	97.79(13)

5. Steady-state spectroscopic characterization

UV-Vis absorption measurements were performed on a Beckman-Coulter DU800 spectrophotometer. Steady state emission spectra were measured on a Quantamaster spectrophotometer from Photon Technology International; each sample was dissolved in spectrophotometric grade acetonitrile with optical density ≤0.3 at the excitation wavelength and thoroughly de-aerated with N_2.

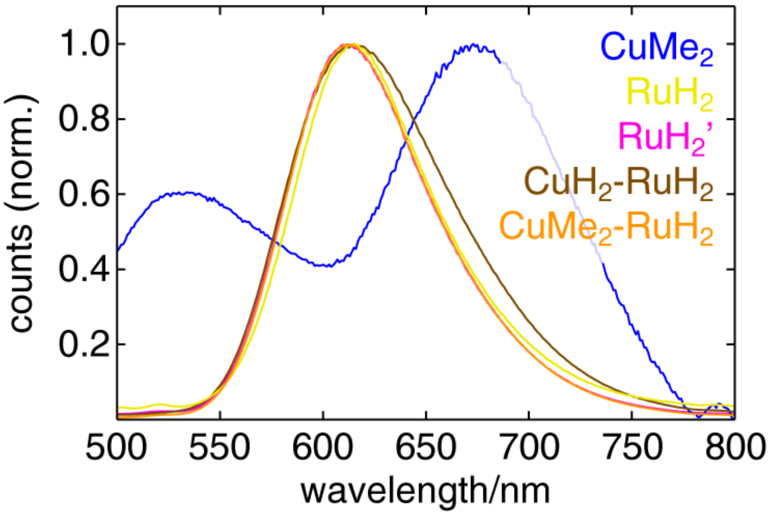

Figure S42. Normalized room temperature emission spectra of mononuclear and heterodinuclear complexes in CH_3CN following MLCT excitation at 460 nm (**CuMe₂**) or 450 nm (all others). **CuH₂** and all three homodinuclear copper complexes studied in this work are non-emissive under these conditions.

6. Cyclic voltammetry

Cyclic voltammetry was conducted using a standard three-electrode cell on a BioAnalytical Systems (BAS 100B) potentiostat and cell stand with a 3mm-diameter glassy carbon working electrode, a Pt wire auxiliary electrode, and a pseudo Ag/AgCl reference electrode. Each solution in anhydrous acetonitrile was purged with N_2 prior to measurement and subsequently maintained under a blanket of N_2. Tetrabutylammonium hexafluorophosphate (0.1M) was used as the supporting electrolyte. Ferrocene (purified by sublimation) was added as an internal standard and redox potentials were referenced to the ferrocene/ferrocenium couple (0.40V vs. SCE (acetonitrile)). All scans were performed at 100 mV/s.

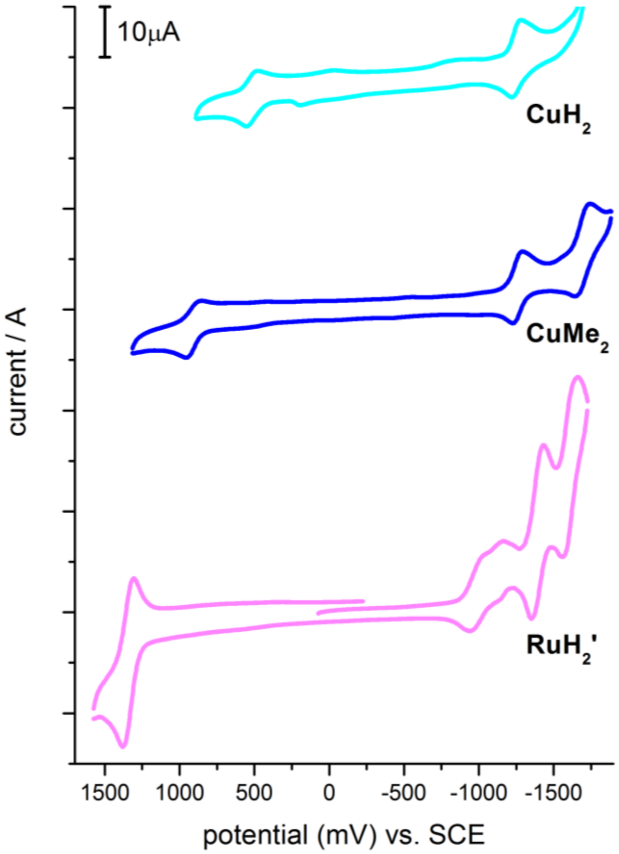

Figure S43. CV of mononuclear complexes in CH_3CN. 1mM complex, 0.1M $TBAPF_6$ / CH_3CN; glassy carbon working electrode, Pt wire auxiliary, pseudo Ag/AgCl reference; scans referenced to SCE using fc/fc^+ as internal standard; scans performed at 100mV/s.

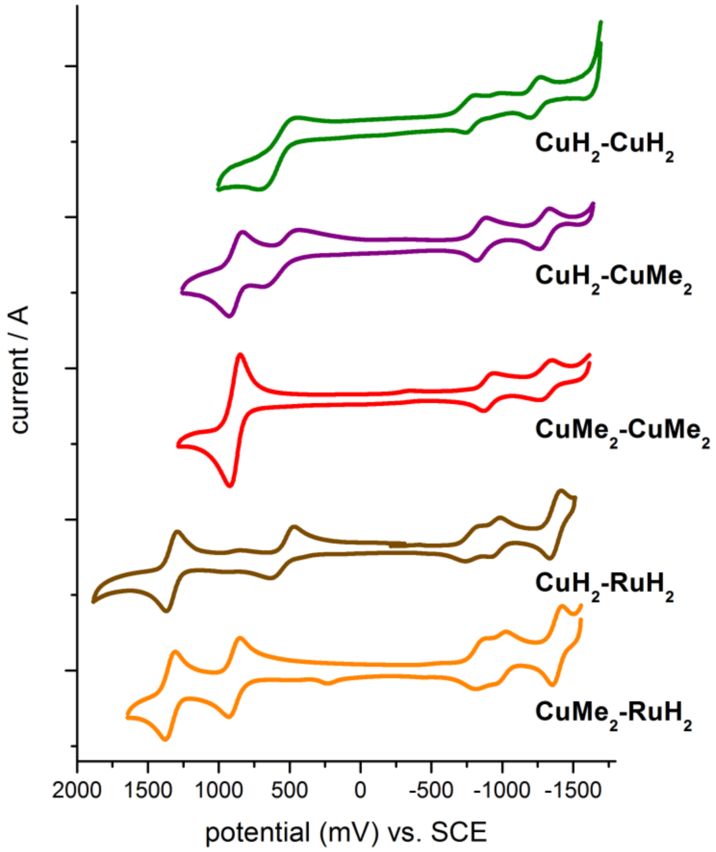

Figure S44. CV of dinuclear complexes in CH_3CN. 1mM complex, 0.1M $TBAPF_6$ / CH_3CN; glassy carbon working electrode, Pt wire auxiliary, pseudo Ag/AgCl reference; scans referenced to SCE using fc/fc^+ as internal standard; scans performed at 100mV/s.

7. Electronic structure calculations

All DFT calculations were carried out using Gaussian 09, revision A.01,[9] software installed on the Blues or Fusion clusters at Argonne National Laboratory. Geometry optimizations were carried out using the B3LYP functional[10] (spin unrestricted for paramagnetic states), in combination with a 6-31G(d) basis set[11–13] for all atoms except Ru, for which the MWB28 pseudopotential[14] was used. Frequency calculations were carried out to ensure structures represented energetic minima. Single point energy calculations were also carried out using the B3LYP functional, but with a higher level split basis set (6-311+G(d) for Cu and N, 6-31G(d) for C and H, and MWB28 for Ru). Orbital surfaces were generated using the β-LUMO program,[15] and gas phase TDDFT calculations were visualized using the SWizard program, revision 4.6,[16,17] using band-shapes with half-widths of 3000 cm^{-1}. Solvation (acetonitrile) was included using the polarized continuum model (PCM).[18] Inner sphere reorganization energies (λ_i) upon single electron oxidation were calculated as $\lambda_i = \lambda_{ox} + \lambda_{red}$, ($\lambda_{ox}$ = E(ox)(red) – E(ox)(ox) and λ_{red} = E(red)(ox) – E(red)(red), where E(1)(2) refers to the calculated energy of a molecule in the geometry of (2) and the oxidation state of (1)).[19,20]

Table S3. TD-DFT calculated transitions for **CuH_2**.[a]

State	Energy (nm)	Energy (cm^{-1})	*f*	Contributions	Assignment
1	535	18,700	0.0119	224 → 225 (39%)<br>224 → 226 (27%)<br>223 → 226 (21%)	Cu(d_1) → tpphz(N)<br>Cu(d_1) → tpphz(phen)<br>Cu(d_2) → tpphz(phen)
3	509	19,600	0.0211	224 → 228 (41%)<br>223 → 225 (40%)	Cu(d_1) → phen<br>Cu(d_2) → tpphz(N)
5	469	21,300	0.2654	223 → 228 (35%)<br>224 → 226 (26%)<br>223 → 226 (23%)	Cu(d_2) → phen<br>Cu(d_1) → tpphz(phen)<br>Cu(d_2) → tpphz(phen)
9	422	23,700	0.0318	223 → 229 (88%)	Cu(d_2) → phen
15	384	26,000	0.4052	221 → 225 (92%)	IL

[a]Calculated transitions with oscillator strengths greater than 0.01 are listed. B3LYP; 6-311+G(d) (Cu, N) and 6-31G(d) (C, H); PCM = CH_3CN. 40 total states considered.

Table S4. TD-DFT calculated transitions for **$CuMe_2$**.[b]

State	Energy (nm)	Energy (cm^{-1})	*f*	Contributions	Assignment
1	549	18,200	0.0318	232 → 233 (72%)	Cu(d_1) → tpphz(N)
2	526	19,000	0.0162	232 → 234 (69%)	Cu(d_1) → phen
4	480	20,800	0.0716	231 → 233 (76%)	Cu(d_2) → tpphz(N)
5	466	21,500	0.0136	232 → 237 (85%)	Cu(d_1) → phen
6	455	22,000	0.1277	231 → 235 (54%)	Cu(d_2) → tpphz(phen)
7	450	22,200	0.0125	232 → 236 (85%)	Cu(d_1) → tpphz
8	422	23,700	0.0125	231 → 234 (34%)<br>231 → 237 (26%)	Cu(d_2) → phen<br>Cu(d_2) → phen
12	393	25,400	0.0106	230 → 234 (67%)	Cu(d_3) → phen
13	393	25,400	0.0110	228 → 233 (94%)	IL
14	385	26,000	0.4500	229 → 233 (87%)	IL

[b]Calculated transitions with oscillator strengths greater than 0.01 are listed. B3LYP; 6-311+G(d) (Cu, N) and 6-31G(d) (C, H); PCM = CH_3CN. 40 total states considered.

Table S5. TD-DFT calculated transitions for **CuH_2-CuH_2**.[c]

State	Energy (nm)	Energy (cm^{-1})	*f*	Contributions	Assignment
2	579	17,300	0.0154	348 → 350 (95%)	Cu(2)(d_1) → tpphz(N)
4	542	18,400	0.0190	349 → 352 (26%)<br>349 → 353 (26%)	Cu(1)(d_1) → tpphz(phen)<br>Cu(1)(d_1) → tpphz(phen)
5	534	18,700	0.0382	346 → 350 (79%)	Cu(1)(d_2) → tpphz(N)
11	478	20,900	0.0140	348 → 351 (89%)	Cu(2)(d_1) → tpphz
12	475	21,100	0.3687	348 → 352 (34%)<br>347 → 354 (29%)<br>348 → 353 (28%)	Cu(2)(d_1) → tpphz(phen)<br>Cu(2)(d_2) → phen<br>Cu(2)(d_1) → tpphz(phen)
13	462	21,700	0.1312	346 → 355 (33%)<br>346 → 352 (23%)<br>346 → 353 (21%)	Cu(1)(d_2) → phen<br>Cu(1)(d_2) → tpphz(phen)<br>Cu(1)(d_2) → tpphz(phen)
14	456	21,900	0.0127	349 → 356 (95%)	Cu(1)(d_1) → phen
20	417	24,000	0.0165	347 → 354 (61%)	Cu(2)(d_2) → phen
21	417	24,000	0.0336	347 → 357 (94%)	Cu(2)(d_2) → phen
22	412	24,300	0.0151	346 → 355 (47%)	Cu(1)(d_2) → phen
24	404	24,700	0.0228	346 → 356 (84%)	Cu(1)(d_2) → phen

[c]Calculated transitions with oscillator strengths greater than 0.01 are listed. B3LYP; 6-311+G(d) (Cu, N) and 6-31G(d) (C, H); PCM = CH_3CN. 25 total states considered.

Table S6. TD-DFT calculated transitions for **CuH_2-RuH_2**.[d]

State	Energy (nm)	Energy (cm^{-1})	*f*	Contributions	Assignment
8	478	20,900	0.3523	310 → 314 (41%)<br>313 → 319 (24%)	Ru(d_2) → tpphz(N)<br>Cu(d_1) → tpphz(phen)
10	472	21,200	0.0243	310 → 314 (51%);<br>313 → 319 (21%)	Ru(d_2) → tpphz(N)<br>Cu(d_1) → tpphz(phen)
22	434	23,100	0.0226	310 → 317 (38%)<br>310 → 316 (27%)<br>311 → 315 (23%)	Ru(d_2) → tpphz(phen)<br>Ru(d_2) → tpphz(phen)<br>Ru(d_1) → tpphz
24	421	23,800	0.1390	309 → 318 (27%)<br>310 → 316 (24%)	Ru(d_3) → phen<br>Ru(d_2) → tpphz(phen)
25	420	23,800	0.0230	312 → 317 (81%)	Cu(d_2) → phen
26	417	24,000	0.1271	309 → 317 (54%)<br>310 → 318 (35%)	Cu(d_2) → phen<br>Ru(d_2) → tpphz(phen)
27	416	24,000	0.0173	312 → 320 (54%)	Cu(d_2) → phen
30	412	24,300	0.0342	312 → 321 (90%)	Cu(d_2) → phen

[d]Calculated transitions with oscillator strengths greater than 0.01 are listed. B3LYP; 6-311+G(d) (Cu, N), 6-31G(d) (C, H), MWB28 (Ru); PCM = CH_3CN. 30 total states considered.

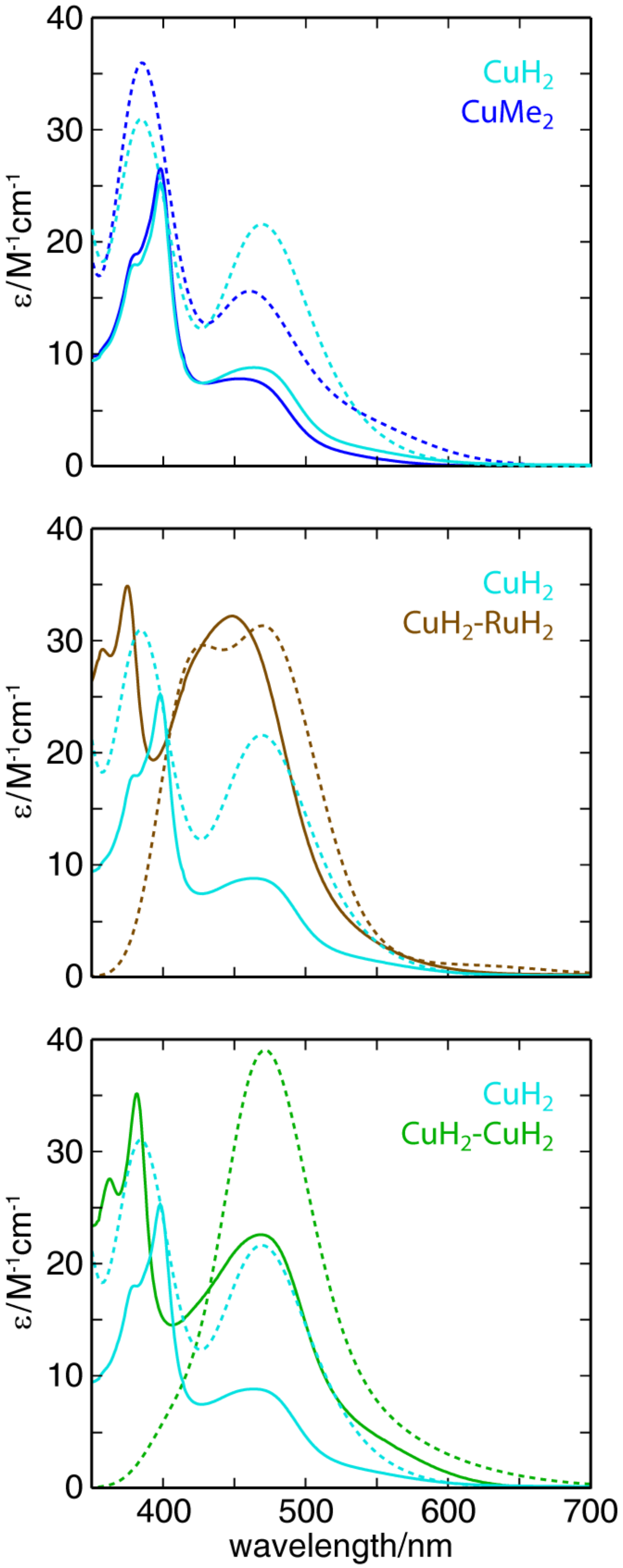

Figure S45. Comparison between experimental (solid lines) and TD-DFT calculated (dashed lines) absorption spectra of **CuH_2** (cyan) and **$CuMe_2$** (blue), **CuH_2-RuH_2** (brown), and **CuH_2-CuH_2** (green).

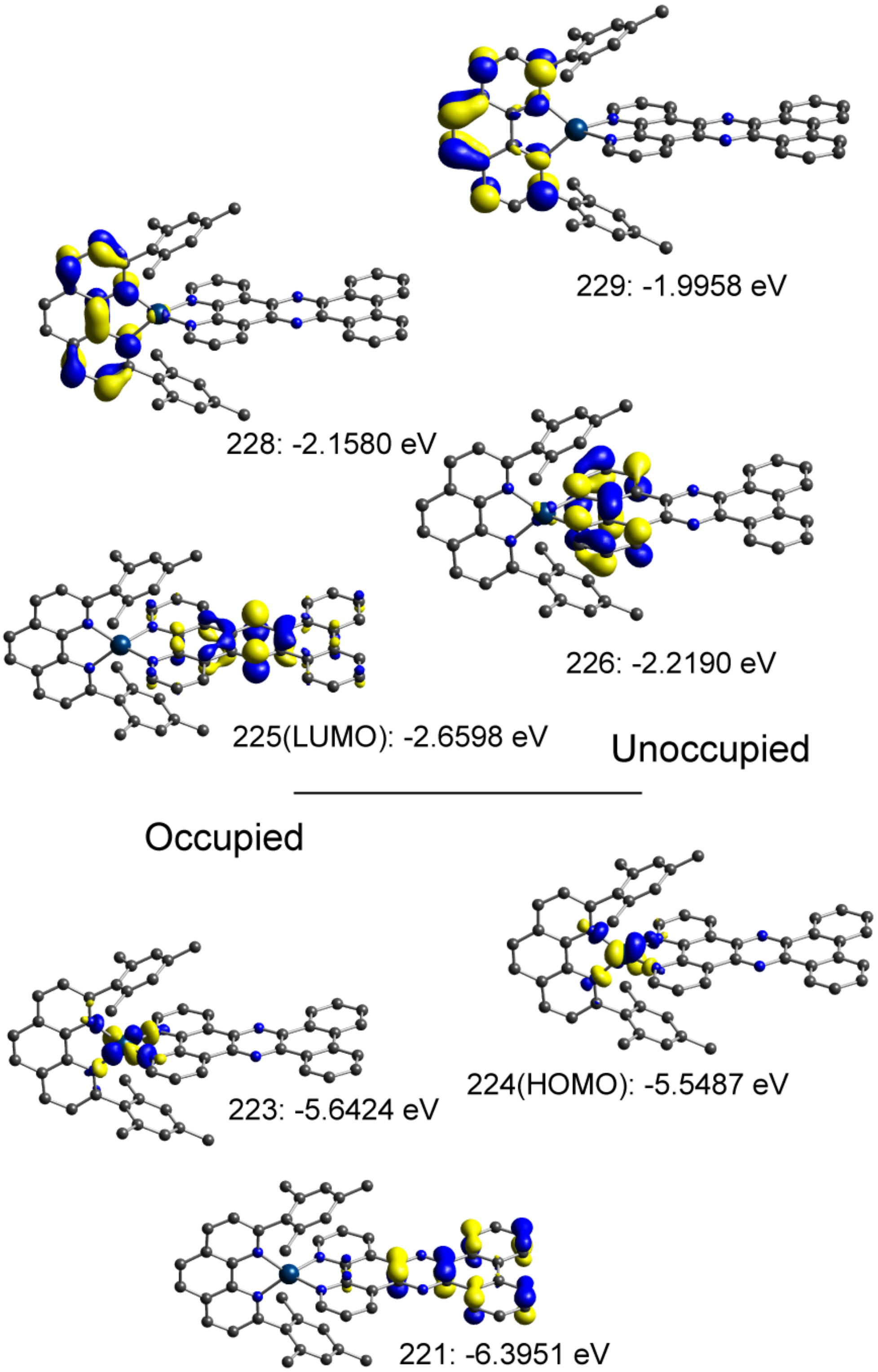

Figure S46. Donor and acceptor molecular orbitals for $\mathbf{CuH_2}$.

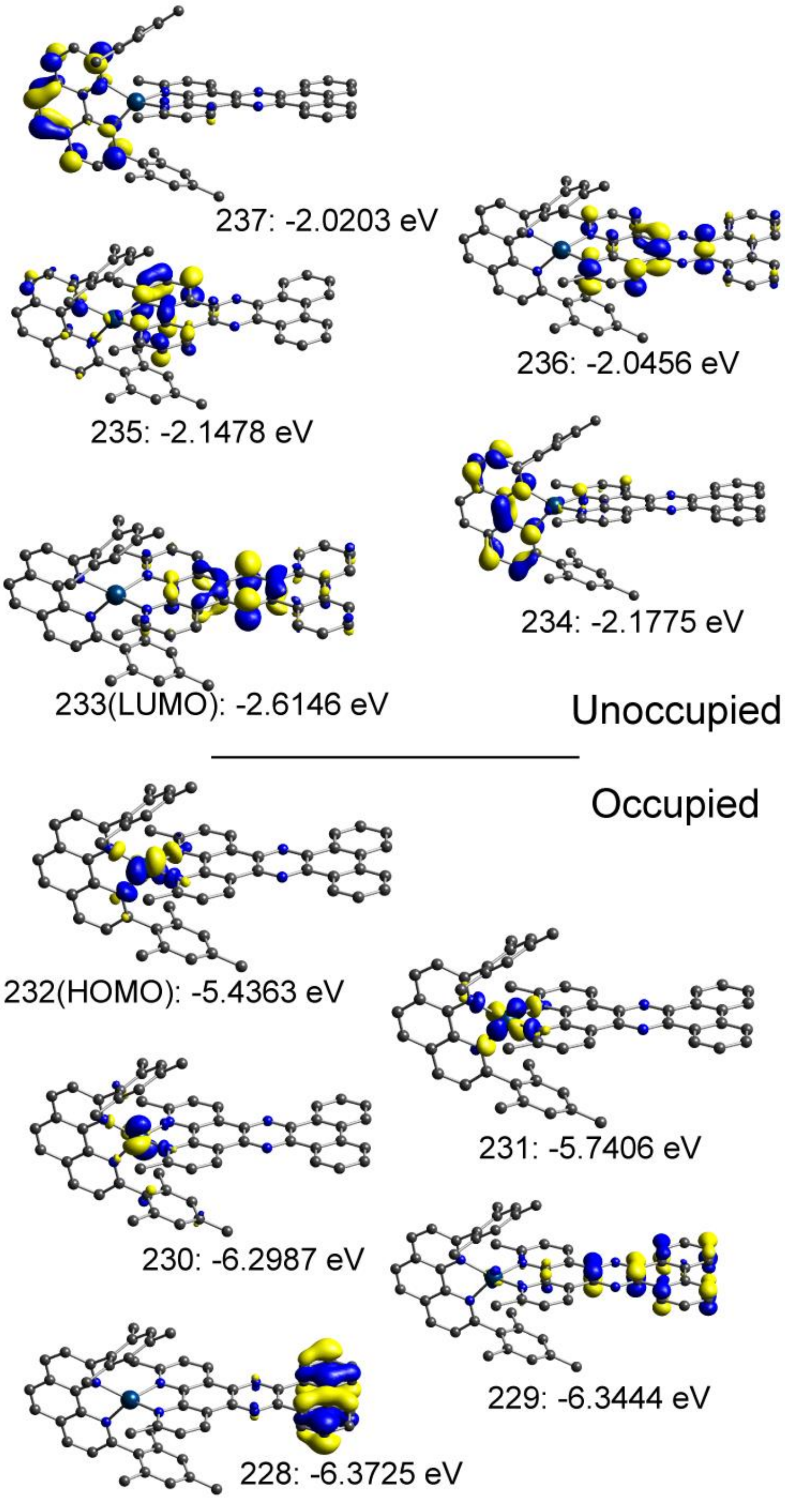

Figure S47. Donor and acceptor molecular orbitals for **$CuMe_2$**.

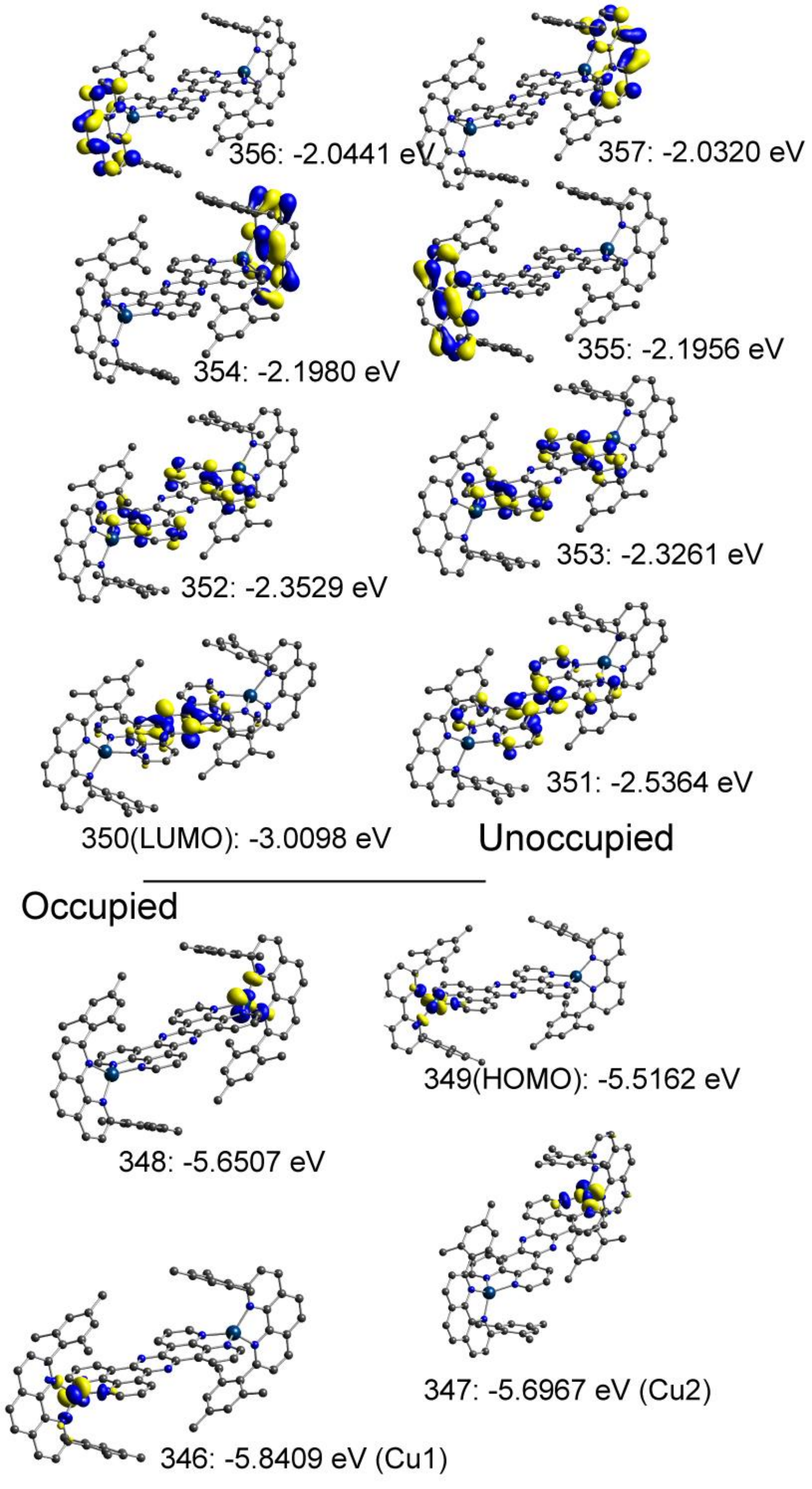

Figure S48. Donor and acceptor molecular orbitals for **CuH_2-CuH_2**.

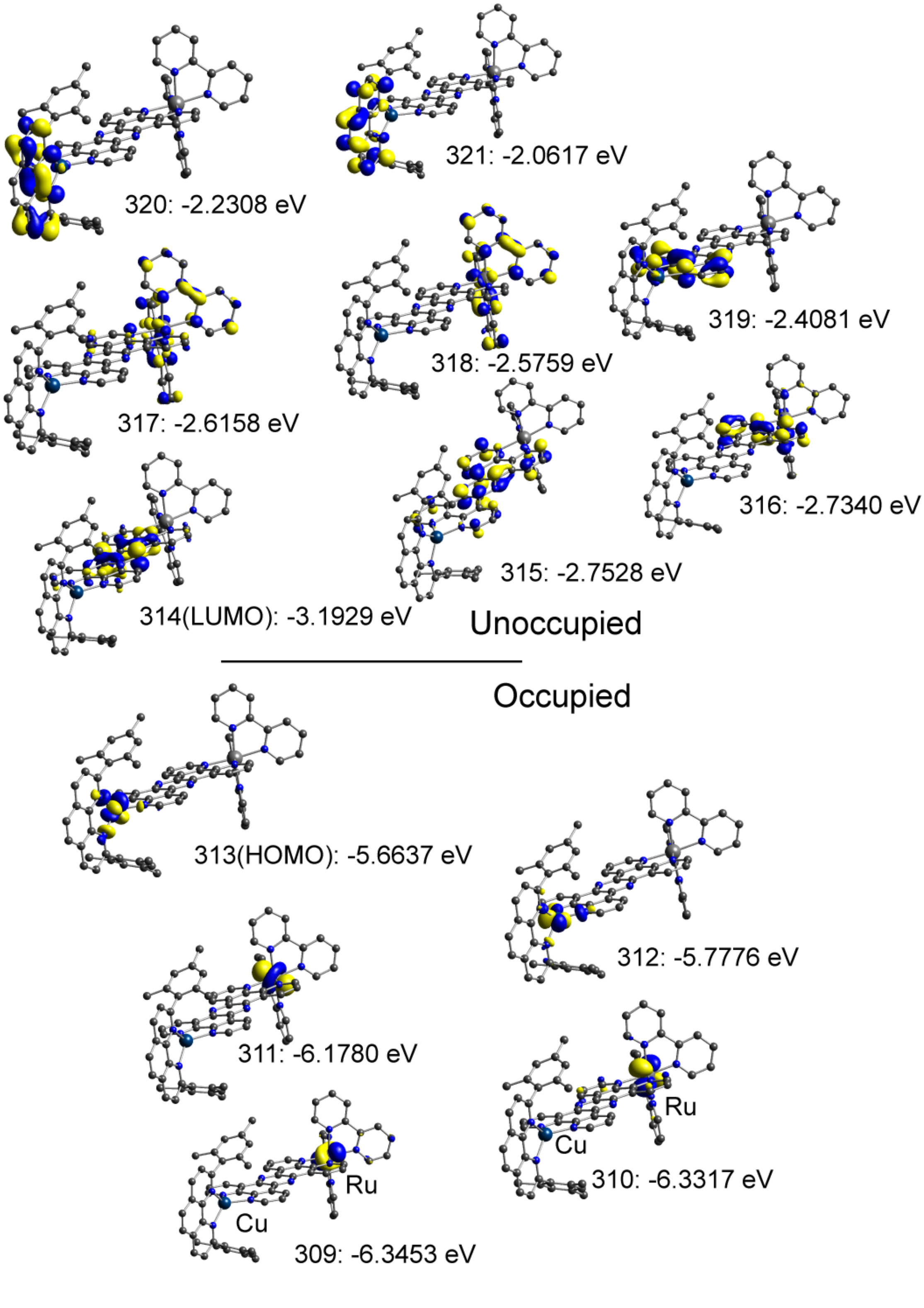

Figure S49. Donor and acceptor molecular orbitals for $\mathbf{CuH_2}$-$\mathbf{RuH_2}$.

8. Femtosecond and nanosecond optical transient absorption

Ultrafast transient absorption measurements were performed using a commercial regeneratively amplified Ti:Sapphire laser (Spectra-Physics) and an automated data acquisition system (Ultrafast Systems, Helios). A 1.0 kHz pulse train of 100 fs, 2.9 mJ pulses centered at 830 nm was produced by a Spitfire Pro amplifier seeded by a Mai Tai oscillator and pumped by an Empower frequency-doubled, diode-pumped Nd:YLF Q-switched laser. One third of the total power was used for the transient absorption experiments. The beam was split, with 90% of the beam used for the pump and 10% used for the probe. The pump was focused into a Type 1 second harmonic generation (SHG) BBO crystal, and the 415 nm component was isolated using a dichroic filter. The pump beam was then passed through a depolarizer, attenuated to the desired power using an absorptive neutral density filter, and chopped at 500 Hz. The probe beam was delayed relative to the pump with a retroreflector mounted on a motorized delay stage and focused into a sapphire plate to generate a white light continuum, which was passed through a high pass filter to give a continuum spanning from 430 to 750 nm. The pump and probe beams were focused and overlapped at the sample position with beam radii ($1/e^2$) of 500 and 85 µm, respectively, and the transmitted probe was focused into a fiber-coupled multichannel spectrometer and CMOS sensor. Unless otherwise stated, all OTA measurements were performed with a pump pulse energy of 0.25 µJ (64 $\mu J/cm^2$ fluence).

Optical transient absorption spectroscopy on the ns-µs timescale was performed using a home-built apparatus. A tunable narrow band laser (Ekspla PL2210 Nd:YAG and OPG with a pulse duration of 50 ps) was used as the pump excitation and also served as the master 1 kHz clock. The pump repetition rate was set at 500 Hz via internal control of the Q-switch. A 1 kHz broadband supercontinuum fiber laser (Leukos STM with a pulse duration of 700 ps) was used as the probe. Time delays were achieved using a delay generator (Stanford Research Systems DG535) externally synchronized to the 1 kHz clock from the pump laser. The transmitted probe intensity was spectrally resolved via a spectrometer (Acton Research Corporation Spectra Pro 2150i) and recorded with a line scan camera (Teledyne Dalsa Spyder3 1k). The instrument was controlled using home-written LabVIEW code.

Samples were prepared in anhydrous acetonitrile at an optical density of ~0.45 at 415 nm in a quartz cuvette with a pathlength of 2 mm. The samples were deaerated with N_2 and sealed with Parafilm.

9. Copper and ruthenium K-edge absorption

Transient and steady-state X-ray absorption measurements were performed at beamline 11-ID-D at the Advanced Photon Source (APS, Argonne National Laboratory). The experimental details of transient absorption measurements at this beamline have been reported elsewhere,[21–23] but the experiments reported here differ in two important ways: 1) The APS was operated in hybrid fill mode for the ruthenium K-edge measurements of **$CuMe_2$-RuH_2**; and 2) The optical pump was the second harmonic of a Ti:Sapphire laser. These differences are detailed below.

1. Hybrid fill operating mode. In this continuous top-up mode, the 102 mA ring current of the APS is divided into a 16 mA singlet bunch followed by a train of 56 smaller bunches with an orbit period of 3.68 µs. The rms bunch length of the 16 mA singlet is 50 ps, and the instrument response full-width half-maximum (FWHM) measured at the beamline was 120 ps.

2. Optical pump. The excitation source was a commercial renegeratively amplified Ti:Sapphire laser system (Coherent). A 3.0 kHz pulse train of 1 ps, 4 mJ pulses centered at 800 nm was produced by a Legend Elite Duo amplifier seeded by a Micra-5 oscillator and pumped by an Evolution intracavity-doubled, diode-pumped Nd:YLF Q-switched laser. The 351.926 MHz rf signal from the storage ring was divided by 4, and the oscillator was phase locked to the rf/4 signal using the Synchrolock AP system (Coherent). The 271.5 kHz signal from the storage ring corresponding to the orbit period was used to trigger a digital delay generator (Stanford Research Systems DG535) that subsequently triggered the Q-switch at a repetition rate of 3.0 kHz. Although the amplifier was seeded with transform-limited, 100 fs pulses from the oscillator, the compression following amplification was intentionally detuned to give temporally chirped, 1 ps pulses to increase the excited state fraction of the sample and minimize sample damage from the laser. The 800 nm beam was passed through a λ/2 waveplate, a Type 1 SHG BBO crystal, and a polarizer to give a 400 nm beam with 0.12 mJ/pulse. The pulse power was adjusted by rotating the waveplate before the SHG crystal and polarizer. The beam was focused with a 300 mm lens such that the beam reached the focal point behind the sample and the spot size at the position of the liquid jet was 750 x 600 µm. The X-ray spot size was 500 x 200 µm. The laser and X-ray beams were overlapped at the sample position by aligning through a pinhole. A fast photodiode and 8 GHz oscilloscope (Infinium, Agilent) was then used to set the zero time delay between the singlet X-ray bunch and the laser pulse by adjusting the timing of the Synchrolock.

The X-ray probe pulses were obtained from the storage ring electron bunches using dual inline undulators followed by an actively stabilized dual crystal Si(111) monochromator. An upstream APD detector channel was used for pulse-by-pulse normalization of the fluorescence signal. A nickel (for copper measurements) or molybdenum (for ruthenium measurements) Z-1 filter (3 absorption lengths) and soller slit assembly was mounted between the sample jet and each fluorescence detector to minimize background signal from elastic scattering. The signal of both APD channels was recorded at a 1 GHz sampling rate by a fast analyzer card (Agilent), and the data was processed in real time by software written by Dr. Guy Jennings (APS), which performs background subtraction, pulse shape fitting, and signal averaging.

XANES spectra were acquired with 4 second averaging at each energy point, and all XTA spectra reported are the average of at least 18 scans. The 8 orbits of the laser-synchronized electron bunch arriving before each laser pulse were averaged to give the ground state reference XANES spectrum, and the difference spectra reported are the difference of the raw spectra obtained from the laser-synchronized orbit and the

ground state reference. No normalization or background subtraction was performed on the XANES spectra before taking the difference. The energy axis of each difference spectrum was calibrated before averaging to the edge of a Cu foil reference spectrum measured simultaneously using the transmitted X-ray beam. XTA spectra of all samples were acquired at a pump-probe delay time of 50 ps.

Time scans were acquired with 8 second averaging at each time point, and all time traces reported are the average of at least 16 scans. The time traces correspond again to the difference between the laser-synchronized orbit and the 8 previous orbits of the same bunch. For the Ru K-edge time traces, the same number of laser-off scans were acquired, and the laser-off difference time traces were then subtracted from the laser-on difference time traces. This step was necessary to eliminate any small contributions to the weak Ru K-edge data from electrical noise originating from the firing of the Pockels cell high-speed drivers in the regenerative amplifier that is picked up by the APD amplifiers. The Cu K-edge signal, however, was generally an order of magnitude higher than the Ru K-edge signal, so this step was not performed for the Cu data.

All samples were prepared in anhydrous acetonitrile at a concentration of 2 mmol in the metal being probed (i.e. all mononuclear and heterodinuclear samples were prepared as 2 mmol solutions, while all homodinuclear samples were prepared as 1 mmol solutions). The recirculating sample was continuously purged with dry nitrogen and delivered as a 600-650 μm liquid jet at the sample position.

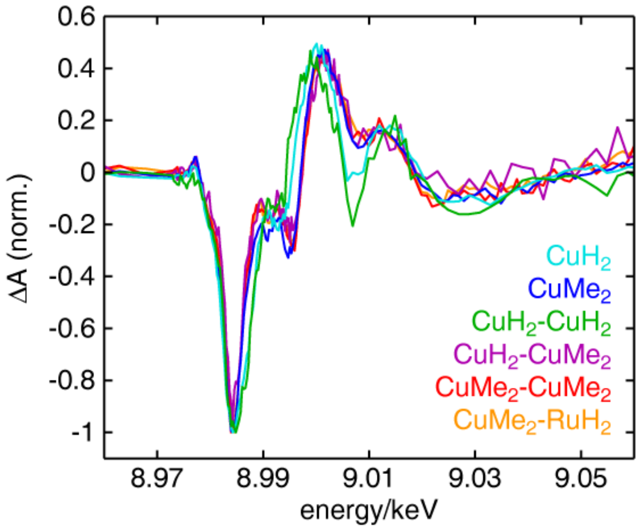

Figure S50. Comparison of Cu K-edge XTA difference spectra of mononuclear, homodinuclear, and heterodinuclear Cu(I) complexes. All spectra were acquired at a delay time of 50 ps following excitation of the MLCT band at 400 nm.

10. Data analysis and fitting

All data analysis was performed using MATLAB R2013a (MathWorks). All fits were obtained using the nonlinear least-squares fitting function lsqcurvefit. Although lower and upper bounds were used to constrain some of the fits to physically reasonable parameter spaces, all fits converged to solutions in which the variables were not approaching their respective bounds.

OTA kinetic traces of **CuH_2-CuH_2** and **$CuMe_2$-$CuMe_2$** were fit to the model given by Equation S1:

$$S(t) = \sum_{i=1}^{n} A_i \exp\left(\frac{\sigma^2}{2\tau_i^2} - \frac{t-t_0}{\tau_i}\right)\left(1 - \mathrm{erf}\left(\frac{\sigma^2 - \tau_i(t-t_0)}{\sqrt{2}\sigma\tau_i}\right)\right) \quad \text{(S1)}$$

Here n is the number of decay components included in the fit, and each term corresponds to the analytical solution of a Gaussian instrument response function (IRF) with root variance σ centered at $t = t_0$ convolved with an exponential decay with a time constant of τ_i. The amplitudes A_i and time constants τ_i were taken as variables in the least-squares fits along with the time zero t_0 and IRF width τ_0, giving a total of $2n$+2 variables.

For each OTA dataset, the kinetic trace at each recorded probe wavelength was fit to the above model. Each reported time constant τ_i is the average of the values of τ_i obtained at each probe wavelength across the FWHM (as determined from the values of A_i) of that TA feature. The error bars correspond to the standard deviation of those values. For each XTA dataset, the kinetics were only measured at one probe energy, and thus the reported time constants are simply the values obtained from the fit to that trace. The error bars correspond to the square root of the corresponding diagonal elements of the covariance matrix, i.e. the standard error.

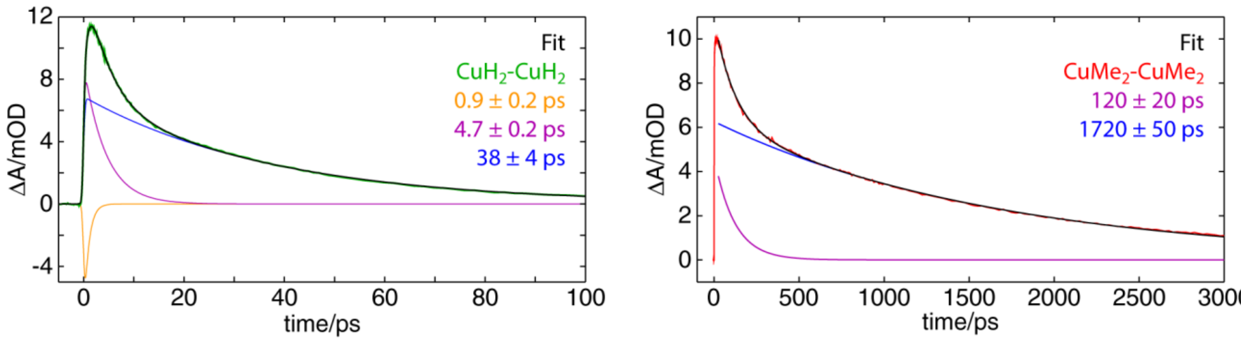

Figure S51. Plot of the OTA kinetic traces of **CuH_2-CuH_2** (top, green) and **$CuMe_2$-$CuMe_2$** (bottom, red) at a probe wavelength of 605 nm and the corresponding fits (black) to the model given by Equation S1. The fit components are plotted in orange, purple, and blue, and the time constants associated with each component are indicated.

The OTA data, fits, and individual fit components for **CuH_2-CuH_2** and **$CuMe_2$-$CuMe_2$** are shown in Figure S51. For **CuH_2-CuH_2**, the data was fit across the entire temporal window (-5 ps to 3 ns) using three components (n = 3). For **$CuMe_2$-$CuMe_2$**, however, the presence of a strong coherent artifact at time zero made it difficult to obtain a stable fit of the early time dynamics across the entire probe wavelength range. But since the ILET time constant and $^3MLCT_0$ lifetime found for this compound are both greater than 100 ps, the early time data could be excluded. An excellent fit across the entire probe wavelength range was

obtained for the time points between 25 ps and 3 ns using only two components (n = 2), and no ISC time constant is reported for this complex. The XTA kinetic trace of **$CuMe_2$** was also fit using the same model with n = 2, as shown in Figure S52. The XTA kinetic trace and the ns OTA trace of **RuH_2-RuH_2** were fit using the same model with only a single component (n = 1), and these fits are shown in Figure S53.

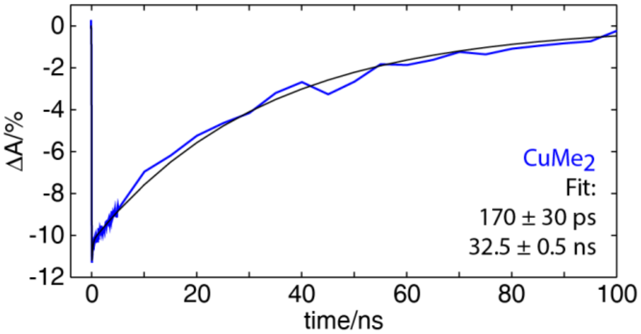

Figure S52. Cu K-edge XTA kinetic trace of **$CuMe_2$** (blue) at a probe energy of 8.984 keV and the corresponding two-component fit (black). The time constants from the fit are indicated.

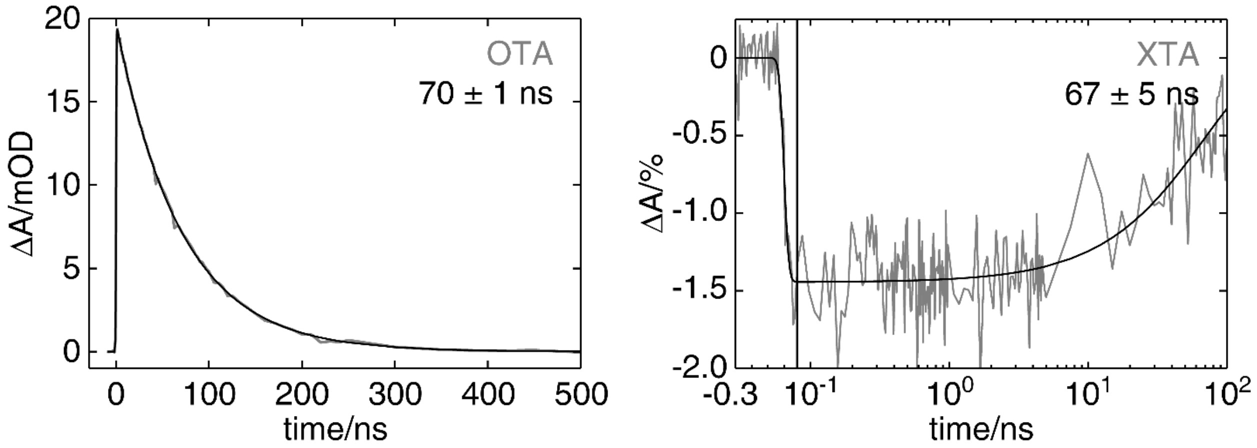

Figure S53. (Left) Nanosecond OTA kinetic trace of **RuH_2-RuH_2** at a probe wavelength of 610 nm (gray) and the corresponding single-component fit (black). (Right) Ru K-edge XTA kinetic trace at a probe energy of 22.126 keV (gray) and the corresponding single-component fit (black). The x-axis is linearly spaced from -0.3 to 0.08 ns and logarithmically spaced from 0.08 to 100 ns; the break is indicated by a solid vertical line.

The OTA kinetic trace of **$CuMe_2$-RuH_2** was fit (Figure 6d) to the model given by Equation S1 with n = 3 plus an additional component corresponding to an exponential decay with a non-impulsive exponential rise time convolved with a Gaussian IRF. This non-impulsive component is given by Equation S2:

$$S_r(t) = A_i\left[\exp\left(\frac{\sigma^2}{2\tau_i^2}-\frac{t-t_0}{\tau_i}\right)\left(1-\mathrm{erf}\left(\frac{\sigma^2-\tau_i(t-t_0)}{\sqrt{2}\sigma\tau_i}\right)\right) - \exp\left(\frac{\sigma^2}{2\tau_r^2}-\frac{t-t_0}{\tau_r}\right)\left(1-\mathrm{erf}\left(\frac{\sigma^2-\tau_r(t-t_0)}{\sqrt{2}\sigma\tau_r}\right)\right)\right] \quad \text{(S2)}$$

Here τ_r is the time constant for the exponential rise, and all other variables are unchanged. This model contains a total of 11 variables. The OTA kinetic trace of **CuH_2-RuH_2** was fit (Figure S54) to the same model as **$CuMe_2$-RuH_2** but with n = 4 and $\tau_4 = \infty$, with a total of 12 variables.

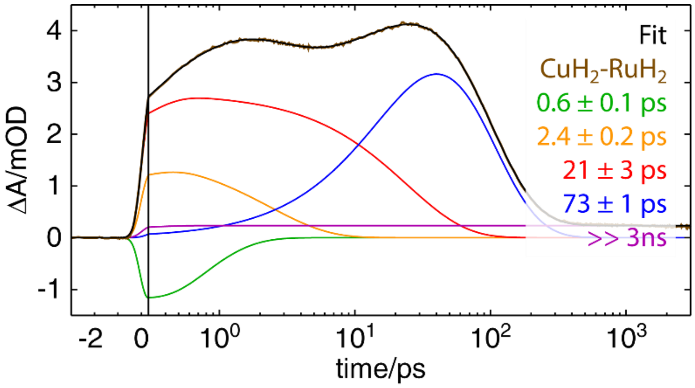

Figure S54. OTA kinetic trace of **CuH_2-RuH_2** (brown) at a probe wavelength of 605 nm, the corresponding five-component fit (black). The components are plotted separately in other colors and the corresponding time constants are given. The *x*-axis is linearly spaced from -3 to 0.3 ps and logarithmically spaced from 0.3 to 3000 ps; the break is indicated by a solid vertical line.

The Cu K-edge XTA kinetic trace of **$CuMe_2$-RuH_2** was fit (Figure 6a) to the sum of a non-impulsive component, given by Equation S2, and an impulsive component, given by Equation S1. The fit was performed with both a single and individually varying time constants for the two components; in either case, the fit converged to give the same lifetime of 1350 ps. The ratio of the amplitudes of the two components (non-impulsive to impulsive) was 1.2 to 1. This may be compared to an expected ratio calculated from the UV-vis absorption spectra of the corresponding symmetric homodinuclear complexes. The extinction coefficients of **$CuMe_2$-$CuMe_2$** and **RuH_2-RuH_2** at 400 nm are 15,400 and 20,800 $M^{-1}cm^{-1}$, respectively, giving an expected ratio of 1.35 to 1, in good agreement with that obtained from the fit.

The Ru K-edge XTA kinetic trace of **$CuMe_2$-RuH_2** was fit (Figure 6b) to either a single impulsive (Equation S1) or non-impulsive (Equation S2) exponential decay convolved with a 120 ps Gaussian IRF. In either case, the time constant was found to be less than 120 ps, indicating that the lifetime of the **$CuMe_2$-RuH_2(III)*** state is shorter than our temporal resolution. For the non-impulsive fit, the rise time was also found to be shorter than our temporal resolution.

The OTA kinetic trace of **CuH_2-$CuMe_2$** at 605 nm was fit (Figure 7) to the model given by Equation S1 in the main text with n = 5, containing a total of 12 fitting parameters. The fit was performed over all probe wavelengths, and the time constants reported in Table 2 are the average of the time constants obtained over the FHWM of the corresponding TA features as described above. In Figure 7, the components assigned to ILET and ground state recovery are grouped together for each copper center, but all components are plotted separately here in Figure S55.

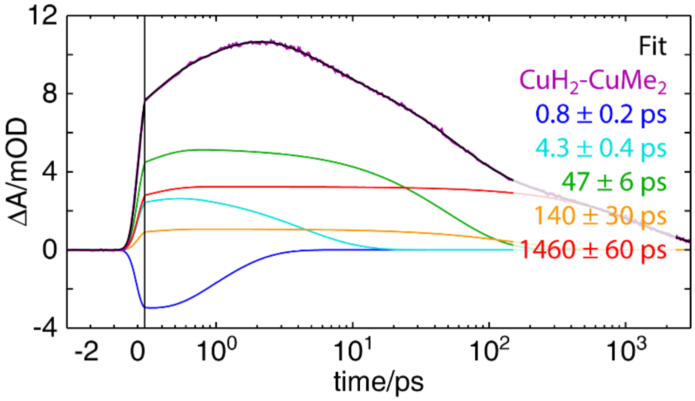

Figure S55. OTA kinetic trace of **CuH_2-$CuMe_2$** at a probe wavelength of 605 nm (purple) and a fit (black) to a linear combination of five decay components plotted separately in other colors. The *x*-axis is linearly spaced from -3 to 0.3 ps and logarithmically spaced from 0.3 to 3000 ps; the break is indicated by a solid vertical line.

To assess the possibility that IMCT does not occur at all in **CuH$_2$-CuMe$_2$**, the same data was also fit to the semi-empirical model given by Equation S3:

$$S(t) = C_1 H(t) \cdot e^{-t/\tau_h} + C_2 M(t) \cdot e^{-t/\tau_m} \quad \text{(S3)}$$

Here *H*(*t*) and *M*(*t*) are the raw OTA kinetic traces of **CuH$_2$-CuH$_2$** and **CuMe$_2$-CuMe$_2$**, respectively, at the probe wavelength being fit, the C_n prefactors are scaling parameters, and the time traces are each multiplied by an exponential decay with a variable time constant to allow the ground state recovery time to vary from that of the corresponding symmetric dinculear complex. The model contains a total of 4 parameters. The fit is shown in Figure S56.

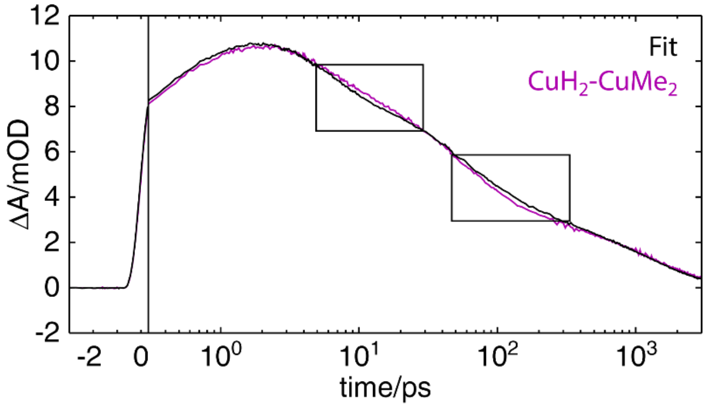

Figure S56. OTA kinetic trace of **CuH$_2$-CuMe$_2$** (purple) at a probe wavelength of 605 nm and the corresponding fit (black) to the electronically decoupled model given by Equation S3. The *x*-axis is linearly spaced from -3 to 0.3 ps and logarithmically spaced from 0.3 to 3000 ps; the break is indicated by a solid vertical line.

While the value of τ_h diverged, leaving the recovery time of the **CuH$_2$** side unchanged, the apparent ground state recovery time τ_a of the CuMe2 side may be calculated from the fit and the lifetime given in Figure S51 according to equation S4:

$$\tau_a = \left(\frac{1}{\tau_m} + \frac{1}{\tau_{\mathrm{MLCT,M}}}\right)^{-1} = \left(\frac{1}{6297\ \mathrm{ps}} + \frac{1}{1720\ \mathrm{ps}}\right)^{-1} = 1351\ \mathrm{ps} \quad \text{(S4)}$$

This value is close to the 1460 ± 60 ps lifetime obtained from the fit shown in Figures 7 and S55, as expected. However, the overall fit does not accurately capture the kinetics observed between 5 and 30 ps and between 50 and 300 ps, as highlighted by black boxes.

11. Numerical modeling of IMCT

To model the contributions to the kinetic traces of CuH2-CuMe2 from IMCT and ground state recovery at both copper centers, we first approximate the initial relative populations of **Cu(II)*H_2-Cu(I)Me_2** and **Cu(I)H_2-Cu(II)Me_2*** immediately following excitation from the extinction coefficients of the corresponding symmetric dinuclear complexes at 415 nm: 15,060 and 12,860 $M^{-1}cm^{-1}$ for **CuH_2-CuH_2** and **CuMe_2-CuMe_2**, respectively. From this ratio, we may assign 53.9% of the initial excited state population to **Cu(II)*H_2-Cu(I)Me_2** and 46.1% to **Cu(I)H_2-Cu(II)Me_2***. We assume the ESA extinction coefficients of for each species are equal, as the ESA corresponds to a ligand radical anion-centered transition. For the sake of simplicity, we also do not consider ILET in this model. Accordingly, the entire **Cu(II)*H_2-Cu(I)Me_2** population decays to the ground state with a time constant of 38 ps. For **Cu(I)H_2-Cu(II)Me_2***, however, we must calculate the branching ratio for the competing IMCT and ground state recovery processes according to Equation S5, where the rates k_x are equal to the reciprocal of the corresponding time constants τ_x:

$$\mathrm{BR} = \frac{k_{\mathrm{IMCT}}}{k_{\mathrm{IMCT}} + k_{\mathrm{MLCT,M}}} = \frac{k_{\mathrm{IMCT}}}{k_{\mathrm{IMCT}} + {}^{1}/_{1720\ \mathrm{ps}}} \tag{S5}$$

Finally, we may calculate the population of **Cu(II)*H_2-Cu(I)Me_2** generated by IMCT as a function of time according to the system of differential equations governing a simple two-step unimolecular mechanism given by Equation S6, where [H] and [M] are the concentrations of **Cu(II)*H_2-Cu(I)Me_2** and **Cu(I)H_2-Cu(II)Me_2***, respectively:

$$\begin{aligned} \frac{d[\mathrm{M}]}{dt} &= -k_{\mathrm{IMCT}}[\mathrm{M}] \\ \frac{d[\mathrm{H}]}{dt} &= k_{\mathrm{IMCT}}[\mathrm{M}] - k_{\mathrm{MLCT,H}}[\mathrm{H}] \end{aligned} \tag{S6}$$

A solution to this system with the initial conditions [H] = 0 and [M] = 1 is given by Equation S7:

$$\begin{aligned} [\mathrm{M}] &= \exp(-k_{\mathrm{IMCT}}t) \\ [\mathrm{H}](t) &= \frac{k_{\mathrm{MLCT,H}}}{k_{\mathrm{MLCT,H}} - k_{\mathrm{IMCT}}}\left[\exp(-k_{\mathrm{MLCT,H}}t) - \exp(-k_{\mathrm{IMCT}}t)\right] \end{aligned} \tag{S7}$$

The total modeled kinetic trace is then calculated as the sum of all the components described above convolved with a 300 fs Gaussian IRF, and the trace is then fit according to Equation S1 with *n* = 2. This process was iterated while varying τ_{IMCT} until the long component had a time constant equal to the 1460 ps lifetime obtained from the fit to the data. This occurred when τ_{IMCT} = 1286 ps. The calculated time trace is plotted in Figure S57a (purple) alongside the individual components: the 38 ps decay of the directly generated **Cu(II)*H_2-Cu(I)Me_2** (green); the 1720 ps decay of **Cu(I)H_2-Cu(II)Me_2*** scaled by 1 minus the branching ratio (red); the 1286 ps decay of **Cu(I)H_2-Cu(II)Me_2*** scaled by the branching ratio (orange); and the non-impulsive exponential growth and decay of **Cu(II)*H_2-Cu(I)Me_2** from IMCT, given by Equation S7 (blue). Importantly, because the ground state recovery time of **Cu(II)*H_2-Cu(I)Me_2** is so fast relative to IMCT, the additional population of **Cu(II)*H_2-Cu(I)Me_2** from IMCT at all times is negligible, reaching a maximum of only 1.7% of the total population at 250 ps as highlighted by the blue arrow. Nevertheless, 57% of the initial **Cu(I)H_2-Cu(II)Me_2*** population relaxes to the ground state through the short-lived

Cu(II)*H_2-Cu(I)Me_2 intermediate on the ns timescale. The two-component fit to the calculated trace is shown in Figure S57b, showing excellent agreement even though the long-time behavior would be more accurately represented by two components of its own.

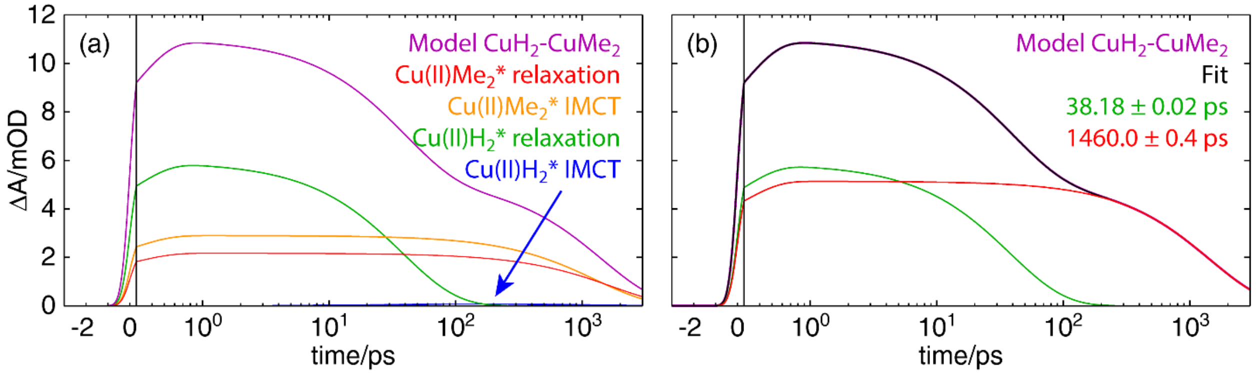

Figure S57. (a) Calculated kinetic trace of **CuH_2-$CuMe_2$** (purple) using the model for IMCT described above. The individual components are plotted in other colors, and the small population of **Cu(II)*H_2-Cu(I)Me_2** arising from IMCT is indicated by the blue arrow. (b) A two-component fit to the calculated trace in panel (a), showing excellent agreement even though the model is comprised of three different components. The *x*-axes are linearly spaced from -3 to 0.3 ps and logarithmically spaced from 0.3 to 3000 ps; the breaks are indicated by a solid vertical line.

12. Sample preparation for semi-empirical OTA modeling

Samples of **CuH_2-$CuMe_2$**, **CuH_2-CuH_2**, and **$CuMe_2$-$CuMe_2$** were simultaneously prepared under identical conditions to obtain the kinetic traces used to fit the **CuH_2-$CuMe_2$** data to the model given by Equations 1, S3, and S7. All samples were prepared in anhydrous acetonitrile in 2 mm fused quartz cuvettes, and the concentrations were adjusted until the peaks near 365 nm in the UV-vis spectra were of equal intensity as shown in Figure S58. This peak was chosen as it is the furthest fully-resolved peak from the MLCT band that we can measure and corresponds to absorption by the bridging tpphz ligand that is expected to be largely independent of the identity of the ligated metals. The samples were then bubbled with dry nitrogen for 2 minutes at the same flow rate and sealed with Parafilm, and OTA spectra were acquired in immediate succession after the entire laser system had stabilized for 24 hours. The total data acquisition time was 4.5 hours (90 minutes per sample).

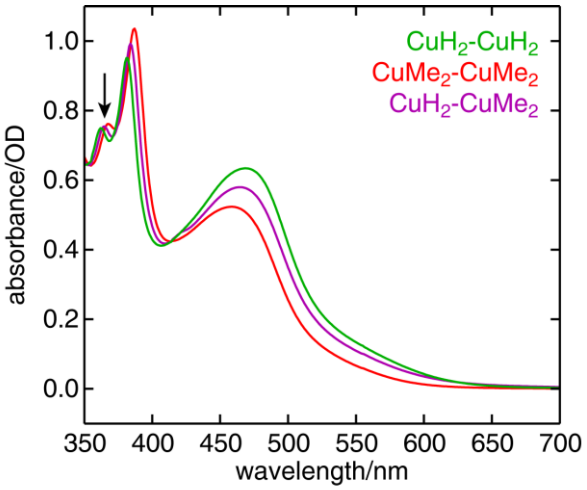

Figure S58. UV-vis spectra of samples used for fitting the **CuH_2-$CuMe_2$** kinetic trace to the models described in the main text and sections 10 and 11 above. The concentrations were adjusted until the peaks near 365 nm indicated by the black arrow were of equal intensity.

13. References

1 D. S. Beaudoin and S. O. Obare, *Tetrahedron Lett.*, 2008, **49**, 6054–6057.
2 J. Bolger, A. Gourdon, E. Ishow and J.-P. Launay, *Inorg. Chem.*, 1996, **35**, 2937–2944.
3 T. A. Vannelli and T. B. Karpishin, *Inorg. Chem.*, 2000, **39**, 340–347.
4 M. Schmittel, U. Lüning, M. Meder, A. Ganz, C. Michel and M. Herderich, *Heterocycl. Commun.*, 1997, **3**, 493–498.
5 L. Kohler, D. Hayes, J. Hong, T. J. Carter, M. L. Shelby, K. A. Fransted, L. X. Chen and K. L. Mulfort, *Dalton Trans.*, 2016, **45**, 9871–9883.
6 Q.-X. Zhen, B.-H. Ye, Q.-L. Zhang, J.-G. Liu, Hong Li, L.-N. Ji and L. Wang, *J. Inorg. Biochem.*, 1999, **76**, 47–53.
7 *Bruker APEX2 Sortware Suite, v2011.4-1*, Bruker AXS, Madison, WI, 2011.
8 G. M. Sheldrick, *SHELXS-97, Program for crystal structure solution*, University of Göttingen, 1997.
9 M. J. Frisch, G. W. Trucks, H. B. Schlegel, G. E. Scuseria, M. A. Robb, J. R. Cheeseman, G. Scalmani, V. Barone, B. Mennucci, G. A. Petersson, H. Nakatsuji, M. Caricato, X. Li, H. P. Hratchian, A. F. Izmaylov, J. Bloino, G. Zheng, J. L. Sonnenberg, M. Hada, M. Ehara, K. Toyota, R. Fukuda, J. Hasegawa, M. Ishida, T. Nakajima, Y. Honda, O. Kitao, H. Nakai, T. Vreven, J. A. Montgomery, J. E. Peralta, F. Ogliaro, M. Bearpark, J. J. Heyd, E. Brothers, K. N. Kudin, V. N. Staroverov, R. Kobayashi, J. Normand, K. Raghavachari, A. Rendell, J. C. Burant, S. S. Iyengar, J. Tomasi, M. Cossi, N. Rega, J. M. Millam, M. Klene, J. E. Knox, J. B. Cross, V. Bakken, C. Adamo, J. Jaramillo, R. Gomperts, R. E. Stratmann, O. Yazyev, A. J. Austin, R. Cammi, C. Pomelli, J. W. Ochterski, R. L. Martin, K. Morokuma, V. G. Zakrzewski, G. A. Voth, P. Salvador, J. J. Dannenberg, S. Dapprich, A. D. Daniels, Farkas, J. B. Foresman, J. V. Ortiz, J. Cioslowski and D. J. Fox, *Gaussian 09, Revision A.02*, Gaussian, Inc., Wallingford CT, 2009.
10 A. D. Becke, *J. Chem. Phys.*, 1993, **98**, 5648–5652.
11 P. C. Hariharan and J. A. Pople, *Theor. Chim. Acta*, 1973, **28**, 213–222.
12 M. M. Francl, W. J. Pietro, W. J. Hehre, J. S. Binkley, M. S. Gordon, D. J. DeFrees and J. A. Pople, *J. Chem. Phys.*, 1982, **77**, 3654–3665.
13 V. A. Rassolov, J. A. Pople, M. A. Ratner and T. L. Windus, *J. Chem. Phys.*, 1998, **109**, 1223–1229.
14 D. Andrae, U. Häußermann, M. Dolg, H. Stoll and H. Preuß, *Theor. Chim. Acta*, 1990, **77**, 123–141.
15 M. T. Kieber-Emmons, *Lumo, Version 0.9b*, Burlingame, CA, 2011.
16 S. I. Gorelsky, *SWizard program, http://www.sg-chem.net*, University of Ottowa, Ottowa, Canada, 2013.
17 S. I. Gorelsky and A. B. P. Lever, *J. Organomet. Chem.*, 2001, **635**, 187–196.
18 S. Miertuš, E. Scrocco and J. Tomasi, *Chem. Phys.*, 1981, **55**, 117–129.
19 M. H. Olsson, U. Ryde and B. O. Roos, *Protein Sci. Publ. Protein Soc.*, 1998, **7**, 2659–2668.
20 U. Ryde and M. H. M. Olsson, *Int. J. Quantum Chem.*, 2001, **81**, 335–347.
21 G. Jennings, W. J. H. Jäger and L. X. Chen, *Rev. Sci. Instrum.*, 2002, **73**, 362–368.
22 X. Zhang, G. Smolentsev, J. Guo, K. Attenkofer, C. Kurtz, G. Jennings, J. V. Lockard, A. B. Stickrath and L. X. Chen, *J. Phys. Chem. Lett.*, 2011, **2**, 628–632.
23 L. X. Chen and X. Zhang, *J. Phys. Chem. Lett.*, 2013, **4**, 4000–4013.
